# From QSAR to deep learning: an interpretable comprehensive pipeline with a read-across approach for mutagenicity prediction *via* the Enalos Cloud Platform

**DOI:** 10.1039/d6ra05786a

**Published:** 2026-09-09

**Authors:** Dimitra-Danai Varsou, Andreas Tsoumanis, Eleonora Marta Longhin, Maria Dusinska, Dimitris G. Mintis, Panagiotis D. Kolokathis, Konstantinos D. Papavasileiou, Iseult Lynch, Georgia Melagraki, Antreas Afantitis

**Affiliations:** a NovaMechanics MIKE Piraeus 18545 Greece varsou@novamechanics.com afantitis@novamechanics.com; b Entelos Institute Nicosia 2102 Cyprus; c NovaMechanics Ltd Nicosia 1070 Cyprus; d Health Effects Laboratory, Department of Environmental Chemistry and Health Effects, NILU – Norwegian Institute for Air Research Kjeller Norway; e School of Geography, Earth and Environmental Sciences, University of Birmingham B15 2TT Birmingham UK i.lynch@bham.ac.uk; f Division of Physical Sciences and Applications, Hellenic Military Academy Vari 16673 Greece; g Department of Pharmacy, Frederick University Nicosia 1036 Cyprus

## Abstract

Assessing the mutagenicity of chemical compounds is essential for ensuring their safe handling and use, thereby minimizing potential health risks. New approach methodologies (NAMs), including computational approaches, provide non-animal alternatives for testing novel materials and chemicals. This study highlights the potential of *in silico* NAMs, which can contribute to the development of novel Safe and Sustainable by Design (SSbD) chemicals and substances by identifying potentially hazardous ones at an early stage. Emphasis is given to the mutagenicity prediction based on data from the Ames (bacterial gene mutation) test curating them to consider stereo-specific input whenever necessary. A consensus strategy integrating different chemical representations (molecular fingerprints, 2D and 3D descriptors and molecular graphs) and modelling methods, *i.e.*, Quantitative Structure–Activity Relationship (QSAR), read-across and deep learning models, is employed to predict the mutagenic profile of chemical compounds. In this course, a XGBoost model is developed based on molecular descriptors and a graph convolutional neural networks model to classify compounds as mutagens and non-mutagens based on the Ames test data. The devised read-across methodology is based on a guided-*k*-Nearest Neighbours scheme (guided-kNN) where two different molecular representations (molecular fingerprints and descriptors) are considered for neighbour selection and predictions generation. The mutagenicity predictions from the three models are integrated in a majority voting scheme to enhance the overall predictive accuracy (83% in external validation) and reduce individual model biases. Interpretation of the descriptors involved in prediction is performed through explainable AI (XAI) methods to provide insight to the mutagenicity mechanism. To enhance the interpretability of the XAI-derived insights and reinforce user confidence in the models' predictions, the involved descriptors are mapped to key events leading to mutations within the Adverse Outcome Pathway (AOP) networks. Apart from the development of reliable and interpretable mutagenicity models, emphasis is given on delivering a pipeline for the generation of 3D descriptors that can be used as the basis for future cheminformatics models. To support transparency and reproducibility of the results of our work, the curated mutagenicity dataset used for modelling is disseminated through the ChemPharos database (https://db.chempharos.eu/datasets/Datasets.zul?datasetID=ds18), the modelling steps are documented following the standardized Modelling Data (MODA) guidelines and the consensus model is freely available *via* the Enalos Cloud platform (https://www.enaloscloud.novamechanics.com/insight/polis/), to facilitate virtual screening of novel compounds.

## Abstract 2

A consensus model integrating QSAR, read-across, and deep learning across multiple molecular representations for Ames mutagenicity, giving interpretable predictions (83% external accuracy), and a reusable 3D descriptor pipeline for SSbD screening.

## Graphical abstract

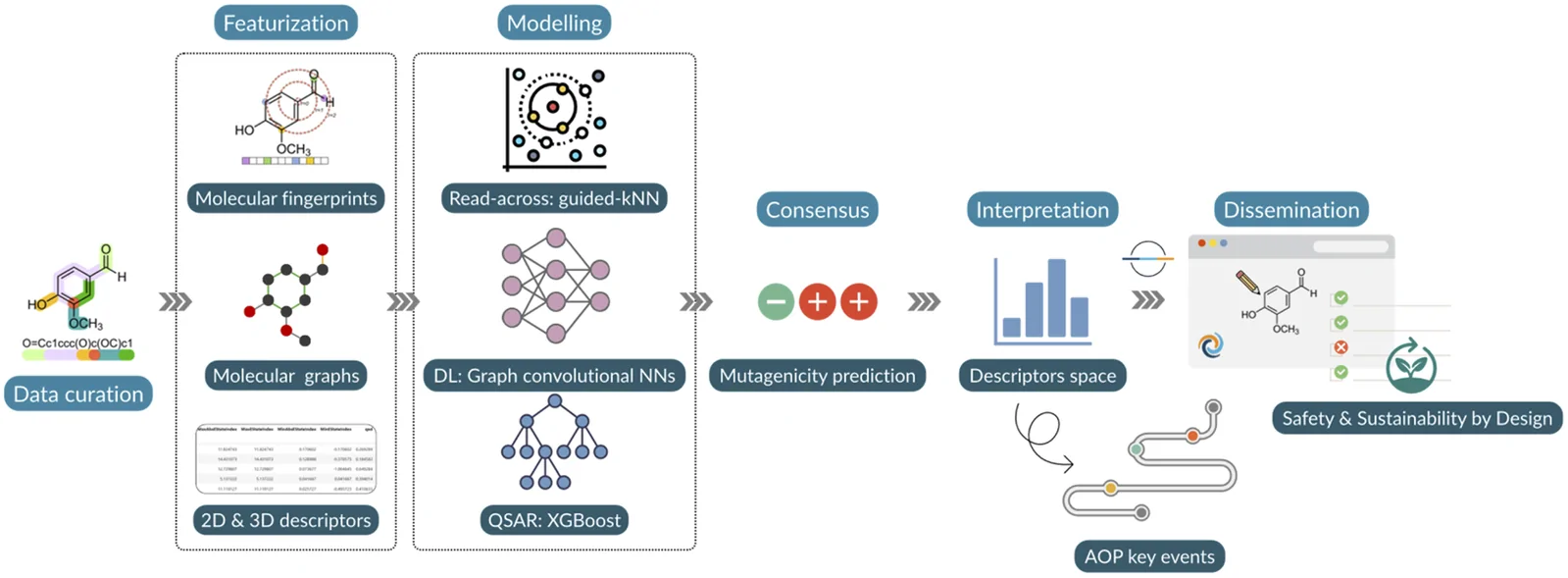

## Introduction

Cheminformatics has transformed the way chemical risk assessment for human health and the environment is conducted, shifting the focus from traditional experimental approaches to more efficient and sustainable alternatives. Since animal-based testing strategies used in chemical safety-assessment are questioned for their limited biological relevance to human effects^1,2^ and are constrained by regulatory frameworks,^3,4^ New Approach Methodologies (NAMs) are emerging as a key component of chemical risk assessment, offering a more ethical, cost effective, and efficient approach to predict chemical hazards, toxicity profiles, and ADMET properties (absorption, distribution, metabolism, elimination and toxicity). NAMs comprise a range of alternative strategies and combinations of methods such as *in silico*, *in chemico*, *ex vivo* and *in vitro* approaches.^2,4^ Beyond addressing ethical concerns, NAMs can shed light on the underlying toxicity mechanisms as they are designed to focus on species-relevant biology,^5,6^ thereby considering the accuracy of the predictions of biological effects.^2^

NAMs including *in silico* approaches are also a cornerstone of the Safe and Sustainable by Design (SSbD) framework, playing a key role in the development of novel compounds with enhanced properties and advanced materials. By integrating computational models into the design and assessment processes, NAMs can address the current chemical safety challenges highlighted in the European Green Deal's zero-pollution ambition^7^ and the Chemicals Strategy for Sustainability (CSS) towards a toxic-free environment.^8^ In this course, the well-established cheminformatics methodologies can greatly support the ongoing research efforts aiming at addressing these challenges, such as the replacement of poly- and perfluoroalkyl substances (PFAS) in products and production processes. PFAS are known for their persistence and adverse effects on both human health and the environment.^9,10^ In 2023 they have been the subject of the largest substances ban proposal in Europe.^11^ Efforts to find effective and safer PFAS-replacements are ongoing in the EU, through activities which include research projects.^12,13^

In the search of novel compounds that can substitute PFAS, computational modelling serves as a useful tool to achieve SSbD objectives.^14^ By predicting the toxicity or other key properties of potential PFAS alternatives, even prior to their actual synthesis, computational models can help identify promising candidates and reveal possible underlying toxicity mechanisms. Therefore, both the time and costs associated with extensive experimental testing can be reduced by prioritizing the most suitable candidates for synthesis and further evaluation.^15^ At the same time, the use of *in silico* methods, *i.e.*, non-animal approaches, supports the 3Rs principles (replacement, reduction, and refinement), contributing to more ethical and sustainable chemical research.^16^

Mutagenicity is a crucial endpoint in a chemical hazard and risk assessment. Mutagenic mechanisms can be broadly summarized in three endpoints: (i) chromosomal aberrations, *i.e.* structural changes in chromosomes (clastogenicity); (ii) numerical changes in the chromosomes (aneugenicity), and (iii) gene or point mutations, *i.e.* changes in the DNA's nucleic acid sequence. Under the European legislation for the Registration, Evaluation, Authorisation and Restriction of Chemicals (REACH), a testing strategy is outlined to cover these different mechanisms. An *in vitro* gene mutation study in bacteria (namely the Ames test) is indicated as the first step to be performed in this testing strategy.^17^ The Ames test is a bacterial reverse mutation assay that detects gene (point) mutations, specifically base pair substitutions (transition and transversions) and frameshift mutations (insertion or deletion of nucleotides), induced by chemical exposure.^18^ It is covered by the OECD Test Guideline (TG) 471. If the result of the test is negative and the test substance is registered in low tonnage, there may be no requirement for further testing. The European Chemicals Agency (ECHA) is now reconsidering mammalian gene mutation test (*Hprt* and *Tk*)^19,20^ to be included in the testing strategies. Gene mutation tests do not detect chromosomal aberrations or aneugenicity, and their predictivity alone to *in vivo* genotoxicity/mutagenicity is therefore limited to only gene mutation endpoint.^21^ For this reason, a recent work from Hjort and colleagues suggests improving the testing strategy for low tonnage substances by supplementing the Ames test with an additional assay, *i.e.* the *in vitro* micronucleus test.^22^ Despite these considerations, partly as a result of the regulatory requirements and testing strategy described above, the majority of mutagenicity data available so far have been produced using the Ames test. As developing robust *in silico* models requires sufficiently large amount of data, for this work we selected the Ames test as a base for the model development.

When it comes to use of resources, the Ames test provides results within 2–3 days,^18^ making it relatively fast compared to other genotoxicity and mutagenicity assays. Nonetheless, when screening large sets of compounds, it can be resource intensive as it requires compound synthesis prior to their evaluation.^23,24^ Therefore, the development of robust and validated *in silico* approaches to predict mutagenicity can facilitate the fast screening of compounds prior to their synthesis, it can help address some of the limitations of experimental testing,^24^ and prioritize compounds for laboratory testing, which is essential for regulatory confirmation.

The mutagenicity endpoint has been assessed *in silico* over the past twenty years by employing various molecular featurizations and modelling approaches. Advancements in machine learning (ML) and deep learning (DL) have evolved the mutagenicity predictions starting from classical quantitative structure–activity relationship (QSAR)-type models relying mainly on molecular descriptors and rule-based structural alerts.^25,26^ Although interpretable, these models are often limited by their training chemical space and cannot generalize beyond their applicability domain.^27^ More recent approaches have shifted towards molecular fingerprints, graph-based representations, and DL architectures, allowing researchers to gain insights on the molecular structural patterns governing mutagenicity.^23^

Starting from conventional ML approaches, Hansen *et al.*^28^ first developed a benchmark dataset collecting mutagenicity data from Ames test and next employed different ML algorithms (Random Forest (RF), Support Vector Machines (SVM), *k*-Nearest Neighbours (kNN) *etc.*) to predict mutagenicity based on molecular descriptors, reaching an area under the curve (AUC) score of 0.86 in internal validation. Xu *et al.*^29^ also employed classical ML approaches including kNN, SVM, classification trees *etc.* to classify compounds as mutagens or non-mutagens based on molecular fingerprints reaching an AUC score of 0.97 in external validation. Chu *et al.*^25^ evaluated multiple ML methods on the Xu *et al.*’s curated dataset and their RF model exhibited the highest accuracy (0.79) and AUC (0.84). Similarly, Vukovic *et al.*^30^ developed the aiQSAR approach combining fingerprint similarity and descriptors, achieving an accuracy score of 0.81 in internal validation. The VEGAHUB platform also offers four models and one consensus one for the compound mutagenicity assessment based on Ames test data. The approaches include kNN, statistical-based and structural alerts-based modelling.^31^

Graph Convolutional Neural Networks (GCNNs) and Graph Neural Networks (GNNs) are DL approaches that directly operate on molecular graphs without requiring explicit descriptor calculations, enabling a more data-driven learning process. Peng *et al.*,^32^ based on the Xu *et al.*^29^ dataset, developed an enhanced Graph Isomorphism Network (GIN) achieving an accuracy score of 0.84 in external validation. Hung *et al.*^33^ developed GCNN models based on a large-scale curated set of 17 643 compounds, achieving an accuracy score of 0.84 and an AUC of 0.87 in external validation. Shi *et al.*^34^ developed an image-based Convolutional Neural Network (CNN) model to predict mutagenicity-among other ADMET properties-based on the data of Xu *et al.*,^29^ achieving an accuracy score of 0.91 in external validation. Given the possibility that we might omit many studies related to the computational assessment of mutagenicity, we encourage interested readers to refer to the Tran *et al.*'s^24^ recent review paper.

Despite this promising progress, several challenges remain, starting with the inconsistencies in dataset preparation, such as reported duplicate entries,^25^ class imbalance, and variations in featurization techniques, that make it difficult to make direct comparisons across models. In addition, many of the studies found in the literature primarily assess performance using internal validation^30,35,36^ and do not report performance on external test sets, raising uncertainties regarding models' generalizability. Nonetheless, computational integrated approaches combining different ML and DL approaches may be a promising path towards the development of robust and generalizable mutagenicity prediction models.

Finally, another challenge that must be considered during the development of predictive models for the *in silico* assessment of chemical compounds is their stereochemistry, *i.e.*, the spatial three-dimensional arrangement of their atoms. In brief, considering that biological targets such as DNA, receptors, and enzymes are chiral entities themselves or have specific spatial conformations, their interaction with small molecules is highly selective as it depends on stereochemistry.^37–39^ For example, two stereoisomeric forms of a drug (identical in molecular composition) can exhibit different pharmacological activity (active/inactive), potency or even toxicity (safe/toxic)^38–41^ and in some cases drugs are therefore marketed as single enantiomers rather than as racemic mixtures.^37,41^ Similarly, in the field of materials science, the physicochemical, mechanical, functional and other properties of polymers are strongly influenced by their stereoregularity and overall spatial arrangement.^42–45^ For this reason, a thorough understanding of stereochemistry is essential for both the development of new drugs and advanced materials, and for the development of reliable and meaningful predictive models that contribute to SSbD. Mutagenicity is among the endpoints that may be influenced by stereochemistry^46–48^ thus, efforts should be made to incorporate stereochemical descriptors into the development of computational approaches to predict mutagenicity.

This work aims to develop computational methods that can be used for the virtual screening of small molecules, accelerating the processes of drug design and material development, excluding non-promising candidates in the early stages of their study, and saving experimental resources. A synergistic approach is followed including data curation, modelling, validation and interpretation, standardized reporting, and model and data dissemination. In this course, a workflow for the generation of 3D descriptors was proposed, a ML QSAR model and a DL model were developed, along with a novel guided-kNN methodology that can be considered a read-across approach. Read-across approaches are data-driven methods which have been developed as alternative non-testing strategies for the data-gap filling of physicochemical, human health and/or environmental properties based on structural similarities to well-characterized compounds.^49,50^ In this work the developed read-across methodology considers two different molecular featurization methods, first for calculating similarity between chemical compounds and then for making reliable predictions.

The Ames test data used for model development to predict mutagenicity were sourced from public databases, and for each methodology or model developed, different molecular featurization techniques were employed, considering stereochemistry information whenever possible, to achieve richer molecular representation by merging complementary information and thus, improved predictive performance. The enriched data are made available in a ready-for-modelling format through the ChemPharos database,^51^ a database that supports cheminformatics research and development. At the final stage of the study, predictions from the three models (QSAR, read-across and DL) were integrated into an ensemble scheme to combine the collective knowledge from different models and chemical representations, and thus to produce more reliable results. The models are applied on a set of chemicals that are components of candidate PFAS-free coating formulations to assess their mutagenic potential. Emphasis was placed on interpreting the predictions to highlight the molecular fragments and functional groups responsible for their adverse effect. The consensus model can be accessed *via* the Enalos Cloud platform,^52^ to support virtual screening of novel compounds.

## Data

In this study, model development is based on the Ames test mutagenicity dataset^29^ directly retrieved by the Therapeutics Data Commons (TDC)^53^ platform. TDC is an open science initiative that provides a comprehensive platform of curated datasets, tools, and benchmarks for machine learning and data-driven research in therapeutics, including drug discovery, development, and repurposing. Regarding the Ames test mutagenicity dataset^29^ additional information (experimental conditions, included bacteria strains, metabolic activation) was not available. TDC offers for each dataset, split functions in Python to support training of ML models. In this course, the TDC's protocol is followed for random data splitting into training (70%), validation (10%) and test (20%) subsets, for the development of our ML and DL classification models.^53^ Training and validation sets are used for model development and hyperparameters tuning, whereas the test set is used to assess the predictability of the models.

### Molecular featurization

To transform the structural characteristics of the available compounds into representations that can be used by computational tools for model development, different molecular featurizations are employed:

• Molecular descriptors represent the structural features and properties of molecules by converting their characteristics into numerical values. These numerical representations can then serve as inputs for various computational techniques.^54^ In this work, Mold2 and RDKit descriptors are employed. Mold2 software, developed by the National Center for Toxicological Research (NCTR), was used to obtain 777 unique molecular descriptors that capture the properties of small molecules, based on their 2D chemical structure.^55^ RDKit software calculates 210 descriptors that encode physicochemical, topological, and structural properties of compounds, again based on their 2D structure.^56^ Additionally, 11 descriptors based on the 3D molecular structure (3D conformers) are calculated using RDKit.^57^

• Molecular fingerprints are binary vectors where the different bits indicate the presence or absence of different fragment features within the molecule.^58^ In this work, Morgan fingerprints are used (also known as extended-connectivity fingerprints, ECFP)^59^ that encode the molecular structures by iteratively assessing the atoms local environment.^60^ In fact, count fingerprints are used to represent the frequency of nonzero counts for substructures in the molecules.

• Graph representations: molecules can be also represented as undirected graphs, where atoms are the graph nodes and bonds are the edges.^23,33^ This mathematical representation allows for the development of graph convolutional neural networks (GCNNs) that can be trained to predict molecular properties and/or activity. In this work the DeepChem library^61^ is employed for the compounds featurization as chemical graphs and the development of GCNNs.

### Data pre-processing and curation

Compounds, as imported from the TDC, are encoded in SMILES (Simplified Molecular Input Line Entry System) notation, which represents molecular structures as ASCII character strings. The compounds in the three subsets are sanitized, standardized (normalization of functional groups, retain largest organic fragments) to reduce noise in the cheminformatics analysis, thereby enhancing model performance and interpretability. Later pre-processed molecules are transformed into canonical SMILES using RDKit to ensure consistency and eliminate representational redundancies. The sets are curated to remove duplicate entries within each subset and between different subsets, and the pre-processed dataset comprises 7255 chemical compounds from which 3951 are mutagens/Ames positive – class 1, and 3304 non-mutagens/Ames negative – class 0.

Furthermore, as the three-dimensional arrangement of atoms in a molecule may control its mutagenic potency and related properties, curated sets are restricted to focus only on the compounds with explicitly defined stereochemistry. The RDKit EnumerateStereoisomers module^62^ is used to search for possible stereoisomers for each one of the compounds of the training, validation and test sets. Compounds that yield multiple stereoisomers due to unassigned stereocenters, are filtered out. More specifically, out of the total 7255 molecules, 5362 have a defined stereochemical assignment, meaning that the mutagenicity endpoint corresponds precisely to a single structure rather than an ambiguous spatial arrangement or a racemate. However, among the featurization methods applied for encoding the structural characteristics of the molecules, only the 3D descriptors take stereochemistry into account. For this reason, an additional filtering of the chemical compounds is performed so that the 3D descriptors correspond as closely as possible to the input compounds, excluding those with ambiguous stereochemistry. Therefore, the models that employ 3D descriptors are trained and validated exclusively on the curated sets. Such a strict filtering process may potentially exclude certain scaffolds that could provide better chemical space coverage and class balance. Nevertheless, for the development and evaluation of the GCNN model, which encodes only atom connectivity (and not the three-dimensional structure), all 7255 molecules of the initial set are used. Thus, we consider that, through the combination of molecular featurization methods, the data space is adequately covered. The datasets used for model development are summarized in Table 1. The data curation and featurization process is schematically presented in Fig. S1.

**Table 1:** Summary of the mutagenicity dataset from the Ames test used for model development^63^

Subset	All	Mutagens [1]	Non-mutagens [0]
**Initial set**			
Training	5076	2741	2335
Validation	726	415	311
Test	1453	795	658
Total	7255	3951	3304

**Curated set**			
Training	3724	2090	1634
Validation	549	325	224
Test	1089	601	488
Total	5362	3016	2346

It should be noted that, although the same training and validation sets were not used for model development across the three methods described in the following sections, in order to ensure fair comparisons during external model validation, the results are presented on the curated test set.

## Methods

The computational assessment of mutagenicity is performed by developing three different *in silico* approaches that include read-across, DL, and QSAR modelling, leveraging various data and featurizations, as described in the following paragraphs. The produced models are later incorporated into a consensus modelling scheme accounting the majority of their predictions, to provide more robust and reliable findings, allowing to combine the models' strengths and lessen their weaknesses.^64,65^ To render these models more FAIR (Findable, Accessible, Interoperable, Reusable) their metadata are also reported^66^ through the standardized Modelling Data (MODA) guidelines proposed by the European Materials Modelling Council (EMMC) using the Easy-MODA tool.^67^ The MODA report is available in the SI files of this publication.

The models are implemented using open-source Python libraries, including PyTDC v0.3.6 (data retrieval),^53^ deepchem v2.8.1 (featurization, GCNN model development),^61^ Keras and TensorFlow v2.15.0 (GCNN model development), RDKit v2024.3.6 (standardization, 2D and 3D featurization, similarities calculation, enumeration of stereoisomers, conformers generation),^68^ pandas v2.1.4 (data manipulation),^69^ numpy v1.24.4, and scikit-learn v1.4.1 (predictive data analysis).^70^ For the QSAR models, feature engineering is carried out in KNIME Analytics Platform,^71^ where molecular featurization is performed with Enalos+^72^ Mold2 node. To ensure compatibility with Mold2's strict SDF parsing requirements, molecular structures were first converted with Open Babel v3.1.1 (ref. 73) into a format fully accepted by Mold2. The QSAR model development, optimization and web implementation is performed through Isalos Analytics Platform using the normalization, classification-AutoML and applicability domain functions.^74^ Finally, the SHAP analysis is performed in Python using shap v0.46.0 library.^75^

### Guided-kNN

The *k*-Nearest Neighbours (kNN) algorithm has been one of the most widely used and easily understandable machine learning algorithms over time, due to its simplicity with respect to its ability to produce reliable results.^76^ In its simplest and most intuitive form, classification is based on the majority vote of the class of the *k*-nearest neighbours of the query sample. For regression tasks, the algorithm performs in a similar manner. The key components of the algorithm are:^76,77^

1. The distance calculation, which also quantifies the similarity of the neighbours,

2. The number (*k*) of neighbours used in the prediction, and

3. The voting scheme for the final prediction.

Essentially, an explicit model is not constructed; instead, predictions are adaptable to the training data (lazy/instance-based classifier).^77^ The kNN's simplicity gives researchers flexibility in terms of potential variations of the classic algorithm. More specifically, numerous variations of kNN have been proposed and are widely used, targeting specific weaknesses (*e.g.*, improving distance calculations, selecting *k*-neighbours, reducing time complexity, *etc.*). These variations modify or optimize one or more of the algorithm's key components to produce even more reliable predictions and/or to make it more robust.^76–79^

A comparative analysis of kNN variations in the field of disease prediction is presented in the article by Uddin *et al.*^78^ Broader variations of the nearest neighbours algorithm also include methodologies based on the local density of samples. In such variations, the number of neighbours is not fixed but is selected based on one or more thresholds or radii-a similarity limit that samples should meet in order to be considered as neighbours.^50,80,81^

In this work, emphasis is given to the algorithms interpretability by integrating problem-oriented knowledge within the neighbour selection process. In detail, for our cheminformatics analysis the use of chemical fingerprints for the similarity search step instead of distance calculation based on molecular descriptors is considered a better practice, as fingerprints are focused on structural patterns and connectivity and thus are particularly advantageous for comparing molecules based on structural motifs or scaffold similarity.^82,83^ Nonetheless, the structural, topological, physicochemical, and electronic properties encoded in the molecular descriptors^56^ should not be neglected during prediction as they capture subtle differences between molecules^84^ and their use in combination with the chemical fingerprints can leverage the limitations imposed by the latter. For this reason, we propose a combination of the fingerprint similarity of the query molecules with their properties, by altering the steps 1 and 3 of the kNN algorithm, as follows:

1. Each molecule is encoded with a chemical fingerprint (count-based Morgan fingerprints in this case) and Tanimoto similarity^85^ is calculated between query molecules and training set molecules (eqn (1)).1
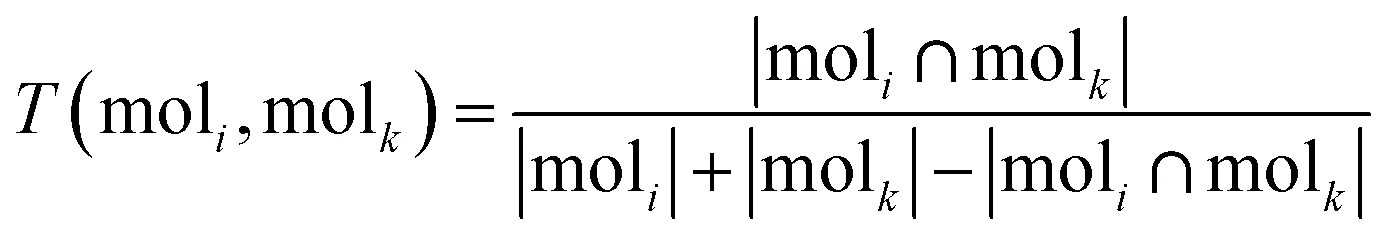
where *T*(mol_*i*_, mol_*k*_), the Tanimoto similarity between the *i*th query molecule and the *k*th molecule from the training set, |mol_*i*_∩mol_*k*_|, the number of common features between the fingerprints of the *i*th query molecule and the *k*th training molecule (bits set to 1 in both fingerprints in mol_*i*_ and mol_*k*_), |mol_*i*_|, the total number of bits set to 1 in fingerprint of mol_*i*_, |mol_*k*_|, the total number of bits set to 1 in fingerprint of mol_*k*_.

2. The *k*-neighbours are selected based on their Tanimoto similarity ranking.

3. The class prediction for the query molecule is the weighted majority vote of the *k*-neighbouring molecules (eqn (2)). Weights are proportional to the inverse distances between these neighbours (eqn (3)), which are calculated using their molecular descriptors.2
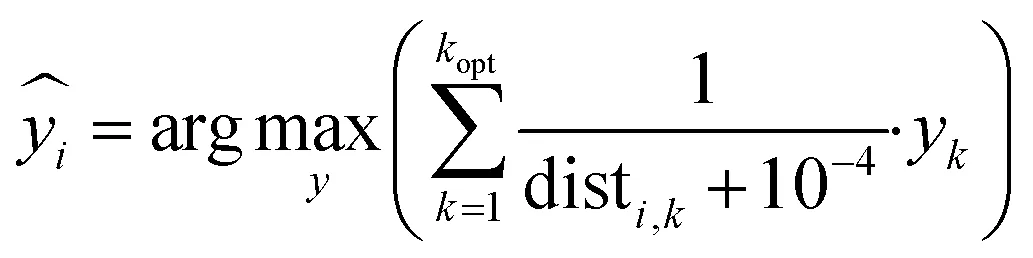
where *ŷ*_*i*_, the predicted class for the *i*th query compound, *k*_opt_, the number of selected training neighbours based on their Tanimoto similarity, dist_*i*,*k*_, the Euclidean distance of the *i*th query compound from the *k*th training neighbour.

3 dist_*i*,*k*_ = ‖***x***_*i*_ − ***x***_*k*_‖

where ***x***_*i*_ and ***x***_*k*_, the descriptor values of the *i*th query compound and its *k*th training neighbour.

The proposed variation of the kNN algorithm results in a hybrid method where vicinity is driven by adequate similarity criteria within the read-across framework. Read-across is a cheminformatics and nanoinformatics strategy for estimating the properties of a target chemical using data from similar-source chemicals,^49,86^ with one of the critical steps being the identification of similarity. Fingerprints aid this process by quantifying structural similarities through metrics like the Tanimoto coefficient, grouping chemicals into similarity clusters for refined read-across predictions by integrating the descriptors-based data. Leveraging fingerprints thus makes read-across a more robust, scalable, and data-driven method, particularly useful in regulatory and industrial applications like chemical risk assessment. In addition, depending on the type of data and the system under investigation stakeholders can tailor the guided-kNN approach to their specific problem by adopting different methods for neighbour selection (step 1) and voting scheme (step 3), *e.g.*, in the nanoinformatics field the multi-perspective characterization data of nanomaterials can be considered in the definition of similarity and within the weighted majority vote prediction. Other similarity and/or distance metrics can be also employed *e.g.*, Dice similarity and Manhattan distance, respectively.

#### Modelling workflow

**Fig. 1:**
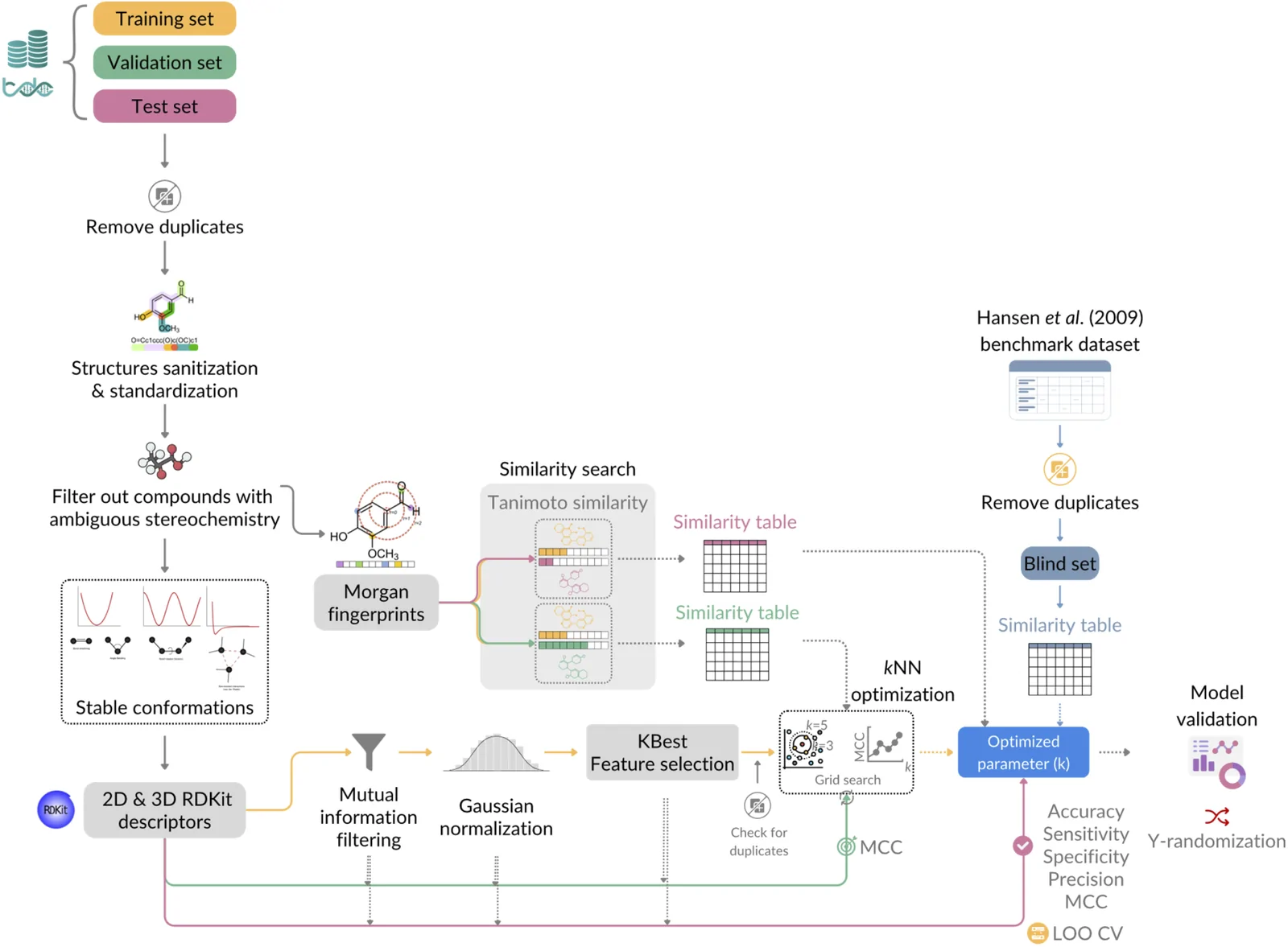
Schematic workflow of the guided-kNN pipeline development.

For the setup of the guided-kNN pipeline (Fig. 1), the curated training, validation and test sanitized and standardized compounds are featurized using count-based Morgan fingerprints^87,88^ with a radius of 4 and a size of 2048 bits using RDKit.^60^ Based on the fingerprints, molecular similarities between training-validation and training-test compounds are quantified with Tanimoto index. Next, for all data subsets 2D and 3D RDKit descriptors are calculated.^56^ Especially, to calculate 3D molecular descriptors, each molecule is initially pre-processed by adding hydrogen atoms explicitly. A diverse set of conformers (atomic coordinates) is then created using the Experimental-Torsion Distance Geometry (ETKDG) algorithm,^89,90^ which combines the distance geometry approach and knowledge-based torsion-angle preferences to guarantee chemically reasonable geometries. In particular, 20 conformers are created per molecule to take into consideration molecular flexibility and reduce the possibility of missing energetically favourable or relevant conformations. Each conformer is subsequently optimized using the Universal Force Field (UFF)^91^ to refine its geometry. Finally, the lowest potential energy conformer is selected as the representative structure for descriptor calculation, so that the calculated 3D descriptors are a reference to a thermodynamically stable conformation.

The descriptors pool is further refined to keep the ones that are relevant to the mutagenicity endpoint. To this end, mutual information filtering^92^ is performed on the training set to keep the 120 more informative descriptors for predicting the endpoint. The remaining descriptors are standardized with *z*-score (Gaussian)^93^ transformation to guarantee that they participate equally to the analysis. KBest feature selection^94^ is also performed to the training set to filter out less informative features based on the descriptors' ANOVA *F*-values and keep the top 50 more relevant ones. It is noted that the filtered out descriptors from the training set are also excluded from the validation and test sets and the standardization parameters derived from the training set are subsequently applied to the validation and test sets, to maintain consistency.

Later, the calculated similarities based on Tanimoto coefficient, calculated using count-based Morgan fingerprints, and the refined descriptor sets are fed to the guided-kNN algorithm as described above. To optimize the number *k* of neighbours, the guided-kNN is included in a grid search scheme: initially, the similarities between the training and validation compounds are considered, and predictions are calculated for different *k* values ranging from 3 to 20. For each *k*, the predictions' Matthews correlation coefficient (MCC, eqn (4)) value is recorded and the *k* value returning the highest MCC (*k*_opt_) is selected for the final guided-kNN model. The model's ability to produce reliable predictions under real-world conditions is evaluated with the curated test set compounds.

### Graph convolutional neural networks

CNNs are widely used in processing and modelling images, speech, and time-series data because convolutional layers take advantage of hierarchical patterns and extract high-level features from input data, leading to powerful representational capabilities.^95,96^ Early layers in CNNs detect small-scale local patterns, while deeper layers capture larger, more abstract features. GCNNs extend CNNs to graph-structured data by leveraging the inherent graph topology. In the context of cheminformatics, GCNNs combine information from neighbouring nodes—bonded atoms in a molecule—in a convolutional manner, allowing for effective learning directly on molecular graphs.^95^

The GCNN model for the mutagenicity class prediction is developed with the open-source DeepChem package^61^ and the TensorFlow framework *via* Keras. The model treats each molecule as a graph and processes these graphs through multiple GraphConv layers (their size and activation are subject to optimization) that implements the graph convolution. Each GraphConv layer is followed by BatchNormalization to stabilize training and dropout to reduce overfitting. A GraphPool layer then performs max pooling over the feature vectors of atoms in a neighbourhood.

The pooled features of the last GraphConv layers are passed through a fully connected dense layer with an activation function (subject to optimization) for further refinement. Normalization and dropout layers are introduced at this stage, as well. A GraphGather layer then aggregates the entire graph's representation. Finally, a dense layer reshapes the output (*via* reshape) and applies a softmax activation function, yielding probability distributions over the two classes. A probability closer to 1 is assigned to Ames positive compounds and a probability closer to 0 to Ames negative ones. The model is trained using the AdamW optimizer-combining adaptive learning rates with weight decay- and a categorical cross-entropy loss to update the network's weights.

It should be noted that a Python generator function is used in order to convert the featurized molecules into numpy arrays that can be used as input to the GraphConv, GraphPool, and GraphGather layers.

#### Modelling workflow

**Fig. 2:**
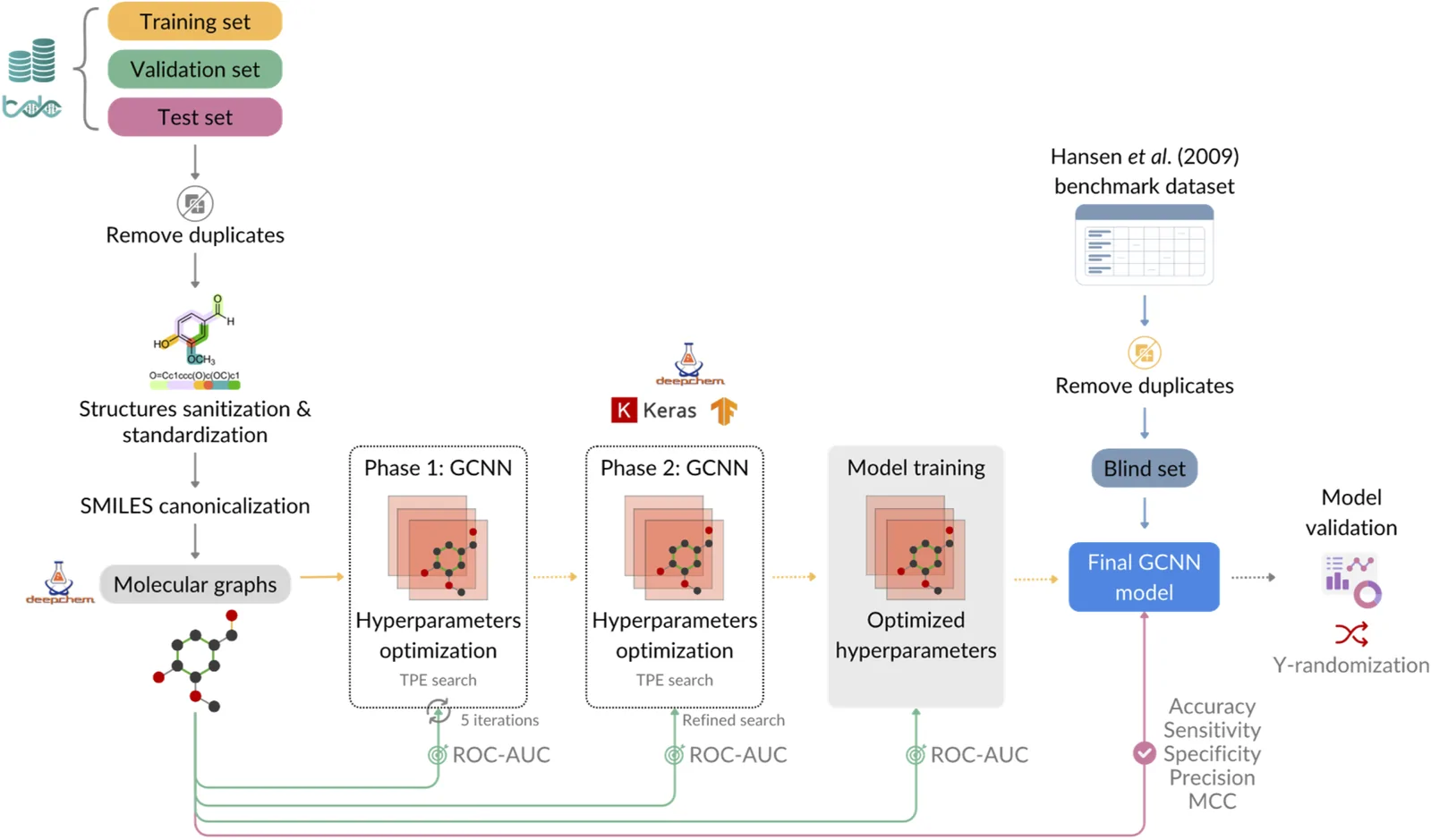
Schematic workflow of the GCNN model development.

The complete GCNN model development workflow is depicted in Fig. 2. Molecular featurization is performed on the initial training, validation, and test compounds starting from their canonical SMILES strings using the DeepChem ConvMolFeaturizer^97^ that implements Duvenaud graph convolutions.^96^ Then hyperparameters tuning is performed following a two-phase strategy using the hyperopt library^98^ and the Tree-structured Parzen Estimator (TPE) algorithm.^99^ In this course the graph convolutional layer sizes, dense layer size, batch size, dropout rate, learning rate, weight decay, and activation function are iteratively varied to find the combination maximizing the receiver operating characteristic curve (ROC-AUC) score of the validation set. More specifically, in the first phase, a broad search space is explored (see Table 2) over 15 epochs, performing 80 evaluations (hyperparameter configurations) and repeating the process five times to validate stability. During training early stopping (with a patience value of 3 and min_delta equal to 10^−4^) is applied to prevent overfitting by customizing the DeepChem validation callback class.

**Table 2:** Parameters optimization search space for the GCNN model

Parameter	Search space (phase 1)
Graph convolution layers	[64, 64], [128, 64, 32], [64, 64, 64], or [256, 128, 64, 32]
Dense layer size	64, 128, or 256
Batch size	16, 32, or 64
Dropout	*X* ∼ *U*(0, 0.5)
Learning rate	log(*X*) ∼ *U*(−8.0, −3.0)
Weight decay	10^−5^, 10^−4^, or 10^−3^
Activation function	Hyperbolic tangent (tan *h*) or rectified linear unit (ReLU)

Hyperparameters that consistently appear in the results of the five iterations of the first phase are then kept fixed, and the rest of the hyperparameters are refined in the second phase. In this phase the search space is narrowed for the remaining hyperparameters, and tuning is performed over 30 epochs with 50 evaluations, maintaining the same early stopping criteria. The two-phase approach provides both stability and optimal hyperparameter selection, leading to a well-generalized GCNN model. The hyperparameters leading to higher ROC-AUC score on the validation set during the second phase along with the fixed hyperparameters of the first phase are used for training the final model using 30 epochs.

### QSAR modelling

QSAR is a well-established *in silico* discipline that is widely used in drug discovery and material design applications. Different ML and DL algorithms have been applied to correlate structural and physicochemical descriptors to compounds biological activity and/or toxicity and are able to predict the endpoints of untested chemicals.^100^

#### Modelling workflow

**Fig. 3:**
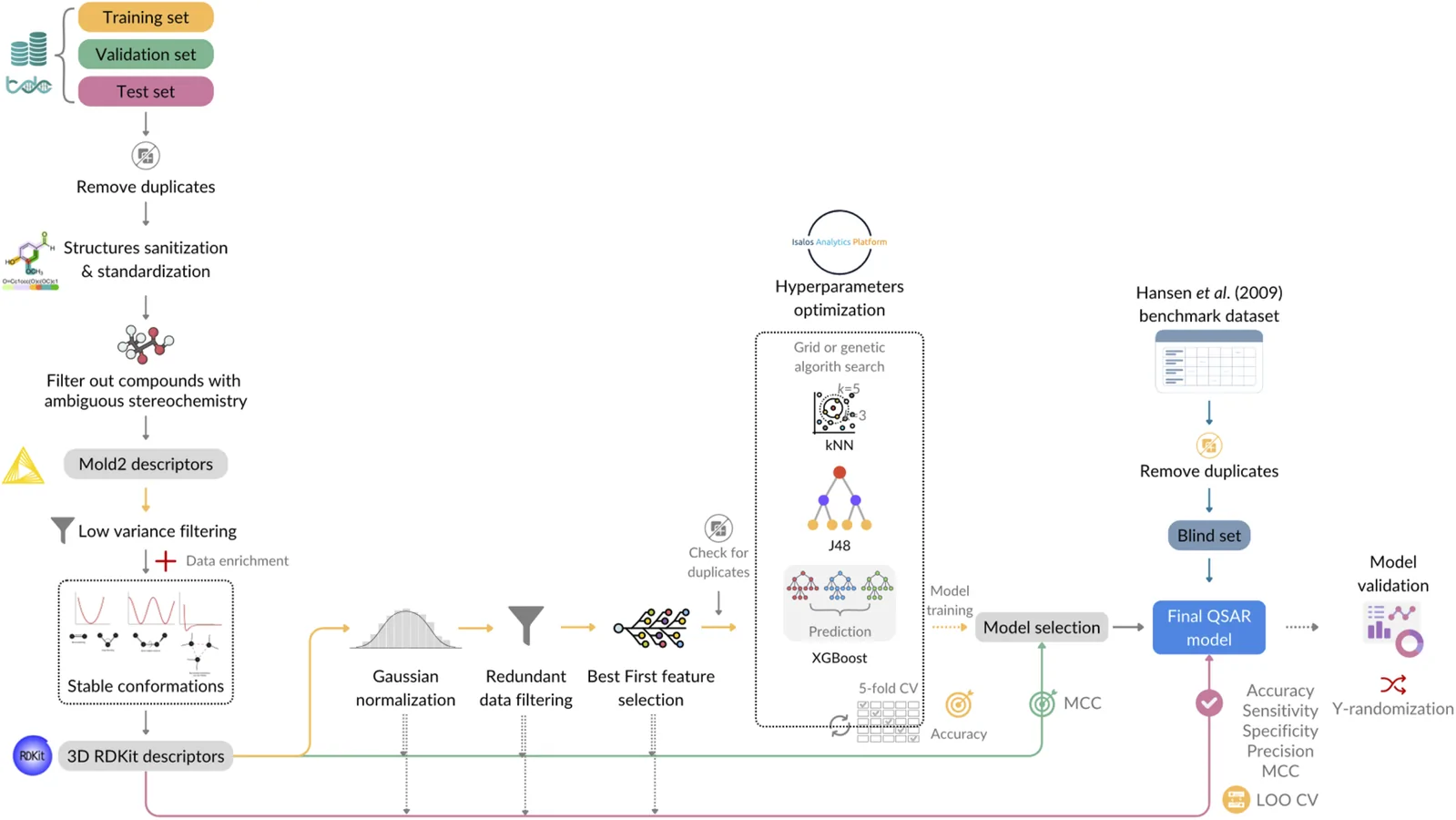
Schematic workflow of the QSAR model development.

The QSAR model development workflow is performed in KNIME and Isalos Analytics Platform for classifying compounds as either Ames positive or negative using 2D Mold2 descriptors^55^ and 3D RDKit descriptors (see Fig. 3). First training, validation, and test molecules are generated from the sanitized and standardized curated structures using RDKit and OpenBabel, and Mold2 descriptors are calculated. Data is then pre-processed to exclude non-informative descriptors, mitigate overfitting, and improve the algorithms' performance. For this reason, a low-variance filter is applied to the training data using a minimum variance threshold of 0.2 for the input descriptors. At this point, the 3D RDKit descriptors calculated before are also included in the three subsets to further enrich the pool of available features. The descriptors are then standardized using the *z*-score method,^93^ to ensure their equal contribution to the analysis. The same standardization function is later applied to the validation and test sets.^101^ The available data are further refined by filtering-out the descriptors with redundant data by removing descriptors where the number of samples of the unique values falls below an 80%.^72^ Next, feature selection is performed on the training data using the BestFirst method coupled with the Correlation-based Feature Subset (CFS) evaluator to select a reduced set of descriptors before developing the predictive models.^77^

After data preprocessing, hyperparameter tuning takes place in Isalos Analytics Platform using the included AutoML function. Three ML algorithms are employed and optimized for their ability to classify compounds as either Ames positive or negative. The tested algorithms include decision tree-based models (the implementation of the C4.5 decision tree algorithm-known as J48,^77^ and the Extreme Gradient Boosting (XGBoost) algorithm^102^), and the kNN algorithm.^103^ For each ML algorithm, hyperparameter tunning is performed (see Table 3) following either a grid or a genetic algorithm search strategy. In the case of the genetic algorithm a population of 50 chromosomes (potential solutions) was set for 20 generations (iterations) in all cases. Crossover probability and mutation rate were set to 0.3 and 0.1, respectively. For each parameter combination, a ML model is developed using a 5-fold cross-validation (CV) scheme and the 5-folds accuracy values (eqn (2)) are recorded and averaged into a single value. The hyperparameter combination yielding the highest average accuracy is selected for each optimized model.

**Table 3:** Hyperparameters optimization search space for the tested ML models

ML algorithm	Hyperparameters	Search space [lower, upper values]; step
J48 (grid search)	Maximum depth	[2, 10]; 1
	Minimum sample split	[5, 20]; 2
XGBoost (genetic algorithm)	Maximum tree depth	[3, 15]; 2
	Learning rate (eta)	[0.10, 0.30]; 0.05
	Alpha	[0, 5]; 1
	Lambda	[0, 5]; 1
	Gamma	[0, 5]; 1
	Minimum child weight	[1,10]; 2
	Subsampling rate	[0.5, 1.0]; 0.1
kNN (grid search)	Number of neighbours (*k*)	[3, 20]; 1

Finally, the three ML models are built using the optimized hyperparameters and the training set, and they are then applied to the validation set. The best-performing model, based on the accuracy statistics (eqn (4)–(8)), is selected as the final QSAR model and it is applied on the test compounds to assess its generalization and predictive performance.

### Model validation

The generated models are externally validated using the test set compounds that are not involved in model development. The test set, like the validation set, is randomly selected from the complete dataset (Table 1), allowing for a fair evaluation of the developed models. In this course, the number of correct predictions and the number of misclassifications is considered, by calculating the MCC, accuracy, sensitivity, specificity, and precision metrics using the formulas presented in Table 4.

**Table 4:** List of metrics used for the validation of the developed models

Matthews Correlation Coefficient (MCC)	
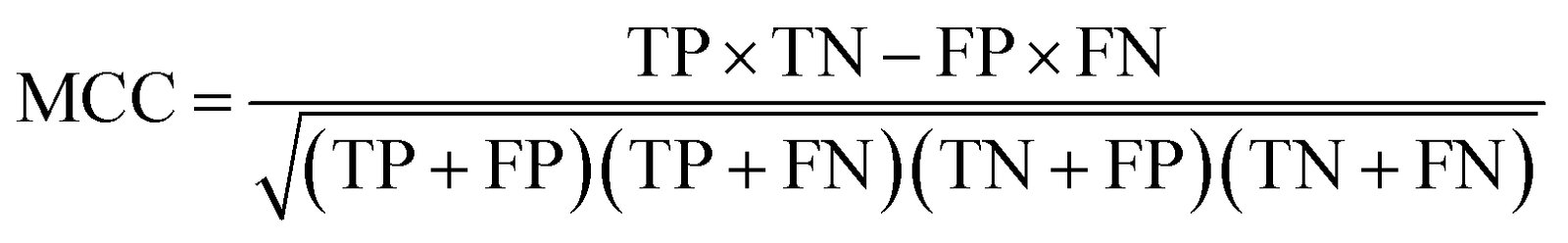 (4)	
Accuracy (Acc.)	Sensitivity or recall (Sen.)
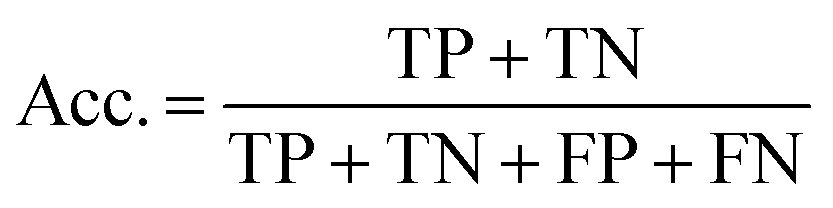 (5)	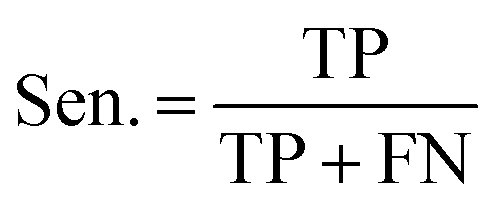 (6)
Specificity (Sp.)	Precision (Prec.)
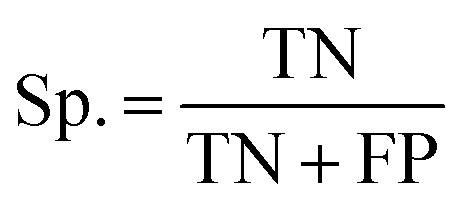 (7)	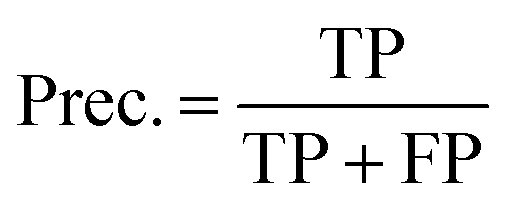 (8)
where, TP are true positives, TN are true negatives, FP are false positives and FN are false negatives.	

The above mentioned metrics are also calculated for the validation set compounds for all models during hyperparameters tuning to assess model fit. Additionally, for the GCNN model the ROC-AUC is calculated during the hyperparameters optimization phase.

To further assess the generalization and predictive performance of our modelling scheme, an additional external validation step is conducting using the benchmark dataset from Hansen *et al.*^28^ as blind set and the models performance is quantified using the abovementioned statistics. Finally, the Y-randomization test is performed in the ML workflows to assess whether the algorithms learn meaningful patterns between the features and the endpoint, and eliminate the possibility of chance correlation.^101^

### Applicability domain

To assess the reliability of the predictions of the two read-across (guided-kNN) and QSAR models, two different applicability domain (AD) methodologies are employed aiming to define the models' limitations based on the training compounds. We consider that the AD of the two models is well-defined covering the physicochemical and structural domain and thus, can cover the reliability of the predictions of the DL model as well, considering that the same training compounds are used in all three methodologies.

In the guided-kNN methodology a similarity based AD^104^ is established based on the work of Wang *et al.*^105,106^ In this case whether a query compound is located inside or outside the model's AD limits is decided by comparing its similarity to the training compounds. If this similarity is equal or larger than a predefined *S*_cutoff_ value with at least *N*_min_ training compounds, then the query compound is considered inside the AD. This is expressed in eqn (9).

9 AD_fingerprint_{*N*_min_, *S*_cutoff_}

The *N*_min_ value is selected to be equal to the *k*_opt_ value of the guided-kNN, to ensure that the training neighbours contributing to the prediction are sufficiently similar to the query compound, thus aligning the AD with the guided-kNN's local prediction mechanism. The *S*_cutoff_ value is selected as the 75th percentile of the distribution of Tanimoto similarities calculated using count-based Morgan fingerprints within the training compounds. This means that only the top 25% of the most similar training compounds are considered. This value represents a rather strict threshold, but it ensures that only highly similar compounds are included within the AD. Nonetheless, we should keep in mind that the domain boundaries may vary depending on the desired trade-off between the models' extent of use and the reliability of the predictions, thus alternative *N*_min_ and *S*_cutoff_ values can be considered.

For the QSAR model, where descriptors are used for compound featurization, a distance-based AD approach is employed. The distance of each test compound to each nearest neighbour of the training compounds is compared to a predefined threshold (eqn (10)); if this distance is lower than the threshold then it falls within the AD of the model, and its mutagenicity prediction can be considered reliable.

10 AD_descriptors_ = 〈*d*〉 + *Zσ*

To define the AD threshold of eqn (10), Euclidean distances between all training compounds based on the selected descriptors are calculated, as well as the average value of these distances (*D*_av_). In a next step the new average value 〈*d*〉 and standard deviation *σ* of the distances included in the subset of training compounds, which have lower distance than *D*_av_, are calculated. *Z*, is an empirical cut-off value, the default value is equal to 0.5.^107^

To increase the confidence in the consensus predictions, the outcome of the two AD methods (whether a prediction lies within the respective AD limits) is combined and an overall decision on the reliability of the predictions is made.^101^ The degree of reliability for each prediction is characterized as “low” (the query compound falls outside both AD limits), “moderate” (only one of the two AD limits is met), and “high” (the query compound falls within both AD limits).

## Results

In the guided-kNN workflow, after compound pre-processing (sanitization, standardization, *etc.*), 2D and 3D descriptors were calculated for the curated training, validation, and test compounds using RDKit. Compounds for which no conformers were found, and thus 3D descriptors could not be computed, were removed from the dataset, resulting in a training set of 3723 compounds and a test set of 1088 compounds. Next, descriptor selection was performed in two steps: first, mutual information filtering was applied, followed by *z*-score normalization and feature selection using the KBest method, leading to a reduced set of 50 descriptors (see Table S1). After the above filtering steps, the training set was reviewed for potential duplicate entries that might have resulted from descriptor filtering, as repeated instances could introduce bias into the models.

The remaining descriptors were then used to compute Euclidean distances between training-validation and training-test compounds. Additionally, pre-processed compounds were featurized using count-based Morgan fingerprints, and Tanimoto similarities were calculated between training-validation and training-test compounds. As per the guided-kNN methodology, for each query compound, the *k* most similar (in terms of Tanimoto similarity) training compounds were selected, while Euclidean distances were used as weighting factors for the weighted majority vote of the neighbours within the kNN scheme.

Following the grid search procedure (see Fig. S3), the *k* value in the search range that maximized the observed MCC value of the validation set predictions was found equal to *k*_opt_ = 9 neighbours. Table 5 summarizes the LOO CV accuracy results and the accuracy achieved on the validation and test sets using the optimal value. Subsequently, the guided-kNN workflow, configured with *k*_opt_ = 9, was applied to the test set compounds to evaluate its predictive performance on external data.

**Table 5:** Accuracy statistics of the guided-kNN model applied to the validation and test sets, along with the results of the LOO-CV applied to the training set, for the *k*_opt_ value

Statistics	Training set (LOO)	Validation set	Test set
MCC	0.569	0.602	0.599
Accuracy	0.788	0.807	0.802
Sensitivity/recall	0.811	0.828	0.845
Specificity	0.758	0.777	0.749
Precision	0.811	0.843	0.806

Finally, the results of a Y-randomization test, presented in Table S2, confirm the model's robustness. Models trained with shuffled endpoint values, when applied on the test set, presented poor predictive performance; both accuracy and MCC values were approximately 0.5 and 0, respectively, indicating that the actual model's performance is not driven by chance correlations. The *S*_cutoff_ value (eqn (9)) was selected as the 75th percentile (0.123) of the distribution of Tanimoto similarities within the training compounds and based on this approach 98.8% of the test set compounds fell inside the AD_fingerprint_ limits (Fig. S2).

The GCNN model was developed in two phases with the pre-processed molecular datasets of the training and validation subsets, which were featurized using ConvMolFeaturizer. In the initial phase, extensive hyperparameter search was carried out by systematically varying the parameters listed in Table 2 across five independent iterations. The best-performing hyperparameter configurations in terms of validation ROC-AUC scores were recorded and the results of this exploratory phase are summarized in Table 6.

**Table 6:** Optimized hyperparameters of phase 1 of the GCNN model development workflow

Hyperparameters	Optimized hyperparameters – phase 1				
	#1	#2	#3	#4	#5
Graph convolution layers	[64, 64]	[64, 64, 64]	[128, 64, 32]	[64, 64]	[64, 64, 64]
Dense layer size	256	128	128	64	64
Batch size	16	32	32	32	16
Dropout	9.28 × 10^−2^	6.35 × 10^−2^	7.00 × 10^−2^	1.27 × 10^−1^	3.78 × 10^−2^
Learning rate	5.21 × 10^−4^	3.36 × 10^−3^	6.82 × 10^−4^	2.07 × 10^−2^	2.96 × 10^−3^
Weight decay	10^−3^	10^−3^	10^−4^	10^−5^	10^−5^
Activation function	ReLU	ReLU	ReLU	ReLU	ReLU
Validation ROC-AUC score	0.886	0.884	0.896	0.893	0.888

The hyperparameters that were consistently found in the top-performing configurations across the five iterations were kept fixed, while the remaining hyperparameters were optimized again, as presented in Table 7, using a narrower search space. The final set of hyperparameters, including both the refined and the fixed parameters, was then used to train the optimized GCNN model.

**Table 7:** Hyperparameters optimization search space for the GCNN model after phase 1 and optimized hyperparameters of phase 2 of the GCNN model development workflow

Hyperparameters	Search space (phase 2)	Optimized hyperparameters – phase 2
Graph convolution layers	[64, 64] or [64, 64, 64]	[64, 64]
Dense layer size	64 or 128	128
Batch size	32 (fixed)	32
Dropout	*X* ∼ *U*(0.04, 0.15), step = 0.001	0.123
Learning rate	*X* ∼ *U*(0.001, 0.02), step = 0.001	0.006
Weight decay	10^−5^ or 10^−3^	10^−3^
Activation function	ReLU (fixed)	ReLU
Validation ROC-AUC score		0.896

The validation performance with the optimized hyperparameters is presented in Table 8. To assess its generalization capability, the trained GCNN final model was applied to the initial test set compounds.

**Table 8:** Accuracy statistics of the fine-tuned GCNN model applied to the validation and test sets

Statistics	Validation set	Test set
MCC	0.656	0.613
Accuracy	0.831	0.808
Sensitivity/recall	0.841	0.815
Specificity	0.817	0.799
Precision	0.860	0.831

The last step in the model validation was the Y-randomization test (see Table S3). Both accuracy and MCC values of the shuffled-endpoint models correspond to predictions made by chance; thus, the original model is robust and can be accepted.

In the QSAR modelling workflow, 777 Mold2 descriptors were generated for the curated training, validation, and test molecules using the EnalosMold2 KNIME node. After low variance data filtering, the training set comprised of 412 Mold2 descriptors which were combined with the 11 3D RDKit descriptors. Descriptors were standardized and the ones with at least one distinct value that the percentage of their instances were above an 80% threshold were removed, leading to a refined set of 359 descriptors. Next, best first feature selection was performed, and 26 descriptors were selected as described in Table S4. As before, the training compound for which conformers were not found and 3D descriptors were not generated, was excluded from modelling. The three ML models were later built in Isalos using 5-fold CV and the hyperparameters were iteratively optimized as presented in Table S5 using the Isalos AutoML function. Next, the three fine-tuned models were re-trained using the entire training set and then applied on the (previously standardized) validation set, and the accuracy was calculated as presented in Table 9. Considering accuracy values of the application of the different ML models on the validation set, the XGBoost model was selected as the final QSAR model. In brief, the XGBoost open-source library supports ensemble machine learning methods based on decision tree models. In an iterative procedure, trees are added to the ensemble so as to reduce the prediction error (loss) of previous models (in our case the objective function was the minimization of softmax probability loss).^102^

**Table 9:** Accuracy values of the three tuned QSAR models applied to the validation sets, along with the results of the five fold-CV applied to the training set

ML methodology	Training set (5-fold CV)	Validation set
J48	0.723	0.741
XGBoost	**0.795**	**0.770**
kNN	0.716	0.732

The XGBoost was later applied to the test set to assess its performance on unseen data and the respective accuracy metrics are presented in Table 10. The results of the Y-randomization test (accuracy and MCC values approx. equal to 0.5 and 0, respectively), presented in Table S6, confirm that the model is robust and not due to chance correlation. The AD_descriptors_ threshold (eqn (10)) was calculated, based on the training set, equal to 5.421 and based on this approach 99.4% of the test set compounds fell inside the AD limits.

**Table 10:** Accuracy statistics of the optimized XGBoost model applied to the test set

	Test set
MCC	0.607
Accuracy	0.805
Sensitivity/recall	0.817
Specificity	0.791
Precision	0.828

In Table 11 the accuracy statistics of the guided-kNN, GCNN, and XGBoost optimized models when applied on the external test set are presented. For fair comparisons against the guided-kNN and QSAR methodologies, the evaluation metrics for the GCNN model are calculated based on the reduced test set predicted classes comprising of 1088 compounds. The models demonstrate strong generalization to unseen data, as evidenced by the close alignment of validation and test metrics. Furthermore, since both validation and test metrics are consistent and comparable, we can conclude that there is no indication of overfitting.

**Table 11:** Accuracy statistics on the test set for the three modelling approaches and the consensus model

	Guided-kNN	GCNN	XGBoost	Consensus
MCC	0.599	0.610	0.607	0.648
Accuracy	0.802	0.807	0.805	0.826
Sensitivity/recall	0.845	0.824	0.817	0.855
Specificity	0.749	0.786	0.791	0.791
Precision	0.806	0.826	0.828	0.834

**Fig. 4:**
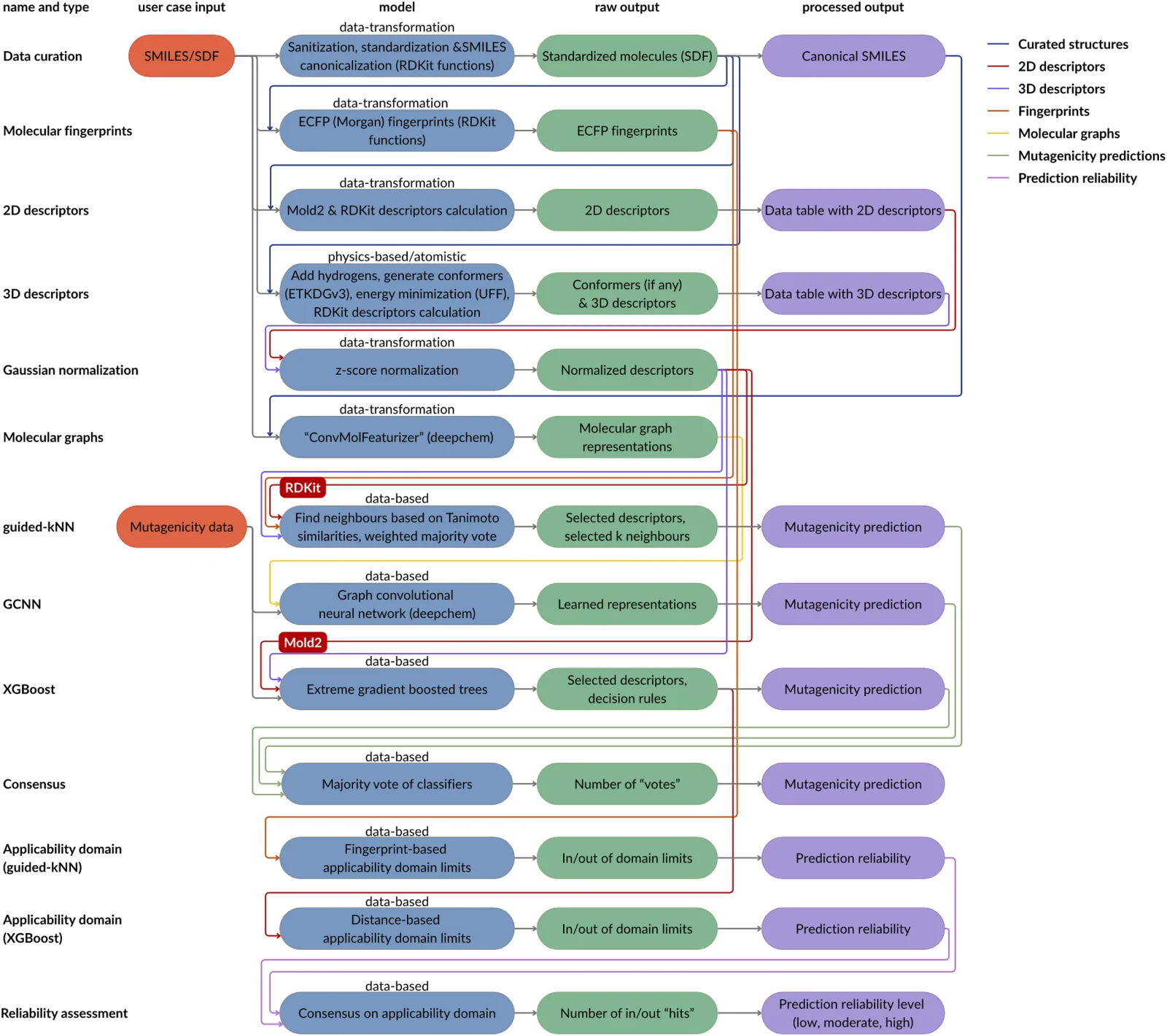
MODA consecutive pipeline presenting data prep-processing, molecular featurization, model development, consensus predictions and reliability assessment.

The three trained models were also combined in a synergistic approach, where their majority vote was considered for the final prediction. The consensus model's performance on the test set, as indicated in Table 11, is increased and outperforms any individual model in all statistic metrics. This improvement is an indication of the benefits of ensemble learning. The integration of multiple featurization approaches and modelling techniques targets complementary aspects of molecular properties, enhancing the “wisdom of the crowds” concept. This not only enhances predictive accuracy but also strengthens stakeholder confidence in the results, as the consensus approach mitigates model-specific biases and leverages the strengths of multiple featurizations. It is also noted that the accuracy achieved by the consensus model is close to the estimated interlaboratory reproducibility of the Ames test (around 85%).^108,109^ An overview of the combination of molecular featurization and modelling is presented in Fig. 4.

To assess whether the improvement of the external validation performance of the consensus model is statistically significant, we applied McNemar's test^110^ with continuity correction to the paired predictions on the 1088 test compounds: for each individual model, a 2 × 2 contingency table of predictions concordant/discordant with the consensus was constructed, and the continuity-corrected *χ*^2^ statistic (1 degree of freedom) was evaluated. The per-comparison *p*-values, calculated using Python mcnemar function,^111^ were 0.0070 (guided-kNN), 0.0255 (XGBoost), and 0.0466 (GCNN), each below the conventional threshold of *α* = 0.05. Because three comparisons were performed, we further applied the Holm–Bonferroni correction (using multipletests function) to control the family-wise error rate: the improvement over guided-kNN remained significant (adjusted *p* = 0.021), whereas the improvements over XGBoost and GCNN were marginal after adjustment (adjusted *p* = 0.051). The smaller, non-significant gaps against XGBoost and GCNN reflect the already high accuracy of these two individual models rather than an absence of improvement. The consensus model outperformed all three individual models on the observed metrics, showing a consistent and directionally positive advantage thus, these results support the selection of the consensus as the final predictive model.

To assess the robustness of our predictive workflow, we also performed a sensitivity analysis to quantify the impact of stochasticity in the 3D conformer generation step. For this reason, an external set of compounds was selected (“log *P* dataset” from Titania tool^26,112^) comprising of 14 207 molecules. Any molecule that failed sanitization was removed from the dataset, leaving 13 844 compounds available for further analysis. Next, conformer generation was performed using ETKDGv3 and UFF optimization followed by 3D descriptor calculation and final mutagenicity prediction. This process was repeated 20 times using in each run a different random seed to initiate the conformer embedding process. The results demonstrated a high degree of stability, with the consensus model predictions varying by only approximately 0.3% across all runs. Given that the model's overall accuracy is 83%, this 0.3% variability is negligible. This finding confirms that the majority voting scheme mitigates the possible sensitivity of the guided-kNN and XGBoost models to molecules spatial “fluctuation” and thus the stochastic nature of the 3D pipeline (generation of conformers and calculation of 3D descriptors) is not a significant source of error in the final predictions.

Finally, we performed an additional external validation step using the Hansen *et al.*^28^ benchmark dataset, as blind set. In brief, we first pre-processed the dataset (comprising of 6512 compounds) by sanitizing and standardizing the structures. We also filtered out compounds already present in the 5076 compounds of the training set, to avoid resubstitution bias. After this filtering step, 2035 unique compounds remained, which were featurized and used as input into the guided-kNN, GCNN, and XGBoost models before being integrated into the consensus scheme. It is noted that for nine compounds where no conformers could be generated, and thus 3D descriptors could not be calculated, the consensus prediction was derived only from the GCNN model. The prediction metrics from our consensus scheme yielded an MCC of 0.61 and an accuracy of 0.81.

### Model and dataset FAIRification

The three developed models have been partly FAIRified as per Wilkinson *et al.*’s^66^ recommendations for the application of FAIR principles to computational workflows. They have been deployed *via* a web-service, assigned a unique URI through the Enalos Cloud Platform (https://www.enaloscloud.novamechanics.com/insight/polis/) and supplemented with an API to enhance their interoperability (https://www.enaloscloud.novamechanics.com/insight/swagger-ui/). To enhance models' transparency and support their reproducibility and regulatory compliance, the metadata of the entire predictive workflow has been reported following the MODA standardized template (see SI files) *via* the Easy-MODA tool.^67^ The MODA guidelines ensure that each step of this complex workflow is reported in a consecutive manner and visualized in a pipeline (Fig. 4). In our case, the MODA pipeline was colour-coded to facilitate readability and comprehension of the data flow. For the sake of brevity, Mold2 and RDKit 2D descriptors were summarized within the same step and variable selection was reported together with modelling. The final report was processed offline to ensure that tables and equations were properly formatted.

As diverse computational fields are combined to study new materials, drugs and chemicals (*e.g.*, through Integrated Approaches to Testing and Assessment-IATAs), there is a growing need to expand standardized templates that support transparent documentation of modelling metadata. Within the Easy-MODA tool an effort to align the QSAR model reporting format (QMRF) with the MODA fields is made.^67^ Nonetheless, directly incorporating the QMRF report into MODA for QSAR-type models may be a more suitable approach, as the QMRF reporting standard is specifically developed and refined to capture the full metadata required for QSAR models. The wider adoption of such standardized templates, in combination with a consensus among modellers and regulators can reveal similar limitations within MODA leading to updated versions of these guidelines. While this will inevitably involve a trade-off between time and complexity in the documentation process, it will also promote FAIR principles and reduce reliance on black box modelling practices.

In line with the FAIR data principles,^113^ the curated and enriched with 2D and 3D molecular descriptors dataset used for model development and validation has been uploaded in the ChemPharos database (https://db.chempharos.eu/datasets/Datasets.zul?datasetID=ds18). For each compound, additional metadata (CID, IUPAC names, InChIKey, PubChem link) are appended whenever available, extracted from the PubChem database based on the compounds canonical SMILES using the Enalos + KNIME nodes.^72^ The data are in tabular format to promote their future reuse in additional cheminformatics analysis and the compounds of the training, validation and test sets are precisely annotated to facilitate guided-kNN and XGBoost modelling reproducibility. For the replication of the GCNN model, stakeholders can directly download the dataset from the TDC database^63^ and use the same splitting protocol.

### Interpretation of the descriptor space

In addition to the development of robust models that accurately predict mutagenicity, special emphasis was also placed on explaining the mechanisms that drive the mutagenicity by interpreting the selected descriptors involved in guided-kNN and XGBoost models. After all, the mechanistic interpretation is one of the principles of the Organization for Economic Cooperation and Development (OECD) for the regulatory assessment of QSAR models.^114^ In this effort, we conducted a literature review of the selected descriptors and employed explainable AI (XAI) techniques, performing Shapley additive explanations (SHAP) analysis.

**Fig. 5:**
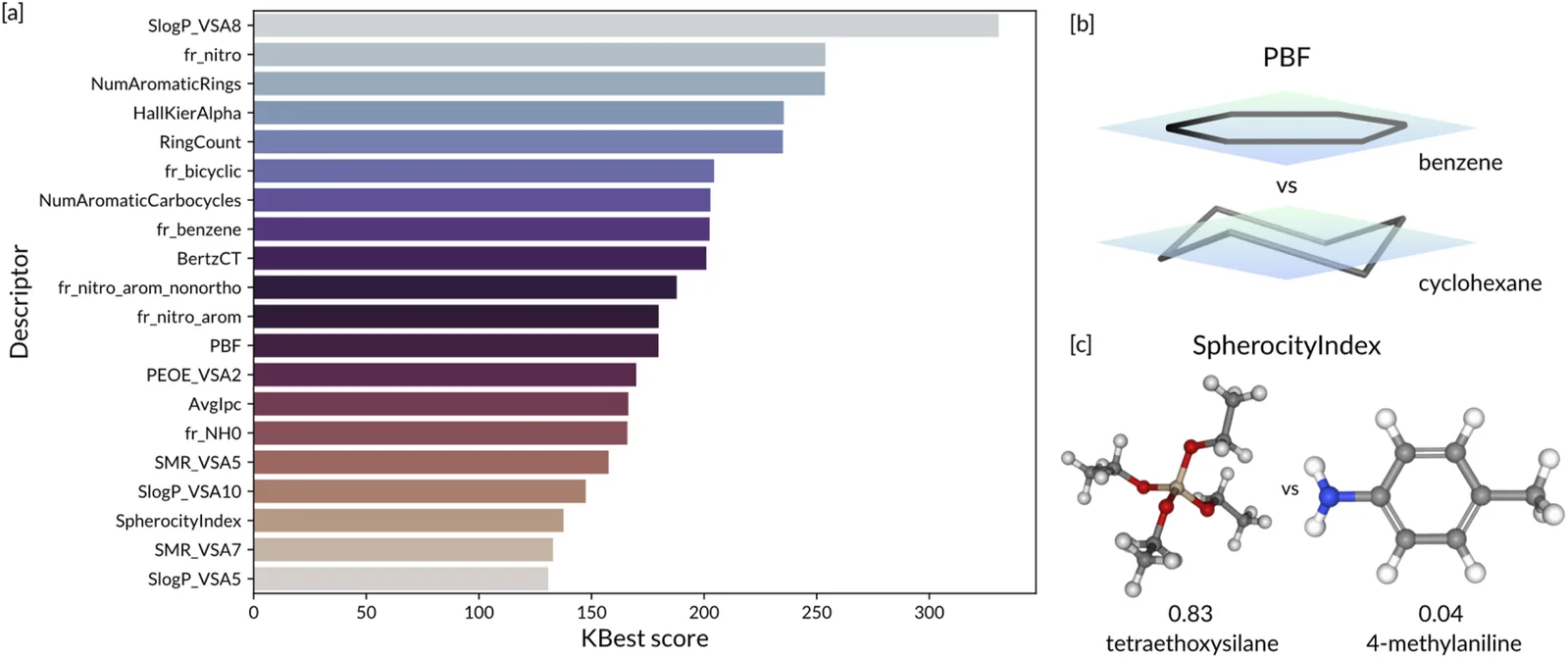
(a) Top 20 selected 2D and 3D descriptors used in the guided-kNN model. The full list of descriptors can be found in Table S1. (b) Example of PBF (blue plane) for a benzene ring and a cyclohexane molecule in a chair conformation. Image based on the depiction of Firth *et al.*^116^ and Hamilton *et al.*^117^ (c) Example of SpherocityIndex values for tetraethoxysilane (quasi-spherical molecule) and 4-methylaniline (planar molecule).

The selected 2D and 3D RDKit descriptors used within the guided-kNN scheme for the mutagenicity prediction are ranked based on their KBest score in Table S1. Among the top-ranked RDKit descriptors (see Fig. 5a), aromaticity-related structural features (number of aromatic rings, aromatic carbocycles, benzene rings) and nitro group-related features (number of nitro groups) are identified as key descriptors. Both aromatic (especially polycyclic aromatic compounds) and nitroaromatic compounds have been associated with mutagenic activity.^109^ The planar structure of aromatic rings can intercalate between DNA base pairs, causing structural distortions that can lead to mutations whereas, the nitro group can undergo metabolic activation to form reactive intermediates that interact with DNA.^109^ It should be also noted that the nitro aromatic group substituents may also act as detoxifying substructures of nitroaromatics depending on their position.^109^ For instance, the introduction of bulky alkyl substituents ortho to the nitro functional group in 4-nitrobiphenyl eliminates mutagenicity compared to the parent compound. This reduction in mutagenic potential is associated with steric hindrance, which forces the nitro group out of the aromatic plane and disrupts its reactivity, that could otherwise lead to DNA adducts formation. These findings may be generalizable to other nitroaromatic compounds with similar substitution patterns,^115^ nonetheless further assessment of the different substituent effects could be addressed in future studies. In our model, the number of *ortho* nitro aromatic group substituents can be derived by the two selected descriptors (number of nitro benzene ring substituents and of non-*ortho* nitro benzene ring substituents), thus overall, the guided-kNN model captures competing effects as it accounts for structural features that both promote and mitigate mutagenicity.

Other physicochemical features such as hydrophobicity-based MOE descriptors (S log *P*_VSA8, S log *P*_VSA10, S log *P*_VSA5) suggested that hydrophobicity (often denoted as log *P* and commonly derived as a key descriptor for mutagenicity prediction^118^) influences cellular uptake, as it controls the penetration of molecules to cell membranes and interacts with intracellular components, increasing the potential of mutagenic interactions. Molar refractivity-based descriptors (SMR_VSA5, SMR_VSA7) encode molecular polarizability, and can serve as a measure of the interactions between biological receptors and substrates.^54^ Other highly ranked descriptors, such as spherocity, which measures molecular anisotropy, are not commonly associated with mutagenicity in the literature. Nonetheless, descriptors like Plane of Best Fit (PBF, Fig. 5b) and SpherocityIndex (Fig. 5c) effectively capture molecular shape and planarity (key structural features of known mutagenic polycyclic aromatic scaffolds such as acridine, phenanthrene, pyrene, and quinoxaline).^119^ In brief, spherocity values close to zero correspond to flat molecules, whereas values near one describe spherical ones.^54^ Finally, low PBF values suggest molecules are nearly planar, whereas higher PBF values reflect greater three-dimensionality, with atoms more dispersed out of a single plane.^116,117^

For the 2D Mold2 and 3D RDKit descriptors used in the development of the XGBoost model, SHAP analysis was possible since XGBoost generates probability outputs. SHAP, a feature-based interpretability method, is based on the concept of Shapley values (*φ*) originating from game theory. In this course, the Shapley value (payoff, fair share) *φ*_*i*_ of player *i* (eqn (11)), is defined based on the individual contribution of this player to the collaborative game.^120,121^11
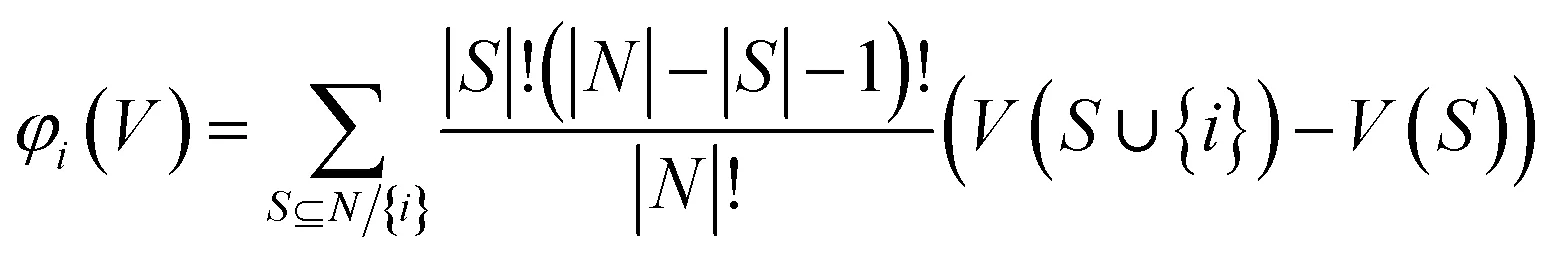
where *S* denotes a coalition, *N* is the total number of players, *V*(*S*) is the value of the game for coalition *S*, (*V*(*S*∪{*i*}) − *V*(*S*)) is the marginal contribution of player *i* to the coalition *S*, 
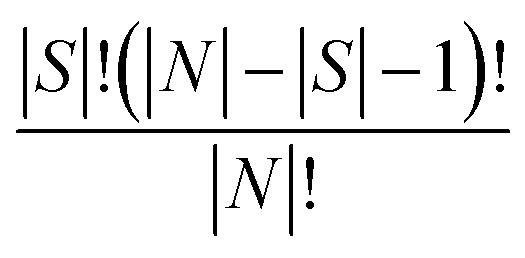
 is the weight of the marginal contribution and, 
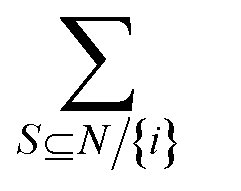
 spans all possible coalitions excluding player *i*.

In ML, SHAP values can be used to quantify the contribution of each variable to the model's outcome. The players correspond to features, the coalitions to feature subsets, and the value of the game to the model's prediction. SHAP values are computed by averaging the marginal contributions of each feature across all possible subsets, ensuring that feature importance is assessed in a fair and comprehensive manner. The magnitude of a feature's contribution is correlated with its mean absolute SHAP value, allowing to rank features based on their impact on model predictions.^121^

**Fig. 6:**
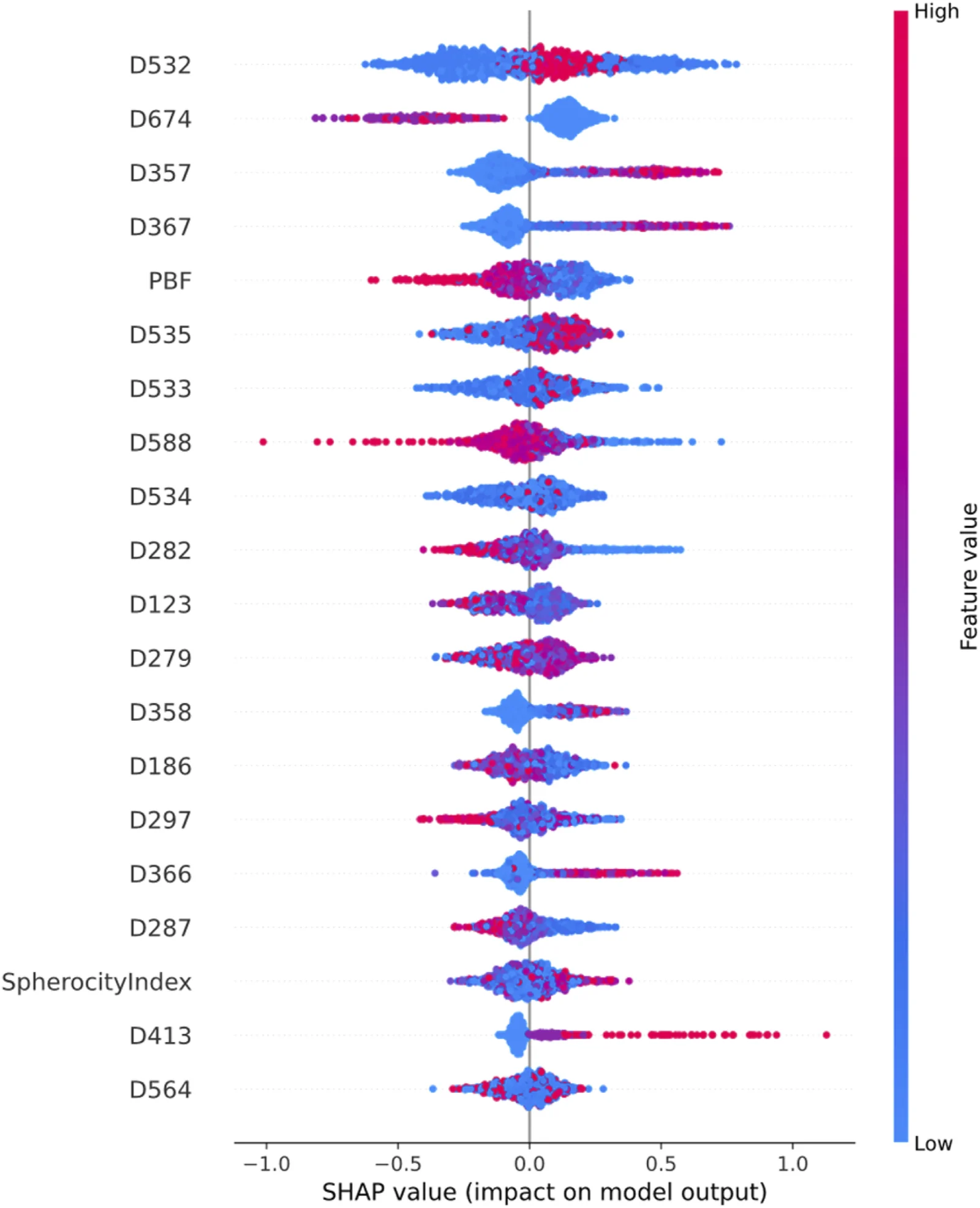
SHAP analysis summary plot using the XGBoost model for the test samples.

Considering this, a SHAP analysis was performed in Python using Tree SHAP to evaluate the influence of each selected descriptor in the XGBoost model's mutagenicity predictions. The global SHAP analysis, shown in the beeswarm plot of Fig. 6 illustrates the magnitude and direction of each descriptor's impact on the model's output (see Table S4 for descriptor definitions). Features with larger mean absolute SHAP values were identified as the most influential ones. Each point in the summary plot represents a sample from the test set, with its colour encoding the descriptor's value.^121^ Descriptors are ordered by importance, where positive SHAP values indicate an increase of the likelihood of mutagenicity, whereas negative values are associated with non-mutagenicity (in terms of the Ames test). Apart from global feature importance, SHAP values also enable local interpretability, explaining how individual descriptor values influence predictions for a specific sample. This aspect allows for a refined understanding of the model's decision-making process.

The SHAP analysis highlighted mainly topological and topochemical properties.^54^ To begin with, Burden matrix eigenvalues (D532, D533, D535, D534, D564, D588) and information content indices (D279, D282, D297) emerged as critical factors, all of which quantify molecular topology, accounting for atomic contributions and molecular diversity of atoms and bonds, respectively.^54,55^ Although these descriptors do not directly indicate known mutagenic substructures, they suggest that the overall electron distribution and molecular connectivity are important for how a compound is metabolized and how it may form reactive intermediates, ultimately influencing mutagenic potential. Additionally, multiple path indices (D357, D358) were important, indicating that the spatial arrangement of topological features also contribute to mutagenicity predictions.^54^ Meanwhile, the SpherocityIndex and PBF were found among the highly contributing descriptors: for instance, SHAP analysis indicates that lower PBF values (blue dots), corresponding to more planar compounds, are associated with increased mutagenic potential, which is consistent with literature indicating planarity of molecules, as a possible factor for DNA intercalation, especially in polycyclic aromatic systems.^109^

Another top-ranked descriptor (D367) was the sum of topological distance between the vertices of nitrogen (N) and phosphorus (P) atoms, with higher values (*e.g.*, in cases of structurally distant N and P atoms in different branches or regions, in cases of elongated molecules or in cases where the N and P atoms are sparse) contributing to mutagenicity. Lower D367 values indicate direct P–N bonds within the molecule or that these atoms are possibly clustered within the same functional region. Both N and P-containing compounds are considered important therapeutic agents in the medicinal field due to their bioavailability, selectivity and biocompatibility.^122,123^ Among the top-ranked descriptors was the number of total hydroxyl groups (D674), which can influence a compound's solubility,^124^ metabolic pathways, and overall biological reactivity, and as a result they can potentially alter mutagenicity risk.^125^ Finally, the negative SHAP values associated with the compounds average molecular weight (D123) indicate that, within the XGBoost model, molecules with lower average molecular weight tend to be predicted as more mutagenic. Heavy atoms such as chlorine and iodine may be associated with toxic structural alerts.^126^ Thus, it is important to interpret these findings in combination with other descriptors (those capturing shape, connectivity and complexity) which also modulate a compound's mutagenic potential.

Even if the KBest method (explores descriptors' individual correlation to mutagenicity) and the SHAP analysis (quantifies selected descriptors' contributions to mutagenicity predictions) are different approaches and are based on different descriptors (except from the 3D RDKit descriptors), their results regarding the descriptors space assessment are largely complementary rather than contradictory. The KBest method used in the guided-kNN prediction prioritized classical structural alerts, such as nitro-aromatic groups and ring-based descriptors, which are well-established indicators of DNA intercalation and reactive metabolite formation. On the other hand, the XGBoost/SHAP analysis highlighted topological and electronic features that capture molecular connectivity and electronic distribution variations. Despite their methodological differences, both strategies converged on the importance of molecular shape, as evidenced by the common emphasis on planarity and sphericity through descriptors like PBF and SpherocityIndex. This convergence reinforces the conclusion that flat, extended structures are frequently associated with higher mutagenic risk. Moreover, the conceptual parallels observed between descriptors that encode complexity (BertzCT and D349, D357, D358) and topological information (AvgIpc and D279, D282, D287, D297), even though “quantified” in different manner, reinforce our choice of developing a consensus scheme for the mutagenicity prediction where different chemical representations and modelling approaches are combined, supplementing each other and supporting user confidence in the results.

**Fig. 7:**
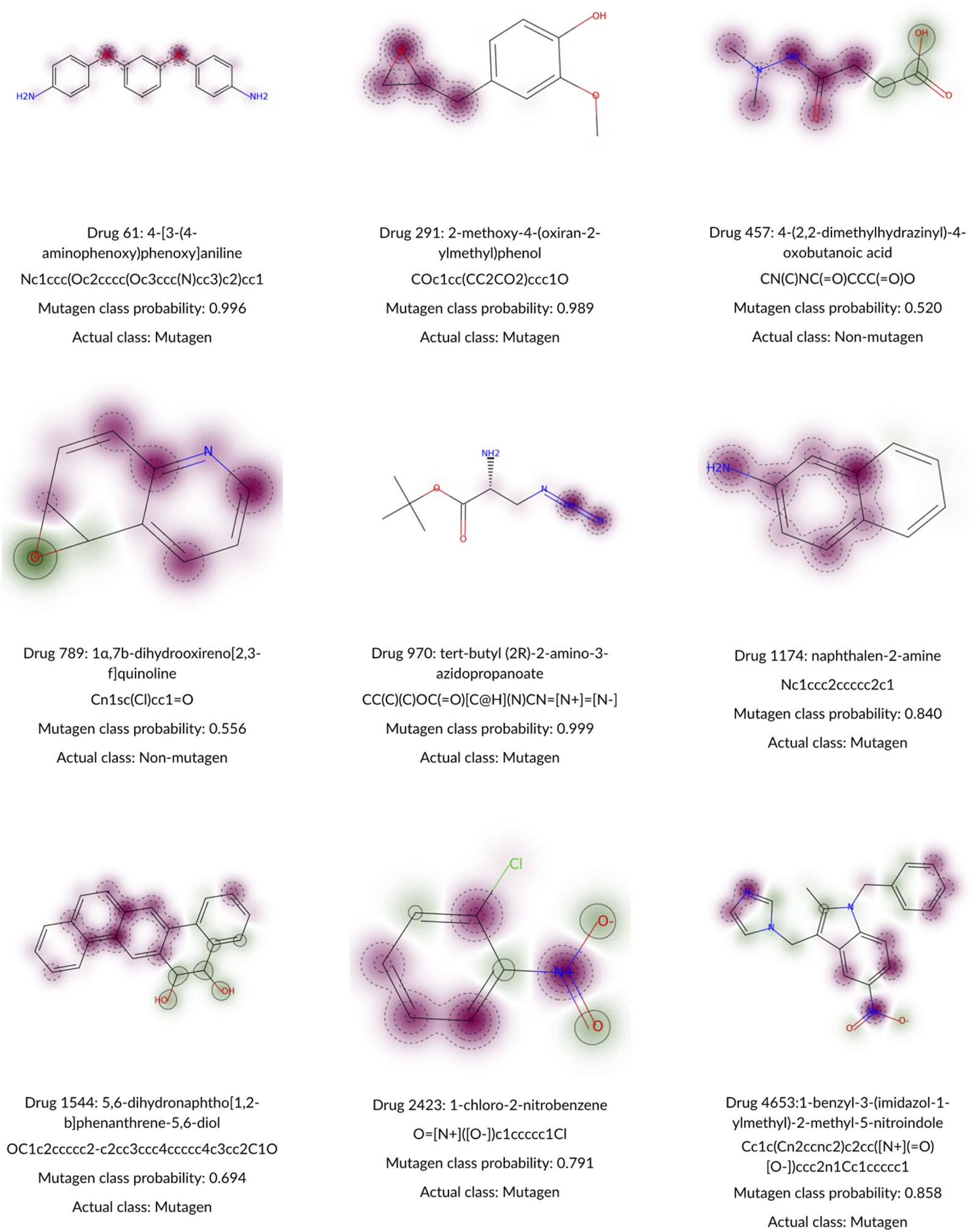
Atomic contribution maps for 9 randomly selected test set compounds predicted to be mutagenic. Pro-mutagenic (increasing mutagenicity) and anti-mutagenic (decreasing mutagenicity) atoms are highlighted in pink and green colours, respectively. IUPAC names are retrieved from the PubChem database based on the compound SMILES identifiers.

The use of the GCNN model allows to calculate and visualize the contributions of heavy atoms within the molecule to the prediction of mutagenicity, enhancing in this manner the model's interpretability, by offering another tool for assessing the mutagenicity mechanism. In brief, the atomic contribution is calculated as the predicted probability difference between the whole molecule, and the remaining part of the molecule after atom removal.^127–129^ To better visualize atomic contributions, through a colour mapping the weights are normalized by dividing by the maximum absolute weight value. Atoms highlighted in pink colour contribute positively to the mutagenic probability (their presence enhances mutagenicity) whereas green colour indicates negative contribution to the mutagenic probability. To this end, 9 test set compounds predicted as mutagens (Ames positive) were randomly selected, and the atomic contributions maps to mutagenicity are presented in Fig. 7. Pro-mutagenic atoms are mainly located in aromatic rings, which is consistent with the descriptor analysis. We acknowledge that a larger sample of mutagenic compounds would likely lead to more reliable and generalizable conclusions, which could be addressed in future studies. Additional examples of atomic contribution maps can be found in Table 13 of the Case study section, where non-mutagenic (Ames negative) compounds are also included.

**Fig. 8:**
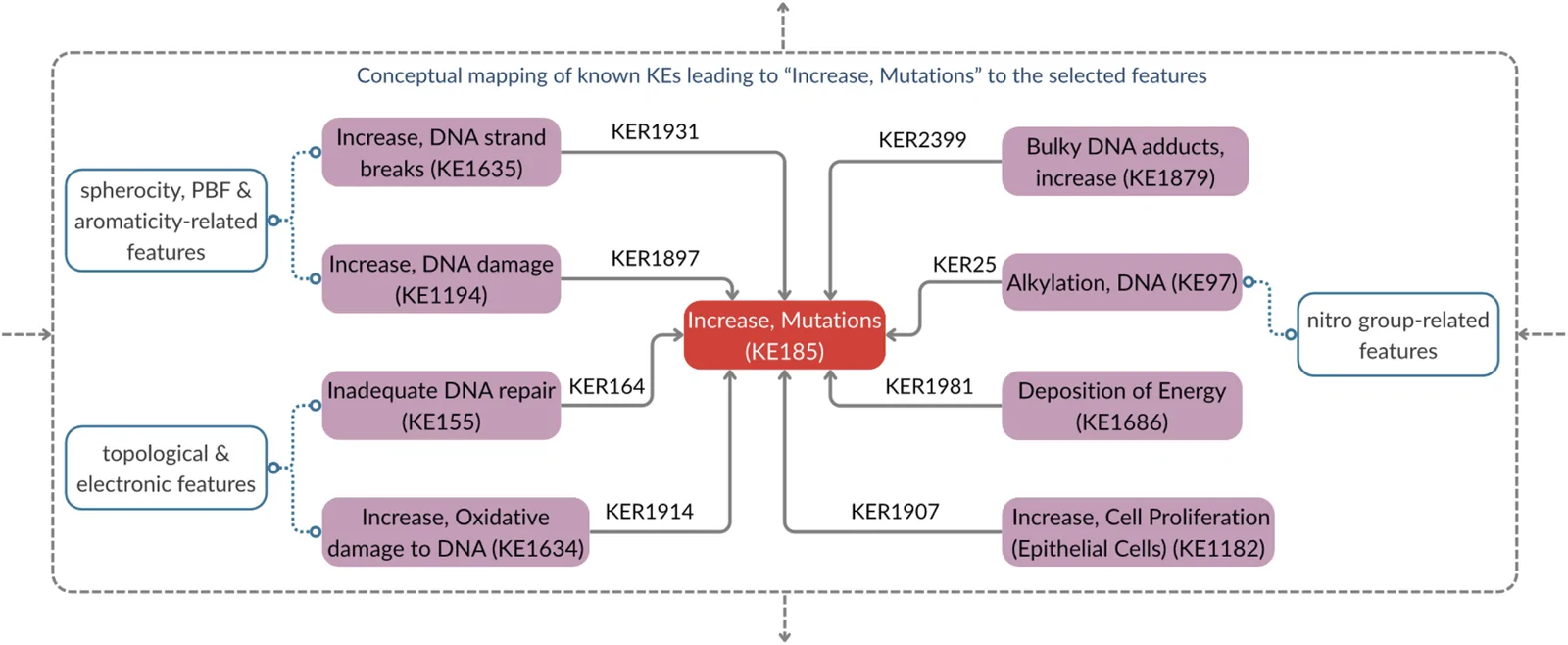
Segment of the AOP network^131^ including KE185: increase, mutations. This image depicts the immediately preceding KEs leading to increase, mutations through the causal KERs. Upstream and downstream events of the depicted KEs are shown with grey dashed arrows. The selected descriptors (in blue boxes) are mapped to specific Kes (conceptual alignment).

Finally, the abovementioned findings were mapped to Adverse Outcome Pathways (AOPs), and particularly to AOP Key Event (KE) 185: “Increase, Mutations” (https://aopwiki.org/events/185).^130^ The KE 185 was visualized as an AOP network (https://wikikaptis.lhasacloud.org/#/ke/185/viewer)^131^ and the KEs leading to “Increase, Mutations” were further examined to explore possible connections between them and the descriptors involved in our models. It should be noted that this is an effort of a conceptual alignment of the known KEs to the selected features, to enhance model interpretability and provide mechanistic insights, and should not be perceived as a weight of evidence assessment. In this course, nitro groups, pointed as informative descriptors in the KBest method, through metabolic activation, can be bioactivated into reactive intermediates capable of forming DNA adducts and provoke DNA alkylation (KE97). In parallel, the selected shape-related descriptors, highlight the role of planarity in facilitating DNA intercalation potentially leading to DNA strand breaks (KE1635) and contributing to DNA damage (KE1194). Finally, descriptors that quantify molecular complexity and electronic distribution may reflect the subtle electronic perturbations that predispose compounds to induce oxidative stress (KE1634) or interfere with DNA repair mechanisms (KE155), leading to increased mutations. These findings suggest that the selected descriptors for modelling correspond with established KEs, reinforcing their biological relevance and enhancing stakeholders' trust in the model predictions. Fig. 8 presents a segment of the AOP network^131^ involving KE185 and its preceding KEs, along with the interlinking key event relationships (KERs) and the associated selected features.

### Comparative analysis of mutagenicity models

As a next step, we evaluated our model against existing models to showcase its potential benefits. ADMETlab 3.0 is a web platform that gives free access to a collection of predictive models for physicochemical, medicinal chemistry and ADMET-related endpoints, including mutagenicity.^132^ We used ADMETlab 3.0 to predict the mutagenicity of our reduced test set of chemicals (1088 compounds) and compared the results with our consensus model. The canonical SMILES of the test set were provided as input to the tool. The prediction metrics from ADMETlab 3.0 yielded an MCC of 0.668 demonstrating higher overall classification performance than our consensus model and an accuracy of 0.824. However, while the tool exhibited high sensitivity (0.982) in the prediction of mutagenic compounds, its specificity was considerably lower (0.628) for the Ames negative ones, whereas our consensus model offered a more balanced sensitivity/specificity trade-off. The two models suit different use cases, and stakeholders may choose between them depending on whether their goal is the exclusion of potential mutagens (Ames positive) or the reliable retention of promising candidates. ADMETlab's high sensitivity makes it more appropriate for hazard flagging applications, where the priority is to identify as many potential mutagens as possible. Our consensus model's more balanced profile is useful when false positives carry a cost, *e.g.*, when prioritizing candidates for further evaluation, where wrongly discarding promising Ames negative compounds should be avoided.

We also compared our approach to the aiQSAR model of Vukovic *et al.*^30^ which can be also considered a read-across approach as predictions are generated within “local” groups of similar compounds. To ensure consistency, we first pre-processed their dataset (5764 compounds) following the same procedures applied in our analysis, including structures' sanitization and standardization. Then, to eliminate potential bias we further filtered out compounds already present in our training set, which had been used to develop our models. After this filtering step, 2173 unique compounds remained, which were then featurized accordingly and used as input into our three trained models before being integrated into the consensus scheme. The prediction metrics from our consensus scheme yielded an MCC of 0.632 and an accuracy of 0.820, whereas the aiQSAR predictions (limited to the 2173 compounds) achieved an MCC of 0.597 and an accuracy of 0.802. These results indicate that our approach may offer improved predictive performance, particularly in terms of model robustness and reliability on a curated dataset.

### Comparative analysis of the guided-kNN approach

To evaluate the advantages of our hybrid local model over the standard kNN algorithm, we compared the guided-kNN model with established kNN classifiers. In detail, four classifiers were evaluated: the WEKA 3.7 IBk algorithm implemented *via* the KNIME IBk node,^133^ the native KNIME *k*-Nearest Neighbor implementation,^134^ the EnaloskNN KNIME node,^103^ and the Python-based KNeighborsClassifier.^135^ To ensure fair comparisons, all models were trained using a consistent dataset comprising of the 50 selected 2D and 3D RDKit descriptors for the 3723 training compounds. The number of neighbours was fixed at the optimal value *k*_opt_ = 9, and inverse Euclidean distances were used as weighting factors. In the KNeighborsClassifier the algorithm used to compute the nearest neighbours was set to “auto” (automatically selected among the BallTree, KDTree algorithms and brute-force search based on the training data). The performance metrics on the validation set are presented in Table 12. Similar results, occur when assessing the performance of the models on the test set. Notably, our guided-kNN model consistently outperformed the standard kNN classifiers.

**Table 12:** Accuracy statistics on the validation set comparing the performance of different standard kNN implementations with the guided-kNN approach

Statistics	Guided-kNN	WEKA 3.7 IBk (KNIME)	*k*-nearest neighbor (KNIME)	EnaloskNN (KNIME)	KNeighborsClassifier (Python)
MCC	0.602	0.542	0.519	0.546	0.519
Accuracy	0.807	0.780	0.769	0.781	0.769
Sensitivity/recall	0.828	0.825	0.815	0.825	0.815
Specificity	0.777	0.714	0.701	0.719	0.701
Precision	0.843	0.807	0.798	0.810	0.798

A challenge within this hybrid prediction scheme lies in the geometric consistency between the two molecular representation spaces, *i.e.*, the Tanimoto-based similarity space derived from count-based Morgan fingerprints and the Euclidean distance space based on physicochemical descriptors. To evaluate the degree of alignment between these two hyperspaces, we conducted a quantitative analysis using Spearman rank correlation (*ρ*), which evaluates whether the relative ordering of pairwise distances is preserved between the two hyperspaces through a monotonic function. All calculations were performed on the training data. To enable direct comparison, the Tanimoto similarity matrix was converted into a pseudo-distance matrix defined as dist_*T*(*i*,*j*)_ = 1 − *T*(mol_*i*_, mol_*j*_), aligning it with the scale of the Euclidean distance matrix.

Globally, the Spearman correlation between the two distance matrices was moderate (*ρ* = 0.40), indicating only partial alignment between the fingerprint- and descriptor-derived hyperspaces. However, when considering local structure (defined here as the 25 nearest neighbours of each compound based on Tanimoto similarity) the relationship was notably stronger, with an average local Spearman correlation of 0.60 ± 0.13. These findings suggest that, although the two hyperspaces differ at the global level, they preserve local neighbourhood structure.

This partial but consistent local alignment supports the rationale for the guided-kNN strategy employed here. While molecular fingerprints effectively capture structural similarity and thus guide the selection of chemically relevant neighbours, physicochemical descriptors provide fine-grained resolution within these neighbourhoods. Consequently, prediction weights derived from descriptor distances may not directly mirror the similarity-based ranking of neighbours: a molecule that is highly similar in fingerprint space may contribute less to the prediction if it is more distant in descriptor space, while a less similar neighbour may exert a greater influence due to its proximity in descriptor space. Nonetheless, these weights remain functionally consistent with the underlying chemically meaningful clustering established by the fingerprint similarity and leveraging both molecular representations likely contributes to the improved predictive performance observed compared to conventional descriptor-based kNN models.

### Case study: mutagenicity assessment of PFAS alternative compounds

The development of self-cleaning coatings for glass applications, including photovoltaic panels, is essential for their trouble-free operation, maintenance, and energy efficiency, based on the coatings' hydrophobic properties. To achieve coatings' high hydrophobicity, chemical modification with fluorosilanes (*e.g.*, perfluorooctyl-triethoxysilane (PFOTS)) and polyvinylidene fluoride is reported.^136,137^ Nonetheless, the per-and poly-fluoroalkyl substances (PFAS) substances, “fluorinated substances that contain at least one fully fluorinated methyl or methylene carbon atom (without any H/Cl/Br/I atom attached to it)”,^138,139^ also known as “forever chemicals” are persistent pollutants of the environment and have a high toxicological impact on the ecosystems and humans.^140,141^ In light of this, industry and safety authorities are working together to decrease their use in products and move towards safer and more sustainable solutions. However, before replacing PFAS with alternative compounds, it is essential to conduct an initial hazard assessment of the candidate compounds to avoid “regrettable substitutions”, that is chemicals with unknown or unforeseen adverse effects are used to replace chemicals that have been identified as problematic, finally resulting in the same or worse adverse outcome.^142^

In this course, we selected PFAS alternative compounds as a case study to predict their mutagenicity. Based on literature search,^136,137,143^ we selected 8 non-fluorinated reagents used for the development of superhydrophobic transparent films, and we extracted their SMILES representation from the PubChem database (http://pubchem.ncbi.nlm.nih.gov). Then we prepared the molecules as described before to be imported in the three models, *i.e.*, structures sanitization and standardization, featurization, descriptors' normalization and filtering. The predictions for each one of the PFAS substituents are presented in Table 13. The maps of atom contributions to mutagenicity based on the GCNN model for each one of the compounds are also presented, where pro-mutagenic and anti-mutagenic atoms are highlighted in pink and green colours, respectively. We can observe that all the query molecules are predicted as Ames negative in the consensus scheme and all fall within the AD limits of the guided-kNN and XGBoost models, except the HMDS.

**Table 13:** Consensus Ames mutagenicity predictions for a set of candidate PFAS substitutes

Reagent [CAS]	Guided-kNN	AD fingerprints	GCNN	XGBoost	AD descriptors	Consensus prediction	Reliability level
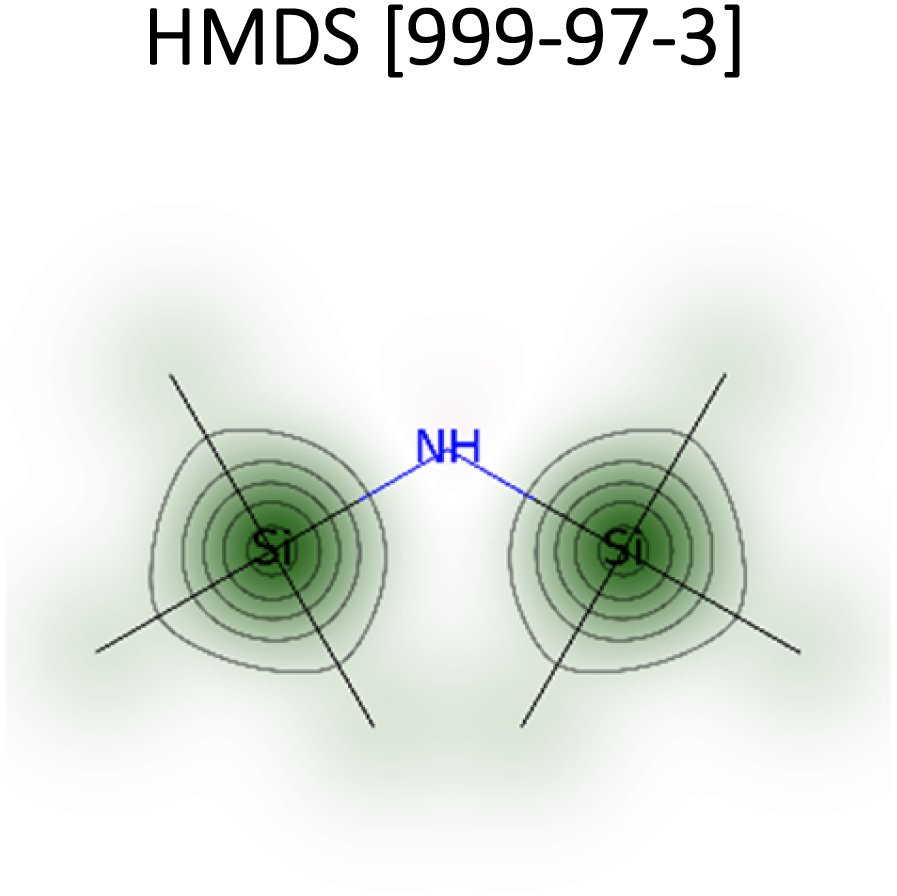	Negative	Out	Negative	Negative	In	Negative	Moderate
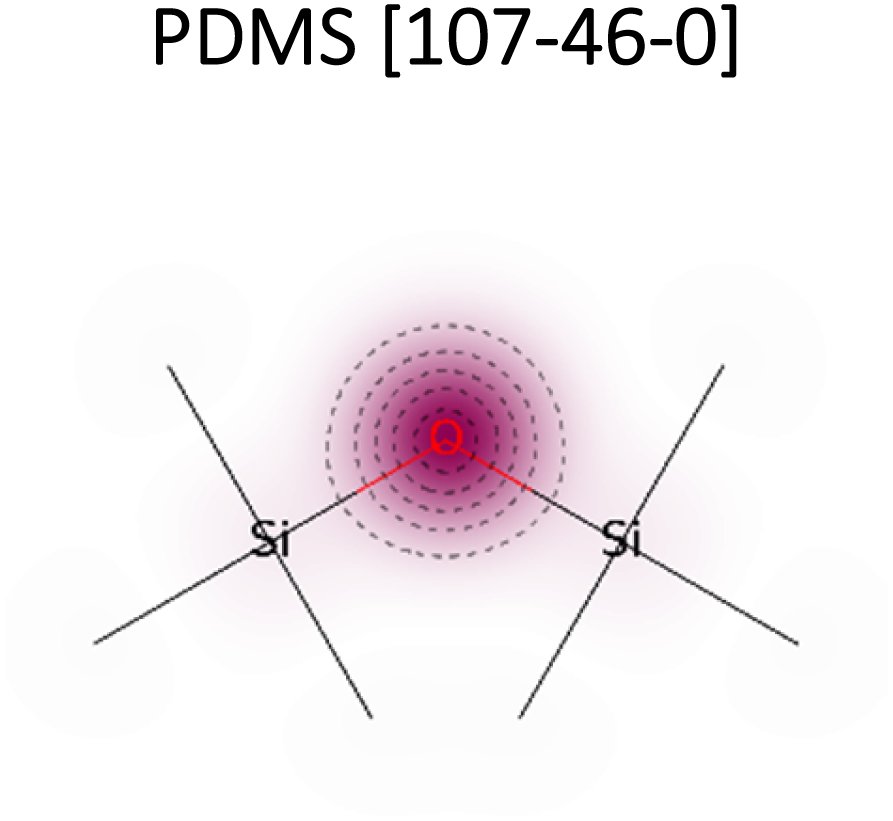	Negative	In	Positive	Negative	In	Negative	High
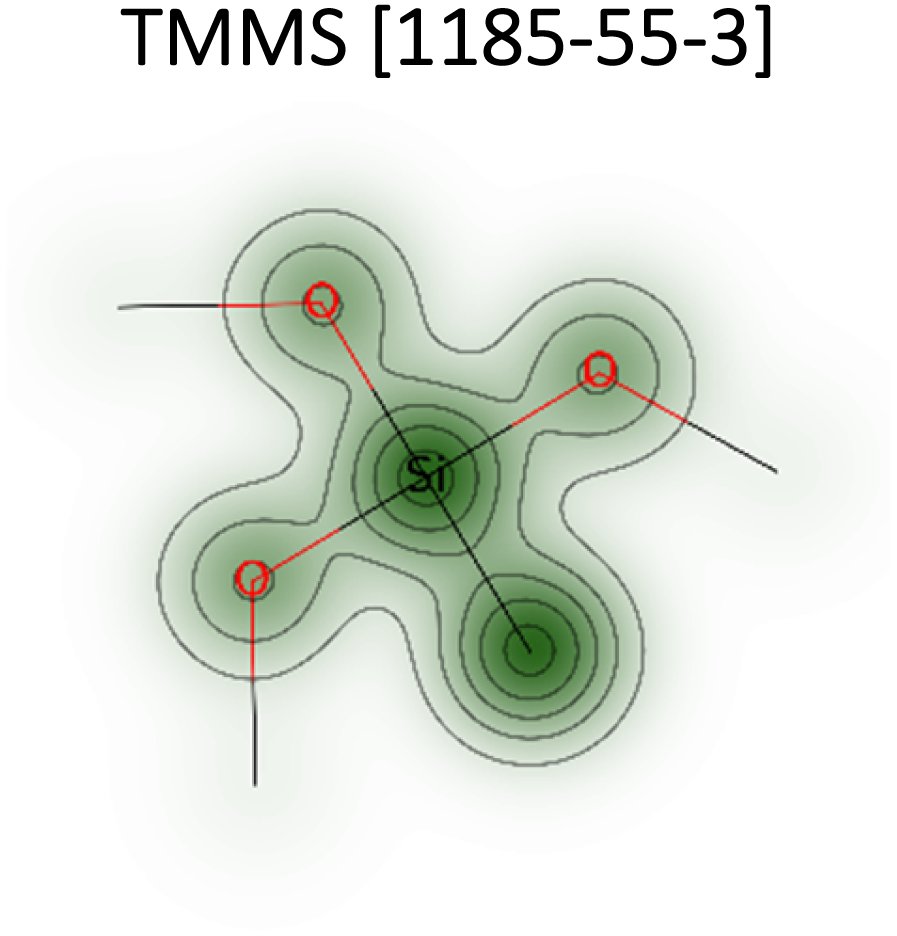	Negative	In	Negative	Negative	In	Negative	High
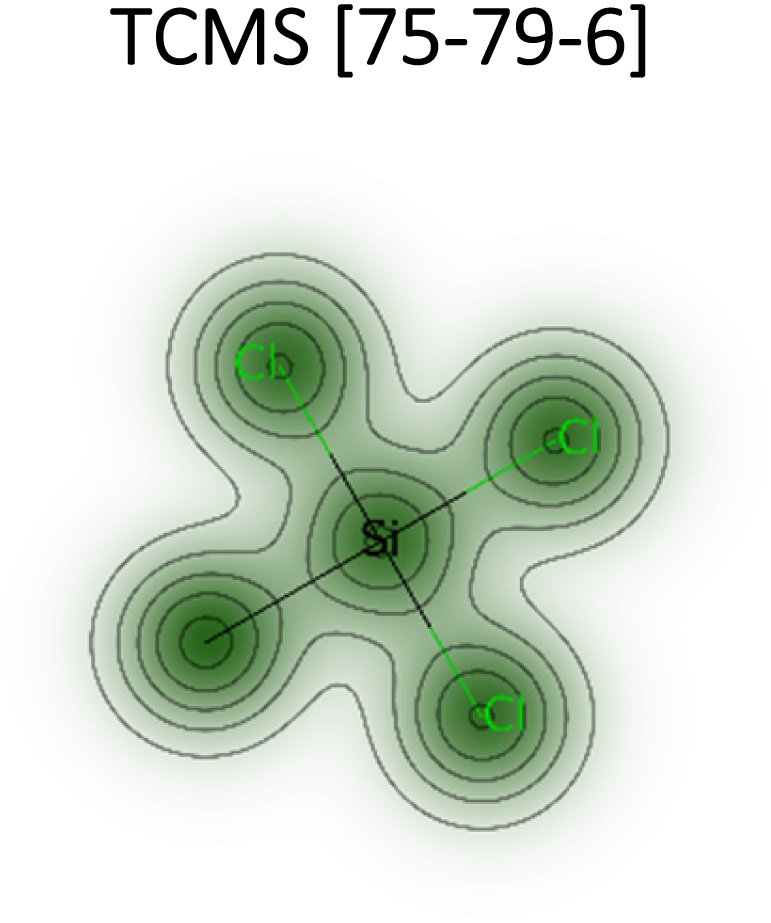	Negative	In	Negative	Negative	In	Negative	High
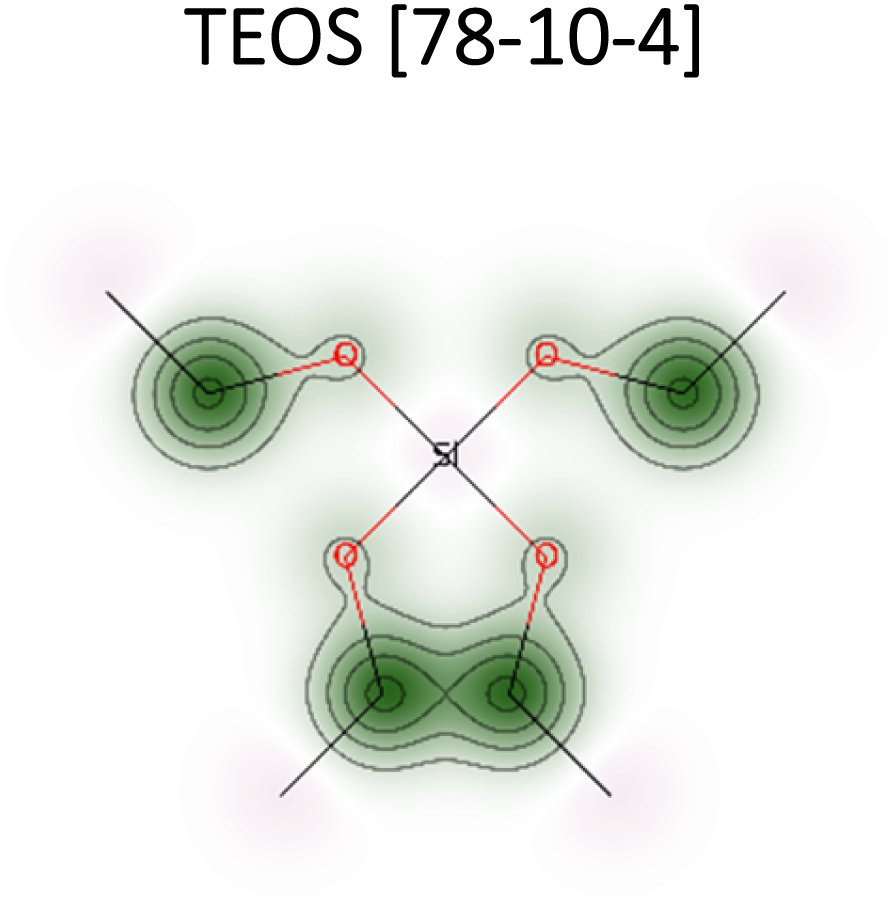	Negative	In	Negative	Negative	In	Negative	High
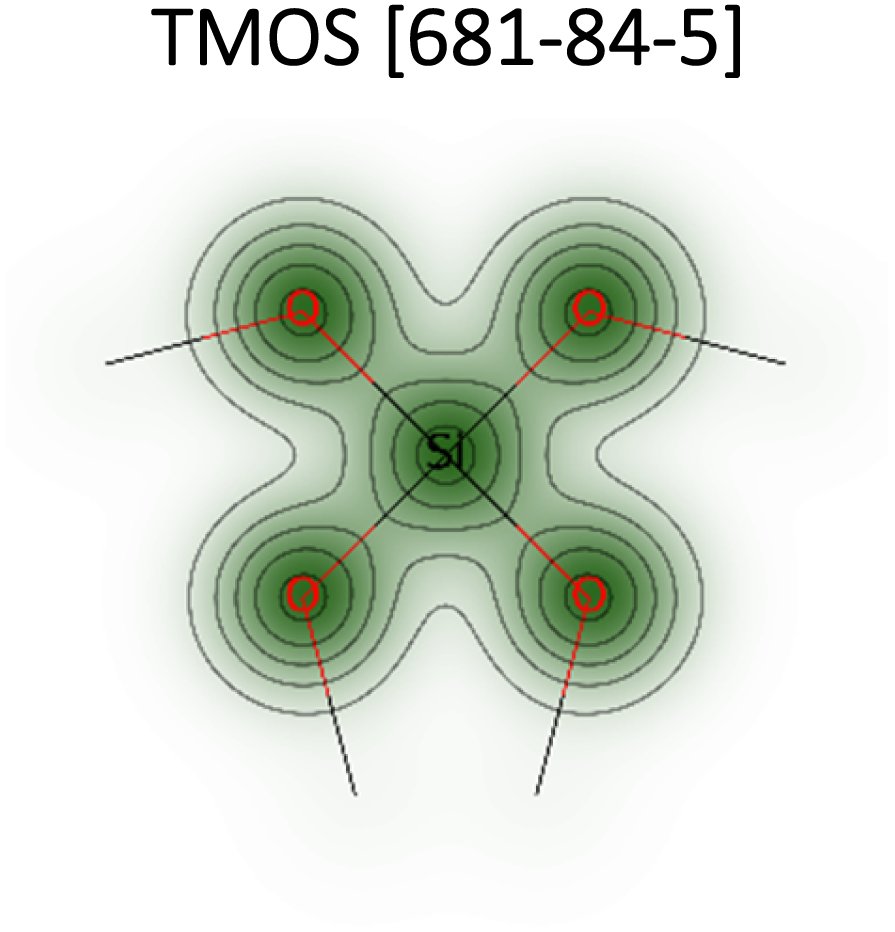	Negative	In	Negative	Negative	In	Negative	High
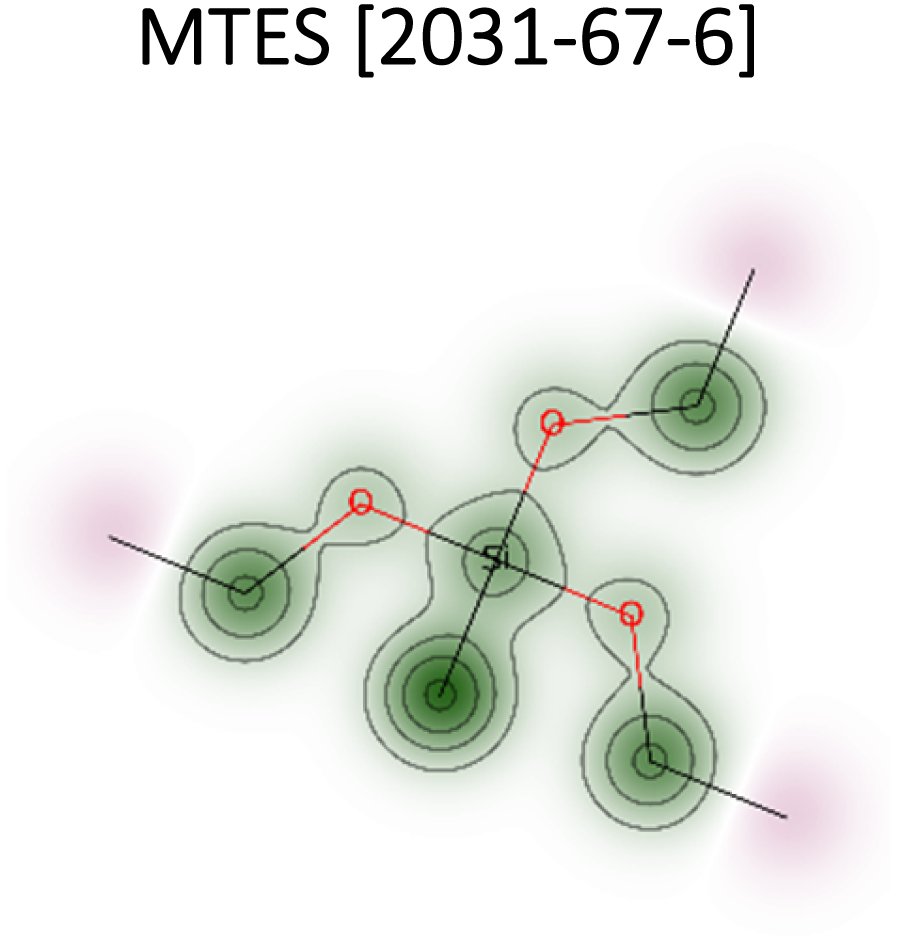	Negative	In	Negative	Negative	In	Negative	High
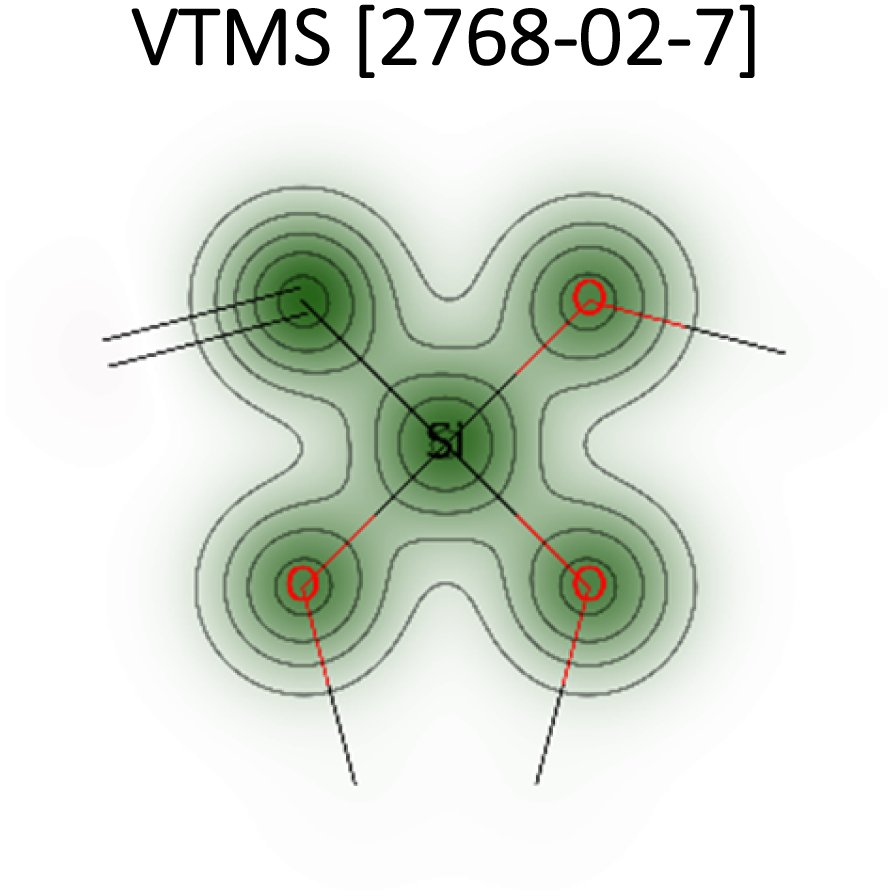	Negative	In	Negative	Negative	In	Negative	High

The models' predictions were validated using observations derived by experimental data, whenever already available. The ECHA's Chemicals database (ECHACHEM)^144^ and EPA's Comptox^145,146^ database were used to this aim. All the investigated chemicals were negative in the Ames test reported in the databases, confirming the predictions of the models (Table S7).

### Web implementation

**Fig. 9:**
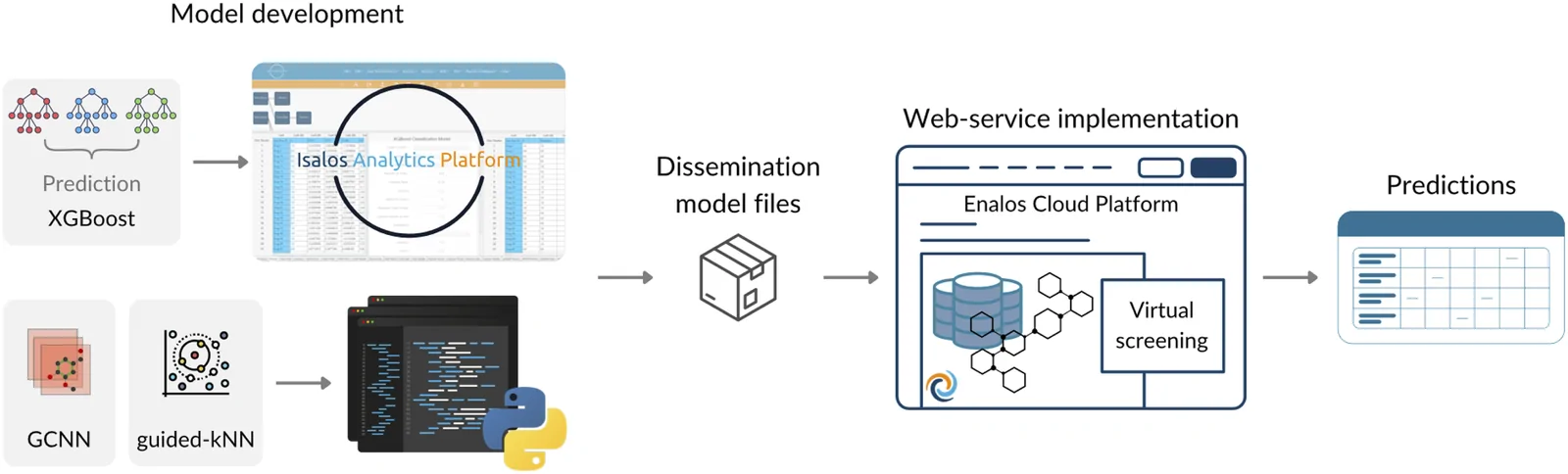
Integration of the models within the Enalos Cloud Platform.

To facilitate the use of the three developed models in computer-aided drug discovery and materials risk assessment applications, we have disseminated them through a user-friendly interface. In brief, Isalos and Python models and scripts were “packed” and integrated into the Pólis web service (Fig. 9), hosted on the Enalos Cloud Platform (https://www.enaloscloud.novamechanics.com/insight/polis/).

**Fig. 10:**
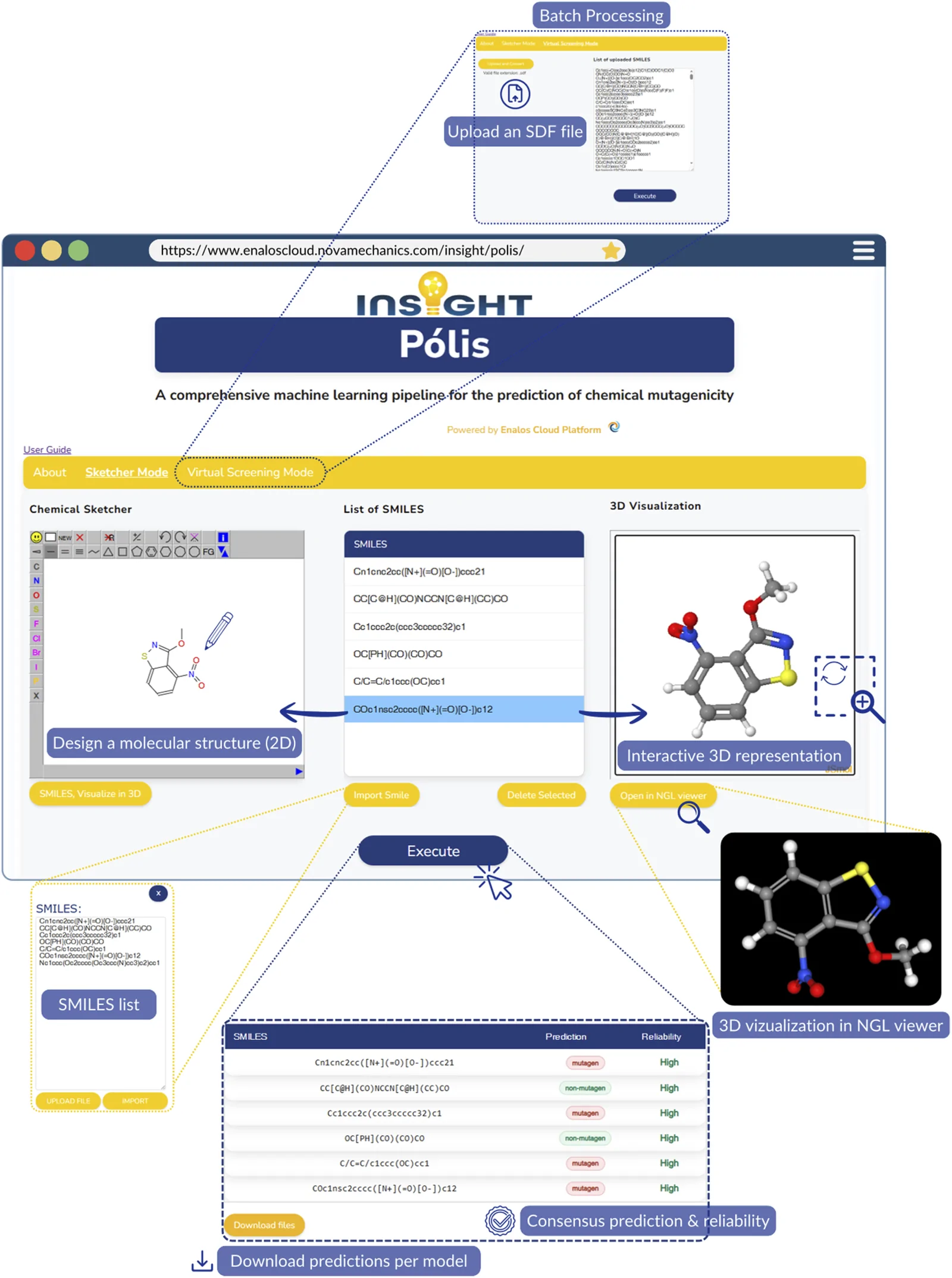
Pólis web service interface: users can sketch query molecules or import them *via* a SMILES list or an SDF file. For each compound the mutagenicity consensus prediction and a notice on the predictions' reliability based on the models' AD limits are displayed.

Users can input one or more compounds of interest either by drawing them using the built-in sketcher or by uploading a list of molecules in SMILES or SDF format. For critical decisions, users are advised to submit fully specified stereochemistry to achieve maximal reliability. The platform provides multiple visualization options, allowing users to inspect compounds in both 2D and 3D (Fig. 10).

Upon submission, mutagenicity predictions for each input structure (“mutagen” – Ames positive or “non-mutagen” – Ames negative) are generated within seconds (Fig. 10). For larger datasets, the calculation of 3D descriptors may take additional time due to the generation of molecular conformers. The results include the consensus prediction, and an assessment of reliability based on the models' AD, as described in previous sections. In case predictions cannot be produced from the guided-kNN and XGBoost models due to the inability to generate 3D conformers, the consensus prediction is based solely on the GCNN model. The results can be downloaded for further analysis, offering flexibility for various research needs. The downloadable files include: the predictions of each model and the consensus outcome, the reliability assessments based on the corresponding AD, the nine nearest training neighbours used in the guided-kNN model, and the canonical SMILES representations of each input compound (sanitized and standardized), to enhance reproducibility.

## Conclusions

In the present research work, a consensus *in silico* approach for chemicals Ames mutagenicity assessment has been developed based on the compounds multi-view representations (molecular fingerprints, 2D, 3D descriptors and graphs) and on three modelling schemes (QSAR/XGBoost, read-across and deep learning models). The proposed guided-kNN read-across approach incorporates two molecular featurization methods that are used for neighbour selection and prediction calculations and can be generalized to any ML problem where a multi-perspective description of observations is available. In this hybrid method molecular structural similarity is measured *via* chemical fingerprint similarity, which guides the selection of neighbouring molecules. Predictions for a query compound are then generated *via* a majority vote scheme where inverse distances between neighbouring molecules are used as weighting factors. In this case distances between neighbours are calculated based on compounds molecular descriptors. Our guided-kNN method achieved accuracy levels comparable to those of the XGBoost and GCNN models, demonstrating that a simpler approach can still deliver reliable results while being significantly faster and less resource-intensive, particularly in terms of training time and hyperparameter optimization, especially when compared to GCNN. A key advantage of the guided-kNN method lies in its ability to offer transparent insights into the classification process. The grouping of similar compounds based on Tanimoto similarity, along with the ability to clearly observe each compound's contribution to the prediction, enhances the model's interpretability from both chemical and algorithmic perspectives, offering a clear and intuitive understanding of the model's decision-making process. Nevertheless, further evaluation of the method and of the encoded hyperspaces (using different fingerprints, *e.g.*, MACCS keys, or combining different fingerprinting methods) across diverse tasks-including those beyond the scope of cheminformatics-will be necessary to fully assess its robustness and identify potential limitations.

Considering that 2D descriptors do not encode the spatial arrangement of molecules, configuration-dependent activity cannot be captured, therefore the inclusion of 3D descriptors addresses this limitation. The presented pipeline for the generation and use of 3D descriptors for Ames mutagenicity prediction can serve as a template for future models and other endpoints, as it consists of a complete, endpoint-agnostic workflow for the generation, application, and stochasticity assessment of 3D molecular descriptors in predictive cheminformatics. Within this pipeline, we retained only compounds with unambiguous stereochemistry. Although this strict filtering led to some loss of information, it was considered necessary so that each descriptor corresponds to a well-defined structure. In addition, as 3D descriptors depend on conformer generation, which is an inherently stochastic procedure, we quantified the resulting variability into the consensus model accuracy, an aspect frequently overlooked but essential for the reproducibility of models trained on 3D descriptors. Overall, the proposed pipeline provides the community with a transparent, transferable, and extensible workflow applicable beyond the present endpoint. Future experimental studies should likewise report complete stereochemical information, and data-management stakeholders should encourage this practice. In this way, it will become possible to perform synergistic studies that yield more meaningful results for the community.

The different methods separately yielded accuracy values between 80–81% in external validation and they were later combined in a majority voting scheme that reached an accuracy of 83% in external validation, close to the average interlaboratory reproducibility rate of results from the Ames test (85%). Next, the application of the consensus model on a blind set yielded accuracy results of 81%, indicating the consistency of the prediction accuracy in real-world applications. An AD analysis was also performed again considering both molecular fingerprints and descriptors. This complementary approach enhances stakeholder confidence in the predictions, by independently defining AD limits for different featurizations, which are then integrated to form a comprehensive assessment of prediction reliability.

A mechanistic interpretation of the models was also provided which associated descriptors used in model development with known toxicophores of mutagenicity (*e.g.*, nitroaromatic and amine groups). To this end, the descriptors selected by the KBest method in the guided-kNN model were compared with those identified through SHAP analysis in the XGBoost model and were interpreted as being associated with mechanisms underlying mutagenic potential. A consistent association was observed between flat, extended molecular structures and an increased risk of mutagenicity. The fragment contribution analysis using the GCNN model for mutagenic compounds further supported this conclusion. An effort was made to conceptually map the relevant descriptors to KEs in the AOPs, specifically leading to KE 185: “Increase, Mutations,” in order to interpret the underlying mechanisms. The descriptors used in modelling were found to align with known KEs, indicating their relevance and contribution to the predictive performance of the models.

Data FAIRness was sought through sharing of the curated and enriched dataset used for model development and validation *via* the ChemPharos database. A standardized MODA document was also prepared, allowing transparency and FAIRification of the modelling steps. The consensus model is made available for the wider community as user-friendly web-service through the Enalos Cloud platform (https://www.enaloscloud.novamechanics.com/insight/polis/) allowing the fast screening chemical compounds to assess their mutagenicity and thus supporting regulatory frameworks and the SSbD of novel materials.

## Author contributions

Dimitra-Danai Varsou: conceptualization, data curation, formal analysis, methodology, software, validation, visualization, writing – original draft preparation, writing – review & editing; Andreas Tsoumanis: validation, software, writing – review & editing; Eleonora Marta Longhin: writing – review & editing; Maria Dusinska: writing – review & editing; Dimitris G. Mintis: writing – review & editing; Panagiotis D. Kolokathis: writing – review & editing; Konstantinos D. Papavasileiou: writing – review & editing; Iseult Lynch: writing – review & editing; Georgia Melagraki: writing – review & editing; Antreas Afantitis: funding acquisition, resources, supervision, writing – review & editing.

## Conflicts of interest

DDV, AT, DGM, PDK, KDP and AA are affiliated with NovaMechanics, a cheminformatics and materials informatics company.

## Abbreviations

AD: Applicability domain
ADMET: Absorption, distribution, metabolism, elimination and toxicity
AI: Artificial intelligence
ANOVA: Analysis of variance
AOP: Adverse outcome pathway
AUC: Area under the curve
CFS: Correlation based feature selection
CNN: Convolutional neural network
CSS: Chemicals strategy for sustainability
CV: Cross-validation
DL: Deep learning
ECFP: Extended-connectivity fingerprints
ECHA: European Chemicals Agency
EMMC: European Materials Modelling Council
ETKDG: Experimental-torsion distance geometry
FAIR: Findable, accessible, interoperable, reusable
GIN: Graph isomorphism network
GCNN: Graph convolutional neural network
GNN: Graph neural network
IATA: Integrated approaches to testing and assessment
KE: Key event
KER: Key event relationship
kNN: *k*-Nearest Neighbours
LOO: Leave-one-out
MCC: Matthews correlation coefficient
ML: Machine learning
MOE: Molecular operating environment
MODA: Modelling data
NAMs: New approach methodologies
NCTR: National Center for Toxicological Research
OECD: Organization for Economic Cooperation and Development
PBF: Plane of best fit
PFAS: Poly- and perfluoroalkyl substances
PFOTS: Perfluorooctyl-triethoxysilane
QSAR: Quantitative structure–activity relationship
QMRF: QSAR model reporting format
REACH: Registration, evaluation, authorisation and restriction of chemicals
ROC: Receiver operating characteristic curve
SHAP: Shapley additive explanations
SMILES: Simplified molecular input line entry system
SSbD: Safe and sustainable by design
SVM: Support vector machines
TDC: Therapeutics data commons
TPE: Tree-structured Parzen Estimator
UFF: Universal force field
XAI: eXplainable AI
XGBoost: Extreme gradient boosting

## Supplementary Material

RA-OLF-D6RA05786A-s001

RA-OLF-D6RA05786A-s002

RA-OLF-D6RA05786A-s003

## Data Availability

The dataset used for the GCNN model development and validation is available *via* the TDC database (https://tdcommons.ai/single_pred_tasks/tox/#ames-mutagenicity). TDC provides Python functions for data retrieval and splitting to facilitate the training of machine learning models (see also QMRF reports). The curated and enriched with 2D and 3D molecular descriptors dataset used for read-across and QSAR model development and validation is available in tabular format *via* the ChemPharos database (https://db.chempharos.eu/datasets/Datasets.zul?datasetID=ds18). The curated dataset is also available through zenodo (https://doi.org/10.5281/zenodo.18937797) under the Creative Commons Attribution 4.0 International license. The Pólis tool and its manual are freely available *via* the Enalos Cloud Platform at https://enaloscloud.novamechanics.com/insight/polis/. Pólis can be also accessed remotely using APIs (https://www.enaloscloud.novamechanics.com/insight/swagger-ui/) to enhance interoperability and reusability. The source code is proprietary and not publicly available. To facilitate transparency the workflow and the independent models used for the Pólis tool development are thoroughly documented in the standardized MODA and QMRF reports which are available free of charge as supplementary information (SI) of this article. Supplementary information: a schematic figure of the data curation and featurization process, Tanimoto similarities within the training compounds, a table describing the selected descriptors and validation results for the model are also provided. A QMRF report and a MODA description of the Pólis model are also made available to increase model transparancy and support FAIR models and software. See DOI: https://doi.org/10.1039/d6ra05786a.
