# Supplementary material for: From QSAR to deep learning: an interpretable comprehensive pipeline with a read-across approach for mutagenicity prediction via the Enalos Cloud Platform
Source: RSC Adv. 2026 Sep 9;16(43):53776–805. doi: 10.1039/d6ra05786a (PMC13557173; doi:10.1039/d6ra05786a)
Supplement: Supplementary file 1 [file RA-016-D6RA05786A-s001.pdf]

Supplementary Information (SI) for RSC Advances.
This journal is © The Royal Society of Chemistry 2026

Moda powered by **Enalos Cloud Platform**

MODA for

Prediction of chemical mutagenicity based on the Ames test

Simulated in project

Pólis

OVERVIEW of the SIMULATION			
1	**USER CASE**	A comprehensive pipeline for Ames Mutagenicity prediction.	
2	**CHAIN OF MODELS**	Data Transformation 1	Data curation<br>Data Transformation
		Data Transformation 2	Molecular fingerprints<br>Data Transformation
		Data Transformation 3	2D descriptors<br>Data Transformation
		Model 4	3D descriptors<br>Physics based Model
		Data Transformation 5	Gaussian normalization<br>Data Transformation
		Data Transformation 6	Molecular graphs<br>Data Transformation
		Model 7	guided-kNN<br>Data based Model
		Model 8	GCNN<br>Data based Model
		Model 9	XGBoost<br>Data based Model
		Model 10	Consensus<br>Data based Model
		Model 11	Applicability domain (guided-kNN)<br>Data based Model
		Model 12	Applicability domain (XGBoost)<br>Data based Model
		Model 13	Reliability assessment<br>Data based Model
3	**PUBLICATION PEER -**	DOI provided: No	

	REVIEWINGTHE DATA	
**4**	**ACCESS CONDITIONS**	Access type: Free<br>Owner of workflow: NovaMechanics MIKE<br>Workflow access link:<br>https://www.enaloscloud.novamechanics.com/insight/polis/
**5**	**WORKFLOW AND ITS RATIONALE**	New approach methodologies (NAMs), including computational approaches, provide non-animal alternatives for testing novel materials and chemicals. This study highlights the potential of *in silico* NAMs, which can contribute to the development of novel Safe and Sustainable by Design (SSbD) chemicals and substances by identifying potentially hazardous ones at an early stage. A consensus strategy integrating different chemical representations (molecular fingerprints, 2D and 3D descriptors and molecular graphs) and modelling methods (QSAR, read-across and deep learning models) is employed to predict the mutagenic profile of chemical compounds. The predictions from the three models are integrated into a majority voting scheme to enhance the overall predictive accuracy and reducing individual model biases.

Workflow picture

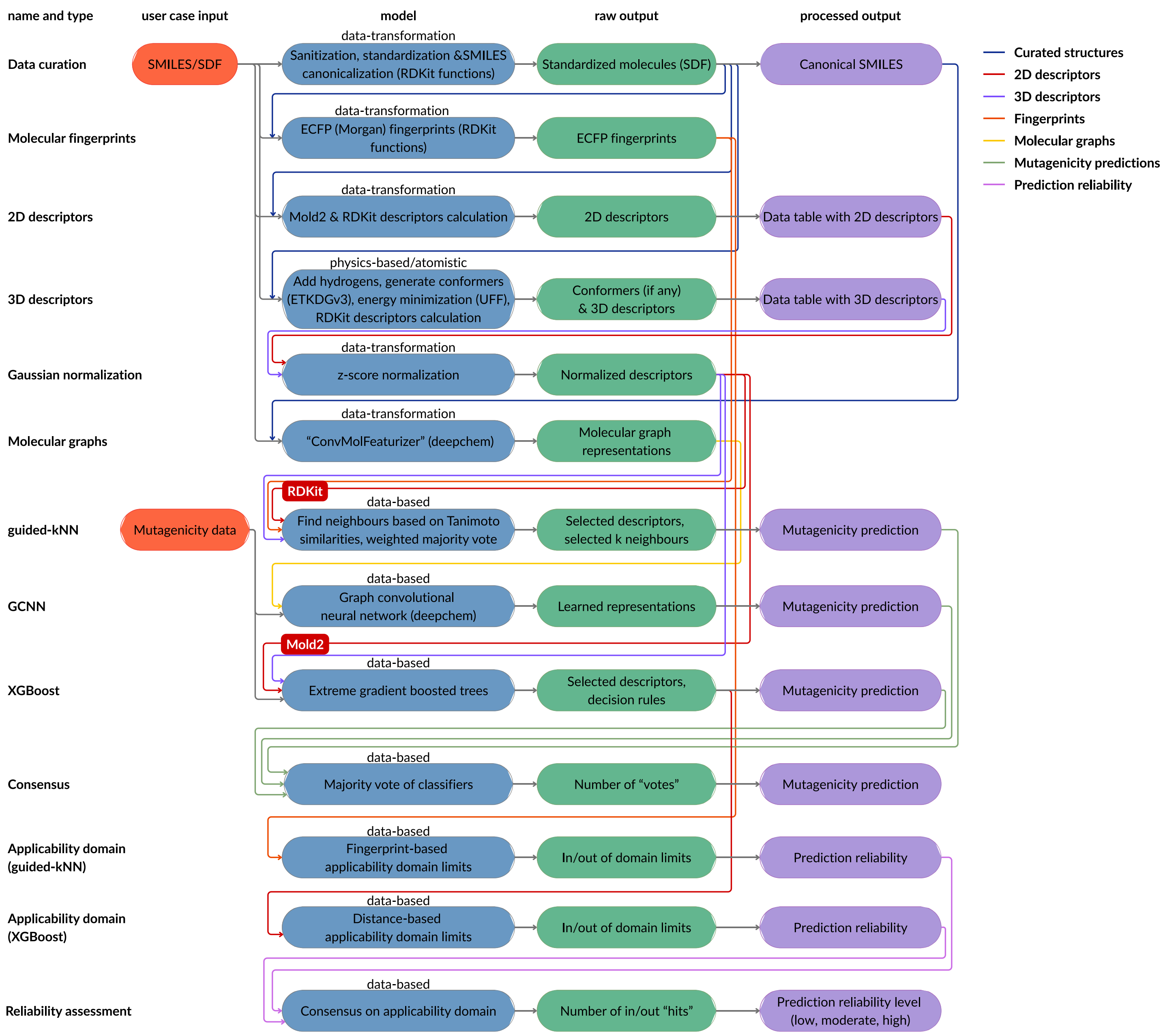

Each physics-based model used in this simulation is to be documented in four chapters:

1. Aspect of the User Case or system simulated with this model
2. Model: Please make sure the notions of Physics Equation and Materials Relation are properly understood.
   - Tightly coupled models can be written up collectively in one set of four tables. To solve tightly coupled PE one matrix is set up and solved in one go.
   - For continuum models the PE is often the conservation equations coded up in bought software packages.
   - Often the MR is established by the modeller.
3. Computational aspects include also documentation of how the user case specifications are translated into computer language.
4. Post processing documents on how the raw output of one simulation is processed into input for the next simulation. This information given under 4.1 in the first model will be the same as the "simulated input" information under 2.4 for the next model. This is the essence of model inter- operability!
5. Pre-processing before the first model can be depicted in pink as it is considered to be part of the user-case.

Each data-based model in this simulation is to be documented in three chapters:

1. Aspect of the User Case or system simulated with this data-based model
2. Data-based Model
3. Computational detail of the data mining operation

Data Transformation 1

Data curation

Aspect of the User Case/System		
**1.1**	**Aspect of the User Case to be simulated**	The compounds used for modelling and validation are sanitized, standardized (normalization of functional groups, retain largest organic fragments) to reduce noise in the cheminformatics analysis, thereby enhancing model performance and interpretability. Later pre-processed molecules are transformed into canonical SMILES using RDKit to ensure consistency and eliminate representational redundancies.
**1.2**	**Material**	Small organic molecules
**1.3**	**Geometry**	
**1.4**	**Time Lapse**	
**1.5**	**Manufacturing process or in service conditions**	
**1.6**	**Publication on this data**	

Data Transformation			
2.0	**Equation type and name**		
2.1	**Database and type**		
2.2	**Equation**	Hypothesis	
		Physical quantities	

Computational detail		
3.1	**Numerical Operations**	mol = Chem.MolFromSmiles(smiles, sanitize=True)<br>cm = rdMolStandardize.Normalize(mol)<br>standardized_mol =<br>rdMolStandardize.FragmentParent(cm)<br>Chem.SanitizeMol(standardized_mol)
3.2	**Software tool**	RDKit v2024.3.6, Chem package
3.3	**Margin Of Error**	

Data Transformation 2

Molecular fingerprints

Aspect of the User Case/System		
1.1	**Aspect of the User Case to be simulated**	Morgan fingerprints are used (also known as extended-connectivity fingerprints, ECFP) encode the molecular structures by iteratively assessing the atoms local environment. In fact, count fingerprints are used to represent the frequency of nonzero counts for substructures in the molecules.
1.2	**Material**	Small organic molecules
1.3	**Geometry**	
1.4	**Time Lapse**	
1.5	**Manufacturing process or in service conditions**	
1.6	**Publication on this data**	

Data Transformation		
2.0	**Equation type and name**	

**2.1**	**Database and type**		
**2.2**	**Equation**	Hypothesis	rdFingerprintGenerator.<br>GetMorganGenerator(radius=4,<br>fpSize=2048)
		Physical quantities	

**Computational detail**		
**3.1**	**Numerical Operations**	
**3.2**	**Software tool**	RDKit v2024.3.6, Chem package
**3.3**	**Margin Of Error**	

Data Transformation 3

2D descriptors

Aspect of the User Case/System		
**1.1**	**Aspect of the User Case to be simulated**	In this work, Mold2 and RDKit descriptors are employed. Mold2 software, developed by the National Center for Toxicological Research (NCTR), was used to obtain 777 unique molecular descriptors that capture the properties of small molecules, based on their 2D chemical structure. RDKit software calculates 210 descriptors that encode physicochemical, topological, and structural properties of substances, again based on their 2D structure.
**1.2**	**Material**	Small organic molecules
**1.3**	**Geometry**	
**1.4**	**Time Lapse**	
**1.5**	**Manufacturing process or in service conditions**	
**1.6**	**Publication on this data**	

Data Transformation			
**2.0**	**Equation type and name**		
**2.1**	**Database and type**		
**2.2**	**Equation**	Hypothesis	
		Physical quantities	

Computational detail		
**3.1**	**Numerical Operations**	
**3.2**	**Software tool**	RDKit v2024.3.6, Chem package<br>Enalos+ Mold2 KNIME node
**3.3**	**Margin Of Error**	

Model 4

3D descriptors

Aspect of the User Case/System to be Simulated			
1.1	**Aspect of the User Case to be simulated**	3D Molecular Descriptor Generation via Energy-Minimized Conformers: To generate reproducible 3D coordinates for a molecule, select the energetically most favorable conformer via forcefield minimization, and compute a set of 3D molecular descriptors that characterize molecular shape, size, and electronic features.	
1.2	**Material**	Small organic molecules	
1.3	**Geometry**		
1.4	**Time Lapse**		
1.5	**Manufacturing process or in service conditions**		
1.6	**Publication on this data**		

Generic Physics Of The Model Equation			
2.0	**Model type and name**	Conformers generation is performed using the Experimental-Torsion Distance Geometry (ETKDG) algorithm which combines the distance geometry approach and knowledge-based torsion-angle preferences to guarantee chemically reasonable geometries.<br><br>Energy minimization is achieved using the Universal Force Field (UFF). This method calculates interatomic potentials (bond stretching, angle bending, torsional, and van der Waals interactions) that govern the molecular geometry.	
2.1	**Model entity**		
2.2	**Model Physics/ Chemistry equation PE**	Equation	UFF force field parameters are used.
		Physical quantities	
2.3	**Materials relations**	Relation	UFF force field parameters are used.
		Physical quantities /descriptors for each MR	

**2.4**	**Simulated input**	A molecule provided as an RDKit Mol object. Hydrogens are added to the molecule.

**Solver and Computational translation of the specifications**			
**3.1**	**Numerical Solver**	1. Conformer Embedding: ETKDGv3 algorithm is used to embed multiple conformers.<br>2. Energy Minimization: Each generated conformer is optimized using the UFF by calling AllChem.UFFOptimizeMolecule. The UFF force field calculates the potential energy (ff.CalcEnergy()), and the conformer with the minimum energy is selected.	
**3.2**	**Software tool**	RDKit v2024.3.6, Chem package<br>Force Field: Universal Force Field (UFF)	
**3.3**	**Time step**	no timestep	
**3.4**	**Computational Representation**	Physics Equation,<br>Material Relations,<br>Material	
**3.5**	**Computational boundary conditions**		
**3.6**	**Additional Solver Parameters**	For conformers generation:<br>randomSeed = 0xf00d<br>maxIterations = 1000<br>pruneRmsThresh = 0.5<br>useSmallRingTorsions = False<br>useMacrocycleTorsions = True<br>enforceChirality = True<br>clearConfs = True<br>useRandomCoords = False<br>useBasicKnowledge = True	

Post processing

The “raw output” calculated by the model consists per definition of values for the physics variable in the PE(s). This variable is already specified in 2.2 and this raw output will appear in your dark green circle in the workflow picture.

- to calculate values for physics variables for different entities of the next model. E.g. the output can be homogenised for larger volumes
    - in the form of a MR for the next model
    - into a Descriptor Rule that is the final output of the total simulation.

This processed output will appear in your light green circle in the workflow picture and also in 2.4 of the next model (if there is one).

The methodology (often including physics) used to do this post processing calculation is to be documented in 4.2.

Solver and Computational translation of the specifications		
**4.1**	**The processed output**	
**4.2**	**Methodologies**	
**4.3**	**Margin Of Error**	

Data Transformation 5

Gaussian normalization

Aspect of the User Case/System		
**1.1**	**Aspect of the User Case to be simulated**	2D and 3D descriptors are standardized with z-score (Gaussian) transformation to guarantee that they participate equally to the analysis. Standardization is performed only on the training data, and the parameters are later applied on the validation and test sets to avoid data leakage. Standardization is necessary for the guided-kNN methodology as Euclidean distances are involved in the prediction. Standardization is optional for tree-based models (like XGBoost) nonetheless, as the final selection of the QSAR model is made through a multi-model pipeline, normalization is performed upfront to keep the workflow modular and clean.
**1.2**	**Material**	Small organic molecules
**1.3**	**Geometry**	
**1.4**	**Time Lapse**	
**1.5**	**Manufacturing process or in service conditions**	
**1.6**	**Publication on this data**	

Data Transformation			
2.0	Equation type and name	$$\tilde{x}_i = \frac{x_i - \overline{\boldsymbol{X}}}{s}$$ where $\tilde{x}_i$ and x_i are the standardized and the original values of the ith instance of $\boldsymbol{X}$ feature vector respectively, $\bar{X}$ is the mean value of $\boldsymbol{X}$ and, s is the standard deviation of $\boldsymbol{X}$.	
2.1	Database and type		
2.2	Equation	Hypothesis	Linear transformation, normalized values present a mean value equal to 0 and standard deviation equal to 1
		Physical quantities	

Computational detail		
3.1	Numerical Operations	
3.2	Software tool	
3.3	Margin Of Error	

Data Transformation 6

Molecular graphs

Aspect of the User Case/System		
1.1	Aspect of the User Case to be simulated	Molecules can be represented as undirected graphs, where atoms are the graph nodes and bonds are the edges. This mathematical representation allows for the development of graph convolutional neural networks (GCNNs) that can be trained to predict molecular properties and/or activity.
1.2	Material	Small organic molecules
1.3	Geometry	
1.4	Time Lapse	
1.5	Manufacturing process or in service conditions	
1.6	Publication on this data	

Data Transformation			
**2.0**	**Equation type and name**		
**2.1**	**Database and type**		
**2.2**	**Equation**	Hypothesis	
		Physical quantities	featurizer= dc.feat.ConvMolFeaturizer(per_atom_fragmentation = False)

Computational detail		
**3.1**	**Numerical Operations**	
**3.2**	**Software tool**	deepchem v2.8.1
**3.3**	**Margin Of Error**	

Model 7

guided-kNN

Aspect of the User Case/System to be Simulated		
**1.1**	**Aspect of the User Case to be simulated**	[Endpoint]<br>Ames Mutagenicity<br><br>[Comment on endpoint]<br>Categorical endpoint, each compound is labelled (TDC database) as either "1" (mutagen) or "0" (non-mutagen)<br><br>[Endpoint units]<br>NA<br><br>[Experimental protocol]<br>See TDC database:<br>https://tdcommons.ai/single_pred_tasks/tox#ames-mutagenicity<br><br>An additional filtering of the chemical compounds is performed so that the 3D descriptors correspond as closely as possible to the input compounds, excluding those with ambiguous stereochemistry. The RDKit EnumerateStereoisomers module is used to search for possible stereoisomers for each one of the compounds of the training, validation and test sets. Compounds that yield multiple stereoisomers due to unassigned stereocenters, are filtered out.<br><br>[Endpoint data quality and variability]

<table>
<tr><td></td><td></td><td>
<table>
<tr><th>Subset</th><th>All</th><th>Mutagen [1]</th><th>Non-mutagen [0]</th></tr>
<tr><td>Train</td><td>3724</td><td>2090</td><td>1634</td></tr>
<tr><td>Validation</td><td>549</td><td>325</td><td>224</td></tr>
<tr><td>Test</td><td>1089</td><td>601</td><td>488</td></tr>
<tr><td>Total</td><td>5362</td><td>3016</td><td>2346</td></tr>
</table>
</td></tr>
<tr><td>1.2</td><td>Material</td><td>Small organic molecules</td></tr>
<tr><td>1.3</td><td>Geometry</td><td></td></tr>
<tr><td>1.4</td><td>Time Lapse</td><td></td></tr>
<tr><td>1.5</td><td>Manufacturing process or in service conditions</td><td></td></tr>
<tr><td>1.6</td><td>Publication on this data</td><td></td></tr>
</table>

<table>
<tr><th colspan="4">The Data-based Model</th></tr>
<tr><td>2.0</td><td>Equation type and name</td><td colspan="2">[Explicit Algorithm]
1. Each molecule is encoded with a chemical fingerprint and Tanimoto similarity is calculated between query molecules and training set molecules.
2. The k neighbours are selected based on their Tanimoto similarity ranking (k=9).
3. The class prediction for the query molecule is the weighted majority vote of the k neighbouring molecules. Weights are proportional to the inverse distances between these neighbours, which are calculated using their molecular descriptors.

[Data availability]
Model development is based on the Ames Mutagenicity dataset directly retrieved by the Therapeutics Data Commons (TDC) platform. TDC offers for each dataset, split functions in Python to support training of machine learning (ML) models. In this course, the TDC’s protocol is followed for random data splitting into train (70%), validation (10%) and test (20%) subsets, for the development of our ML and DL classification models. The descriptors used for modelling and validation can be found in the ChemPharos DB (https://db.chempharos.eu/datasets/Datasets.zul?datasetID=ds18).</td></tr>
<tr><td>2.1</td><td>Database and type</td><td colspan="2">ChemPharos DB (https://db.chempharos.eu/datasets/Datasets.zul?datasetID=ds18)</td></tr>
<tr><td rowspan="2">2.2</td><td rowspan="2">Equation</td><td>Hypothesis</td><td>[Type of model]
Instance-based/read-across</td></tr>
<tr><td>Physical quantities</td><td>[Descriptors in the model]
<table>
<tr><td>SlogP_VSA8</td><td>A MOE-type descriptor that sums the van der Waals surface area (VSA) contributions of atoms assigned to bin 8 based on their atomic contributions to the logP (expressing hydrophobicity)</td></tr>
</table>
</td></tr>
</table>

			**fr_nitro**	Number of nitro groups
			**NumAromaticRings**	Number of aromatic rings in the molecule
			**HallKierAlpha**	The Hall-Kier alpha value for a molecule
			**RingCount**	Total number of rings in the molecule
			**fr_bicyclic**	Number of bicyclic rings
			**NumAromaticCarbocycles**	Number of aromatic rings containing only carbon atoms.
			**fr_benzene**	Number of benzene rings
			**BertzCT**	A topological index meant to quantify complexity of molecules
			**fr_nitro_arom_nonortho**	Number of non-ortho nitro benzene ring substituents
			**fr_nitro_arom**	Number of nitro benzene ring substituents
			**PBF**	Plane of best fit
			**PEOE_VSA2**	A MOE-type descriptor that sums the van der Waals surface area (VSA) contributions of atoms assigned to bin 2 based on their partial equalization of orbital electronegativity (PEOE) charge values
			**AvgIpc**	Average information content per atom
			**fr_NH0**	Number of Tertiary amines
			**SMR_VSA5**	A MOE-type descriptor that sums the van der Waals surface area (VSA) contributions of atoms assigned to bin 5 based on their atomic contributions to the MR (molecular refractivity)
			**SlogP_VSA10**	A MOE-type descriptor that sums the van der Waals surface area (VSA) contributions of atoms assigned to bin 10 based on their atomic contributions to the logP (expressing hydrophobicity)
			**SpherocityIndex**	An anisometry descriptor, it varies from zero for flat molecules, to one for totally spherical molecules
			**SMR_VSA7**	A MOE-type descriptor that sums the van der Waals surface area (VSA) contributions of atoms assigned to bin 7 based on their atomic contributions to the MR (molecular refractivity)
			**SlogP_VSA5**	A MOE-type descriptor that sums the van der Waals surface area (VSA) contributions of atoms assigned to bin 5 based on their

				atomic contributions to the logP (expressing hydrophobicity)
			**SMR_VSA10**	A MOE-type descriptor that sums the van der Waals surface area (VSA) contributions of atoms assigned to bin 10 based on their atomic contributions to the MR (molecular refractivity)
			**SlogP_VSA4**	A MOE-type descriptor that sums the van der Waals surface area (VSA) contributions of atoms assigned to bin 4 based on their atomic contributions to the logP (expressing hydrophobicity)
			**VSA_EState8**	A MOE-type descriptor that sums the electrotopological state (EState) indices of atoms assigned to bin 8 based on their van der Waals surface area (VSA) contributions
			**FractionCSP3**	Fraction of sp^3-hybridized carbon atoms
			**VSA_EState6**	A MOE-type descriptor that sums the electrotopological state (EState) indices of atoms assigned to bin 6 based on their van der Waals surface area (VSA) contributions
			**PEOE_VSA13**	A MOE-type descriptor that sums the van der Waals surface area (VSA) contributions of atoms assigned to bin 13 based on their partial equalization of orbital electronegativity (PEOE) charge values
			**qed**	Quantitative Estimate of Drug-likeness
			**FpDensityMorgan3**	Fingerprint density for Morgan fingerprints (radius=3)
			**SlogP_VSA6**	A MOE-type descriptor that sums the van der Waals surface area (VSA) contributions of atoms assigned to bin 6 based on their atomic contributions to the logP (expressing hydrophobicity)
			**NOCount**	Number of Nitrogen and Oxygen atoms
			**VSA_EState3**	A MOE-type descriptor that sums the electrotopological state (EState) indices of atoms assigned to bin 3 based on their van der Waals surface area (VSA) contributions
			**TPSA**	Topological polar surface area

			**VSA_EState4**	A MOE-type descriptor that sums the electrotopological state (EState) indices of atoms assigned to bin 4 based on their van der Waals surface area (VSA) contributions
			**NumHAcceptors**	Number of Hydrogen Bond Acceptors
			**fr_unbrch_alkane**	Number of unbranched alkanes of at least 4 members (excludes halogenated alkanes)
			**Chi4n**	A valence molecular connectivity index calculated based on Rev. Comp. Chem., vol 2, 367-422 (1991).
			**PEOE_VSA8**	A MOE-type descriptor that sums the van der Waals surface area (VSA) contributions of atoms assigned to bin 8 based on their partial equalization of orbital electronegativity (PEOE) charge values
			**VSA_EState7**	A MOE-type descriptor that sums the electrotopological state (EState) indices of atoms assigned to bin 7 based on their van der Waals surface area (VSA) contributions
			**EState_VSA10**	A MOE-type descriptor that sums the van der Waals surface area (VSA) contributions of atoms assigned to bin 10 based on their electrotopological state (EState) index
			**fr_COO2**	Number of carboxylic acids
			**Chi1**	A molecular connectivity index calculated from equations (1),(11) and (12) of Rev. Comp. Chem. vol 2, 367-422, (1991)
			**fr_ArN**	Number of aromatic nitrogens
			**BalabanJ**	A chemical distance-based topological index from Chem. Phys. Lett. vol 89, 399-404 (1982).
			**VSA_EState2**	A MOE-type descriptor that sums the electrotopological state (EState) indices of atoms assigned to bin 2 based on their van der Waals surface area (VSA) contributions
			**NumRotatableBonds**	Number of Rotatable Bonds
			**HeavyAtomCount**	Number of heavy atoms of a molecule

			**SlogP_VSA3**	A MOE-type descriptor that sums the van der Waals surface area (VSA) contributions of atoms assigned to bin 3 based on their atomic contributions to the logP (expressing hydrophobicity)
			**Chi3n**	A valence molecular connectivity index calculated based on Rev. Comp. Chem., vol 2, 367-422 (1991).
			**NumHeteroatoms**	Number of Heteroatoms
			**fr_aniline**	Number of anilines

[Descriptor selection]
Mutual information filtering & KBest
[Algorithm and descriptor generation]
RDKit v2024.3.6, Chem package

[Software name and version for descriptor and algorithm generation]
RDKit v2024.3.6, Chem package

[Chemicals/ Descriptors ratio]
3724 compounds/50 descriptors

Computational detail of data mining operation

3.1	**Numerical Operations**	
3.2	**Software tool**	Python
3.3	**Margin Of Error**	[Statistics for goodness-of-fit (validation set)]

MCC	0.602
Accuracy	0.807
Sensitivity/Recall	0.828
Specificity	0.777
Precision	0.843

[Robustness – Statistics obtained by leave-one-out cross-validation (training set)]

MCC	0.569
Accuracy	0.788
Sensitivity/Recall	0.811
Specificity	0.758
Precision	0.811

[Robustness – Statistics obtained by Y-scrambling]

Y-Randomization	Accuracy	MCC
# 1	0.495	-0.038

# 2	0.522	0.018
# 3	0.472	-0.087
# 4	0.516	0.011
# 5	0.496	-0.035

[Predictivity – Statistics obtained by external validation]

MCC	0.599
Accuracy	0.802
Sensitivity/Recall	0.845
Specificity	0.749
Precision	0.806

Model 8

GCNN

Aspect of the User Case/System to be Simulated

1.1 Aspect of the User Case to be simulated

[Endpoint]
Ames Mutagenicity

[Comment on endpoint]
Categorical endpoint, each compound is labelled (TDC database) as either “1” (mutagen) or “0” (non-mutagen)

[Endpoint units]
NA

[Experimental protocol]
See TDC database:
https://tdcommons.ai/single_pred_tasks/tox#ames-mutagenicity

[Endpoint data quality and variability]

Subset	All	Mutagen [1]	Non-mutagen [0]
Train	5076	2741	2335
Validation	726	415	311
Test	1453	795	658
Total	7255	3951	3304

1.2 Material

Small organic molecules

1.3 Geometry

1.4 Time Lapse

1.5 Manufacturing process or in service conditions

1.6 Publication on this data

The Data-based Model			
2.0	**Equation type and name**	[Explicit Algorithm]<br>Convolutional neural networks (CNNs) are widely used in processing and modelling images, speech, and time-series data because convolutional layers take advantage of hierarchical patterns and extract high-level features from input data, leading to powerful representational capabilities. Early layers in CNNs detect small-scale local patterns, while deeper layers capture larger, more abstract features. Graph convolutional neural networks (GCNNs) extend CNNs to graph-structured data by leveraging the inherent graph topology. In the context of cheminformatics, GCNNs combine information from neighbouring nodes—bonded atoms in a molecule—in a convolutional manner, allowing for effective learning directly on molecular graphs.<br><br>[Data availability]<br>Model development is based on the Ames Mutagenicity dataset directly retrieved by the Therapeutics Data Commons (TDC) platform. TDC offers for each dataset, split functions in Python to support training of machine learning (ML) models. In this course, the TDC's protocol is followed for random data splitting into train (70%), validation (10%) and test (20%) subsets, for the development of our ML and DL classification models.	
2.1	**Database and type**		
2.2	**Equation**	Hypothesis	[Type of model]<br>Graph convolutional neural network
		Physical quantities	Molecular graphs

Computational detail of data mining operation		
3.1	**Numerical Operations**	The GCNN model treats each molecule as a graph and processes these graphs through multiple GraphConv layers that implements the graph convolution. Each GraphConv layer is followed by BatchNormalization to stabilize training and Dropout to reduce overfitting. A GraphPool layer then performs max pooling over the feature vectors of atoms in a neighbourhood.<br><br>The pooled features of the last GraphConv layers are passed through a fully connected Dense layer with ReLU activation function for further refinement. Normalization and dropout layers are introduced at this stage, as well. A GraphGather layer then aggregates the entire graph's

		representation. Finally, a Dense layer reshapes the output (via Reshape) and applies a softmax activation function, yielding probability distributions over the two classes. A probability closer to 1 is assigned to mutagenic compounds and a probability closer to 0 to non-mutagenic ones. The model is trained using the AdamW optimizer - combining adaptive learning rates with weight decay- and a categorical cross-entropy loss to update the network's weights.<br><br>A Python generator function is used in order to convert the featurized molecules into numpy arrays that can be used as input to the GraphConv, GraphPool, and GraphGather layers.
**3.2**	**Software tool**	deepchem v2.8.1, Keras and TensorFlow v2.15.0
**3.3**	**Margin Of Error**	[Statistics for goodness-of-fit (validation set)]

MCC	0.656
Accuracy	0.831
Sensitivity/Recall	0.841
Specificity	0.817
Precision	0.860

[Robustness – Statistics obtained by Y-scrambling]

Y-Randomization	Accuracy	MCC
# 1	0.531	0.064
# 2	0.480	-0.030
# 3	0.539	0.041
# 4	0.515	0.004
# 5	0.522	0.036

[Predictivity – Statistics obtained by external validation]

MCC	0.613
Accuracy	0.808
Sensitivity/Recall	0.815
Specificity	0.799
Precision	0.831

Model 9

XGBoost

Aspect of the User Case/System to be Simulated		
**1.1**	**Aspect of the User Case to be simulated**	[Endpoint]<br>Ames Mutagenicity

		[Comment on endpoint]<br>Categorical endpoint, each compound is labelled (TDC database) as either “1” (mutagen) or “0” (non-mutagen)<br><br>[Endpoint units]<br>NA<br><br>[Experimental protocol]<br>See TDC database:<br>https://tdcommons.ai/single_pred_tasks/tox#ames-mutagenicity<br><br>An additional filtering of the chemical compounds is performed so that the 3D descriptors correspond as closely as possible to the input compounds, excluding those with ambiguous stereochemistry. The RDKit EnumerateStereoisomers module is used to search for possible stereoisomers for each one of the compounds of the training, validation and test sets. Compounds that yield multiple stereoisomers due to unassigned stereocenters, are filtered out.<br><br>[Endpoint data quality and variability]
1.2	**Material**	Small organic molecules
1.3	**Geometry**	
1.4	**Time Lapse**	
1.5	**Manufacturing process or in service conditions**	
1.6	**Publication on this data**	

Subset	All	Mutagen [1]	Non-mutagen [0]
Train	3724	2090	1634
Validation	549	325	224
Test	1089	601	488
Total	5362	3016	2346

The Data-based Model

2.0	**Equation type and name**	[Explicit Algorithm]<br>The XGBoost open-source library supports ensemble machine learning methods based on decision tree models. In an iterative procedure, trees are added to the ensemble so as to reduce the prediction error (loss) of previous models (in our case the objective function was the minimization of softmax probability loss).

Hyperparameters	Optimized hyperparameters
Booster	gbtree
Tree method	auto

		Number of trees: 100<br>Maximum depth: 11<br>Learning rate (eta): 0.25<br>alpha: 4<br>lambda: 5<br>gamma: 0<br>Minimum child weight: 1<br>Subsampling rate: 0.8<br><br>[Data availability]<br>Model development is based on the Ames Mutagenicity dataset directly retrieved by the Therapeutics Data Commons (TDC) platform. TDC offers for each dataset, split functions in Python to support training of machine learning (ML) models. In this course, the TDC's protocol is followed for random data splitting into train (70%), validation (10%) and test (20%) subsets, for the development of our ML and DL classification models. The descriptors used for modelling and validation can be found in the ChemPharos DB (https://db.chempharos.eu/datasets/Datasets.zul?datasetID=ds18).
2.1	**Database and type**	ChemPharos DB
2.2	**Equation**	Hypothesis: [Type of model] Decision trees/XGBoost

Parameter	Value
Number of trees	100
Maximum depth	11
Learning rate (eta)	0.25
alpha	4
lambda	5
gamma	0
Minimum child weight	1
Subsampling rate	0.8

Physical quantities: [Descriptors in the model]

**D123**	Average of molecular weight
**D173**	Mohar order-2 index
**D186**	Average vertex distance connectivity index
**D279**	Total information content order-4 index
**D282**	Structural information content order-1 index
**D287**	Complementary information content order-0 index
**D297**	Bond information content order-4 index
**D349**	Molecular topological path index of order 09
**D357**	Molecular topological multiple path index of order 09
**D358**	Molecular topological multiple path index of order 10
**D366**	Sum of topological distance between the vertices N and N
**D367**	Sum of topological distance between the vertices N and P
**D413**	Walk-returning count order-5
**D477**	Geary topological structure autocorrelation length-7 weighted by atomic polarizabilities
**D478**	Geary topological structure autocorrelation length-8 weighted by atomic polarizabilities
**D532**	Lowest eigenvalue from Burdex matrix weighted by masses order-1
**D533**	Lowest eigenvalue from Burdex matrix weighted by masses order-2

			**D534** — Lowest eigenvalue from Burdex matrix weighted by masses order-3<br>**D535** — Lowest eigenvalue from Burdex matrix weighted by masses order-4<br>**D564** — Highest eignevalue from Burdex matrix weighted by masses order-1<br>**D588** — Highest eignevalue from Burdex matrix weighted by polarizabilities order-1<br>**D674** — Number of group total hydroxyl groups<br>**D746** — Number of group H attached to C0(sp3) no X attached to next C<br>**NPR1** — Normalized Principal Moment of Inertia 1<br>**SpherocityIndex** — An anisometry descriptor, it varies from zero for flat molecules, to one for totally spherical molecules<br>**PBF** — Plane of best fit<br><br>[Descriptor selection]<br>Feature selection using<br>the “Best First” approach and the “CfsSubset” evaluator<br><br>[Algorithm and descriptor generation]<br>RDKit v2024.3.6, Chem package<br>Enalos+ Mold2 KNIME node<br><br>[Software name and version for descriptor and algorithm generation]<br>RDKit v2024.3.6, Chem package<br>Enalos+ Mold2 KNIME node<br><br>[Chemicals/ Descriptors ratio]<br>3724 compounds/26 descriptors

Computational detail of data mining operation

**3.1**	**Numerical Operations**	
**3.2**	**Software tool**	Isalos Analytics Platform
**3.3**	**Margin Of Error**	[Statistics for goodness-of-fit (validation set)]<br>MCC 0.523<br>Accuracy 0.770<br>Sensitivity/Recall 0.815<br>Specificity 0.705<br>Precision 0.801<br><br>[Robustness – Statistics obtained by Y-scrambling]<br>Y-Randomization / Accuracy / MCC<br># 1 / 0.514 / -0.003<br># 2 / 0.538 / 0.051

1.1		# 3 0.494 -0.048<br># 4 0.517 0.004<br># 5 0.512 -0.007<br><br>[Predictivity – Statistics obtained by external validation]<br>MCC 0.607<br>Accuracy 0.805<br>Sensitivity/Recall 0.817<br>Specificity 0.791<br>Precision 0.828

Model 10

Consensus

Aspect of the User Case/System to be Simulated		
1.1	**Aspect of the User Case to be simulated**	[Endpoint]<br>Ames Mutagenicity<br><br>[Comment on endpoint]<br>Categorical endpoint, each compound is labelled (TDC database) as either “1” (mutagen) or “0” (non-mutagen)<br><br>[Endpoint units]<br>NA
1.2	**Material**	Small organic molecules
1.3	**Geometry**	
1.4	**Time Lapse**	
1.5	**Manufacturing process or in service conditions**	
1.6	**Publication on this data**	

The Data-based Model		
2.0	**Equation type and name**	[Explicit Algorithm]<br>Each model casts a "vote" for a class, and the class with the highest number of votes is selected as the final prediction.<br><br>[Data availability]<br>ChemPharos DB<br>(https://db.chempharos.eu/datasets/Datasets.zul?datasetID=ds18),

		TDC (https://tdcommons.ai/single_pred_tasks/tox/#ames-mutagenicity)	
**2.1**	**Database and type**	ChemPharos DB (https://db.chempharos.eu/datasets/Datasets.zul?datasetID=ds18), TDC (https://tdcommons.ai/single_pred_tasks/tox/#ames-mutagenicity)	
**2.2**	**Equation**	Hypothesis	[Type of model]<br>Majority vote
		Physical quantities	

Computational detail of data mining operation		
**3.1**	**Numerical Operations**	
**3.2**	**Software tool**	Python
**3.3**	**Margin Of Error**	[Predictivity – Statistics obtained by external validation]

MCC	0.648
Accuracy	0.826
Sensitivity/Recall	0.855
Specificity	0.791
Precision	0.834

Model 11

Applicability domain (guided-kNN)

Aspect of the User Case/System to be Simulated		
**1.1**	**Aspect of the User Case to be simulated**	To assess the reliability of the predictions of the guided-kNN model, an applicability domain (AD) methodology is employed aiming to define the models' limitations based on the training compounds.
**1.2**	**Material**	Small organic molecules
**1.3**	**Geometry**	
**1.4**	**Time Lapse**	
**1.5**	**Manufacturing process or in service conditions**	
**1.6**	**Publication on this data**	

The Data-based Model

**2.0**	**Equation type and name**	[Description of the applicability domain of the model]<br>A similarity-based AD is established. In this case whether a query compound is located inside or outside the model's AD limits is decided by comparing its similarity to the training compounds. If this similarity is equal or larger than a predefined S_{cutoff} value with at least N_{min} training compounds, then the query compound is considered inside the AD.<br><br>[Method used to assess the applicability domain]<br>$AD_{fingerprint}\{N_{min}, S_{cutoff}\}$<br><br>[Software name and version for applicability domain assessment]<br>Python<br><br>[Limits of applicability]<br>The N_{min} value is selected to be equal to the number of neighbours used within the guided-kNN (k=9), to ensure that the training neighbours contributing to the prediction are sufficiently similar to the query compound, thus aligning the AD with the guided-kNN's local prediction mechanism. The S_{cutoff} value is selected as the 75th percentile of the distribution of Tanimoto similarities within the training compounds.
**2.1**	**Database and type**	
**2.2**	**Equation**	Hypothesis: [Type of model]<br>Similarity-based<br>Physical quantities: Molecular fingerprints

Computational detail of data mining operation		
**3.1**	**Numerical Operations**	
**3.2**	**Software tool**	RDKit v2024.3.6, Chem package
**3.3**	**Margin Of Error**	

Model 12

Applicability domain (XGBoost)

Aspect of the User Case/System to be Simulated		
**1.1**	**Aspect of the User Case to be simulated**	To assess the reliability of the predictions of the XGBoost model, an applicability domain (AD) methodology is employed aiming to define the models' limitations based on the training compounds.
**1.2**	**Material**	Small organic molecules
**1.3**	**Geometry**	
**1.4**	**Time Lapse**	
**1.5**	**Manufacturing process or in service conditions**	
**1.6**	**Publication on this data**	

The Data-based Model		
**2.0**	**Equation type and name**	[Description of the applicability domain of the model]<br>The applicability domain is defined by fixed boundaries, the APD threshold, calculated by considering Euclidean distances between all molecules in the training set. The distance of a query compound to its nearest neighbour in the training set is compared to the predefined applicability domain threshold. If the distance is beyond this threshold, then the prediction is considered unreliable.<br><br>[Method used to assess the applicability domain]<br>The distance of each test compound to each nearest neighbour of the training compounds is compared to a predefined threshold, $AD = \langle d \rangle + Z\sigma$; if this distance is lower than the threshold then it falls within the AD of the model and its mutagenicity prediction can be considered reliable. To define the AD threshold, Euclidean distances between all training compounds are calculated, as well as the mean value of these distances (D_{av}). In a next step the new

<table>
<tr><td></td><td></td><td colspan="2">average value $\langle d \rangle$ and standard deviation σ of the distances included in the subset of training compounds, which have lower distance than D_{av}, are calculated. Z, is an empirical cut-off value, the default value is equal to 0.5.

[Software name and version for applicability domain assessment]
Isalos Analytics Platform: “Domain – APD” function

[Limits of applicability] APD threshold = 5.421</td></tr>
<tr><td>2.1</td><td>Database and type</td><td colspan="2"></td></tr>
<tr><td rowspan="2">2.2</td><td rowspan="2">Equation</td><td>Hypothesis</td><td>[Type of model]
Distance based</td></tr>
<tr><td>Physical quantities</td><td>[Descriptors in the model]
<table>
<tr><td>D123</td><td>Average of molecular weight</td></tr>
<tr><td>D173</td><td>Mohar order-2 index</td></tr>
<tr><td>D186</td><td>Average vertex distance connectivity index</td></tr>
<tr><td>D279</td><td>Total information content order-4 index</td></tr>
<tr><td>D282</td><td>Structural information content order-1 index</td></tr>
<tr><td>D287</td><td>Complementary information content order-0 index</td></tr>
<tr><td>D297</td><td>Bond information content order-4 index</td></tr>
<tr><td>D349</td><td>Molecular topological path index of order 09</td></tr>
<tr><td>D357</td><td>Molecular topological multiple path index of order 09</td></tr>
<tr><td>D358</td><td>Molecular topological multiple path index of order 10</td></tr>
<tr><td>D366</td><td>Sum of topological distance between the vertices N and N</td></tr>
<tr><td>D367</td><td>Sum of topological distance between the vertices N and P</td></tr>
<tr><td>D413</td><td>Walk-returning count order-5</td></tr>
<tr><td>D477</td><td>Geary topological structure autocorrelation length-7 weighted by atomic polarizabilities</td></tr>
</table></td></tr>
</table>

			**D478**	Geary topological structure autocorrelation length-8 weighted by atomic polarizabilities
			**D532**	Lowest eigenvalue from Burdex matrix weighted by masses order-1
			**D533**	Lowest eigenvalue from Burdex matrix weighted by masses order-2
			**D534**	Lowest eigenvalue from Burdex matrix weighted by masses order-3
			**D535**	Lowest eigenvalue from Burdex matrix weighted by masses order-4
			**D564**	Highest eignevalue from Burdex matrix weighted by masses order-1
			**D588**	Highest eignevalue from Burdex matrix weighted by polarizabilities order-1
			**D674**	Number of group total hydroxyl groups
			**D746**	Number of group H attached to C0(sp3) no X attached to next C
			**NPR1**	Normalized Principal Moment of Inertia 1
			**SpherocityIndex**	An anisometry descriptor, it varies from zero for flat molecules, to one for totally spherical molecules
			**PBF**	Plane of best fit

Computational detail of data mining operation		
**3.1**	**Numerical Operations**	
**3.2**	**Software tool**	Isalos Analytics Platform
**3.3**	**Margin Of Error**	

Model 13

Reliability assessment

Aspect of the User Case/System to be Simulated		
**1.1**	**Aspect of the User Case to be simulated**	We consider that the AD of the two models is well-defined covering the physicochemical and structural domain and thus, can cover the reliability of the predictions of the DL

		model as well, considering that the same training compounds are used in all three methodologies. To increase confidence in the consensus predictions, the outcome of the two AD methods (whether a prediction lies within the respective AD limits) is combined and an overall decision on the reliability of the predictions is made.
**1.2**	**Material**	Small organic molecules
**1.3**	**Geometry**	
**1.4**	**Time Lapse**	
**1.5**	**Manufacturing process or in service conditions**	
**1.6**	**Publication on this data**	

**The Data-based Model**			
**2.0**	**Equation type and name**	[Explicit Algorithm]<br>The degree of reliability for each prediction is characterized as “low” (the query compound falls outside both AD limits), “moderate” (only one of the two AD limits is met), and “high” (the query compound falls within both AD limits).	
**2.1**	**Database and type**		
**2.2**	**Equation**	Hypothesis	[Type of model]<br>Voting scheme
		Physical quantities	

**Computational detail of data mining operation**		
**3.1**	**Numerical Operations**	
**3.2**	**Software tool**	Python
**3.3**	**Margin Of Error**	
