# Supplementary material for: From QSAR to deep learning: an interpretable comprehensive pipeline with a read-across approach for mutagenicity prediction via the Enalos Cloud Platform
Source: RSC Adv. 2026 Sep 9;16(43):53776–805. doi: 10.1039/d6ra05786a (PMC13557173; doi:10.1039/d6ra05786a)
Supplement: Supplementary file 2 [file RA-016-D6RA05786A-s002.pdf]

Supplementary Information (SI) for RSC Advances.
This journal is © The Royal Society of Chemistry 2026

From QSAR to Deep Learning: An interpretable comprehensive pipeline with a read-across approach for mutagenicity prediction via the Enalos Cloud Platform

Dimitra-Danai Varsou[1,2], Andreas Tsoumanis[2,3], Eleonora Marta Longhin[4], Maria Dusinska[4], Dimitris G. Mintis[2,3], Panagiotis D. Kolokathis[1,2], Konstantinos D. Papavasileiou[1,2], Iseult Lynch[5], Georgia Melagraki[6], Antreas Afantitis[1,2,3,7]

[1]NovaMechanics MIKE, Piraeus 18545, Greece
[2]Entelos Institute, Nicosia 2102, Cyprus
[3]NovaMechanics Ltd, Nicosia 1070, Cyprus
[4]Health Effects Laboratory, Department of Environmental Chemistry and Health Effects, NILU - Norwegian Institute for Air Research, Kjeller, Norway
[5]School of Geography, Earth and Environmental Sciences, University of Birmingham, B15 2TT Birmingham, UK
[6]Division of Physical Sciences and Applications, Hellenic Military Academy, 16673, Vari, Greece
[7]Department of Pharmacy, Frederick University, Nicosia 1036, Cyprus

Supporting Information

QMRF reports

QMRF report 1: guided-kNN

	Element	Explanation
**1.**	**QSAR identifier**	
1.1.	QSAR identifier (title)	Pólis - Classification of chemicals mutagenicity (Ames positive/negative) based on the guided-kNN methodology
1.2	Other related models	Xu, C., Cheng, F., Chen, L., Du, Z., Li, W., Liu, G., ... & Tang, Y. (2012). In silico prediction of chemical Ames mutagenicity. *Journal of chemical information and modeling*, 52(11), 2840-2847. - https://doi.org/10.1021/ci300400a<br><br>Other models based on this dataset can be found in the TDC database: https://tdcommons.ai/benchmark/admet_group/21ames/
1.3.	Software coding the model	Python 3.11.5<br>RDKit v2024.3.6<br>pandas v2.1.4<br>numpy v1.24.4<br>scikit-learn v1.4.1<br>Link to the web service:<br>https://www.enaloscloud.novamechanics.com/insight/polis/
**2.**	**General information**	

2.0	Abstract	A machine learning model for the classification of chemicals as “mutagens”/Ames positive or “non-mutagens”/Ames negative based on similarities, 2D and 3D descriptors. The relevant paper describing this model is in preparation.
2.1.	Date of QMRF	March 3, 2026
2.2.	QMRF author(s) and contact details	Dimitra-Danai Varsou, varsou@novamechanics.com<br>Andreas Tsoumanis, tsoumanis@novamechanics.com<br>Georgia Melagraki, georgiamelagraki@gmail.com<br>Antreas Afantitis, afantitis@novamechanics.com
2.3.	Date of QMRF update(s)	-
2.4.	QMRF update(s)	-
2.5.	Model developer(s) and contact details	Dimitra-Danai Varsou, varsou@novamechanics.com<br>Andreas Tsoumanis, tsoumanis@novamechanics.com<br>Georgia Melagraki, georgiamelagraki@gmail.com<br>Antreas Afantitis, afantitis@novamechanics.com
2.6.	Date of model development and/or publication	2025
2.7.	Reference(s) to main scientific papers and/or software package	Paper in preparation
2.8.	Availability of information about the model	The model is proprietary: the source code is confidential; however, the description of the modelling workflow is presented in the original research article, training and validation sets are available as supplementary information of the original research article and the model is implemented as a public web service.
2.9.	Availability of another QMRF for exactly the same model	-
**3**	**Defining the endpoint - OECD Principle 1: “A DEFINED ENDPOINT"**	**PRINCIPLE 1: “A DEFINED ENDPOINT". ENDPOINT refers to any physicochemical, biological, or environmental property/activity/effect that can be measured and therefore modelled. The intent of PRINCIPLE 1 (a (Q)SAR should be associated with a defined endpoint) is to ensure clarity in the endpoint being predicted by a given model, since a given endpoint could be determined by different experimental protocols and under different experimental conditions. It is therefore important to identify the experimental system and test conditions that is being modelled by the (Q)SAR.**
3.1.	Species	Bacteria (*Salmonella typhimurium*, *Escherichia coli*)
3.2.	Endpoint	Endpoint: detection of gene (point) mutations<br>The experimental protocol for the Ames bacterial reverse mutation test is covered by the OECD Test Guideline (TG) 471
3.3	Comment on endpoint	Exposure to amino-acid requiring strains (histidine-*Salmonella typhimurium* or tryptophan-*Escherichia coli*, respectively)
3.4.	Endpoint units	Classification: Ames positive or negative. Positive results from the bacterial reverse mutation test indicate that a substance induces point mutations by base substitutions or frameshifts in the genome of either *Salmonella typhimurium* and/or *Escherichia coli*.
3.5.	Dependent variable	Ames mutagenicity class: “Mutagenic” (Ames positive) or “Non-mutagenic” (Ames negative)
3.6.	Experimental protocol	See OECD Test Guideline (TG) 471. Data was collected and curated as described in Xu, C., Cheng, F., Chen, L., Du, Z., Li, W., Liu, G., ... & Tang, Y. (2012). In silico prediction of chemical Ames mutagenicity. *Journal of chemical information and modeling*, 52(11), 2840-2847. - https://doi.org/10.1021/ci300400a

<table>
<tr><td>3.7.</td><td>Endpoint data quality and variability</td><td>Details on the initial data collection and curation can be found in the work of Xu, C., Cheng, F., Chen, L., Du, Z., Li, W., Liu, G., ... & Tang, Y. (2012). In silico prediction of chemical Ames mutagenicity. Journal of chemical information and modeling, 52(11), 2840-2847. - https://doi.org/10.1021/ci300400a

Data are retrieved from the Therapeutics Data Commons (TDC) platform: https://tdcommons.ai/single_pred_tasks/tox/#ames-mutagenicity

from tdc.single_pred import Tox
data = Tox(name = 'AMES')
df = data.get_data()

An additional filtering of the chemical compounds is performed so that the 3D descriptors (see §4.5) correspond as closely as possible to the input compounds, excluding those with ambiguous stereochemistry. The RDKit EnumerateStereoisomers module is used to search for possible stereoisomers for each one of the compounds of the training, validation and test sets. Compounds that yield multiple stereoisomers due to unassigned stereocenters, are filtered out.

Classes distribution between sets after data curation:
<table>
<tr><th>Subset</th><th>All</th><th>Mutagens (Ames positive)</th><th>Non-mutagens (Ames negative)</th></tr>
<tr><td>Training</td><td>3724</td><td>2090</td><td>1634</td></tr>
<tr><td>Validation</td><td>549</td><td>325</td><td>224</td></tr>
<tr><td>Test</td><td>1089</td><td>601</td><td>488</td></tr>
<tr><td>Total</td><td>5362</td><td>3016</td><td>2346</td></tr>
</table></td></tr>
<tr><td>4</td><td>Defining the algorithm - OECD Principle 2: “AN UNAMBIGUOUS ALGORITHM”</td><td>PRINCIPLE 2: “AN UNAMBIGUOUS ALGORITHM”. The (Q)SAR estimate of an endpoint is the result of applying an ALGORITHM to a set of structural parameters which describe the chemical structure. The intent of PRINCIPLE 2 (a (Q)SAR should be associated with an unambiguous algorithm) is to ensure transparency in the model algorithm that generates predictions of an endpoint from information on chemical structure and/or physicochemical properties. In this context, algorithm refers to any mathematical equation, decision rule or output approach.</td></tr>
<tr><td>4.1.</td><td>Type of model</td><td>Read-across model, similarity and kNN based</td></tr>
<tr><td>4.2.</td><td>Explicit algorithm</td><td>1. Each molecule is encoded with a chemical fingerprint and Tanimoto similarity is calculated between query molecules and training set molecules.
2. The k neighbours are selected based on their Tanimoto similarity ranking (k=9).
3. The class prediction for the query molecule is the weighted majority vote of the k neighbouring molecules. Weights are proportional to the inverse distances between these neighbours, which are calculated using their molecular descriptors.</td></tr>
</table>

4.3.	Descriptors in the model	• SlogP_VSA8: A MOE-type descriptor that sums the van der Waals surface area (VSA) contributions of atoms assigned to bin 8 based on their atomic contributions to the logP (expressing hydrophobicity)<br>• fr_nitro: Number of nitro groups<br>• NumAromaticRings: Number of aromatic rings in the molecule<br>• HallKierAlpha: The Hall-Kier alpha value for a molecule<br>• RingCount: Total number of rings in the molecule<br>• fr_bicyclic: Number of bicyclic rings<br>• NumAromaticCarbocycles: Number of aromatic rings containing only carbon atoms.<br>• fr_benzene: Number of benzene rings<br>• BertzCT: A topological index meant to quantify complexity of molecules<br>• fr_nitro_arom_nonortho: Number of non-ortho nitro benzene ring substituents<br>• fr_nitro_arom: Number of nitro benzene ring substituents<br>• PBF: Plane of best fit<br>• PEOE_VSA2: A MOE-type descriptor that sums the van der Waals surface area (VSA) contributions of atoms assigned to bin 2 based on their partial equalization of orbital electronegativity (PEOE) charge values<br>• AvgIpc: Average information content per atom<br>• fr_NH0: Number of Tertiary amines<br>• SMR_VSA5: A MOE-type descriptor that sums the van der Waals surface area (VSA) contributions of atoms assigned to bin 5 based on their atomic contributions to the MR (molecular refractivity)<br>• SlogP_VSA10: A MOE-type descriptor that sums the van der Waals surface area (VSA) contributions of atoms assigned to bin 10 based on their atomic contributions to the logP (expressing hydrophobicity)<br>• SpherocityIndex: An anisometry descriptor, it varies from zero for flat molecules, to one for totally spherical molecules<br>• SMR_VSA7: A MOE-type descriptor that sums the van der Waals surface area (VSA) contributions of atoms assigned to bin 7 based on their atomic contributions to the MR (molecular refractivity)<br>• SlogP_VSA5: A MOE-type descriptor that sums the van der Waals surface area (VSA) contributions of atoms assigned to bin 5 based on their atomic contributions to the logP (expressing hydrophobicity)<br>• SMR_VSA10: A MOE-type descriptor that sums the van der Waals surface area (VSA) contributions of atoms assigned to bin 10 based on their atomic contributions to the MR (molecular refractivity)<br>• SlogP_VSA4: A MOE-type descriptor that sums the van der Waals surface area (VSA) contributions of atoms assigned to bin 4 based on their atomic contributions to the logP (expressing hydrophobicity)<br>• VSA_EState8: A MOE-type descriptor that sums the electrotopological state (EState) indices of atoms assigned to bin 8 based on their van der Waals surface area (VSA) contributions<br>• FractionCSP3: Fraction of sp^3-hybridized carbon atoms<br>• VSA_EState6: A MOE-type descriptor that sums the electrotopological state (EState) indices of atoms assigned to bin 6 based on their van der Waals surface area (VSA) contributions

		• PEOE_VSA13: A MOE-type descriptor that sums the van der Waals surface area (VSA) contributions of atoms assigned to bin 13 based on their partial equalization of orbital electronegativity (PEOE) charge values<br>• Qed: Quantitative Estimate of Drug-likeness<br>• FpDensityMorgan3: Fingerprint density for Morgan fingerprints (radius=3)<br>• SlogP_VSA6: A MOE-type descriptor that sums the van der Waals surface area (VSA) contributions of atoms assigned to bin 6 based on their atomic contributions to the logP (expressing hydrophobicity)<br>• NOCount: Number of Nitrogen and Oxygen atoms<br>• VSA_EState3: A MOE-type descriptor that sums the electrotopological state (EState) indices of atoms assigned to bin 3 based on their van der Waals surface area (VSA) contributions<br>• TPSA: Topological polar surface area<br>• VSA_EState4: A MOE-type descriptor that sums the electrotopological state (EState) indices of atoms assigned to bin 4 based on their van der Waals surface area (VSA) contributions<br>• NumHAcceptors: Number of Hydrogen Bond Acceptors<br>• fr_unbrch_alkane: Number of unbranched alkanes of at least 4 members (excludes halogenated alkanes)<br>• Chi4n: A valence molecular connectivity index calculated based on Rev. Comp. Chem., vol 2, 367-422 (1991).<br>• PEOE_VSA8: A MOE-type descriptor that sums the van der Waals surface area (VSA) contributions of atoms assigned to bin 8 based on their partial equalization of orbital electronegativity (PEOE) charge values<br>• VSA_EState7: A MOE-type descriptor that sums the electrotopological state (EState) indices of atoms assigned to bin 7 based on their van der Waals surface area (VSA) contributions<br>• EState_VSA10: A MOE-type descriptor that sums the van der Waals surface area (VSA) contributions of atoms assigned to bin 10 based on their electrotopological state (EState) index<br>• fr_COO2: Number of carboxylic acids<br>• Chi1: A molecular connectivity index calculated from equations (1), (11) and (12) of Rev. Comp. Chem. vol 2, 367-422, (1991)<br>• fr_ArN: Number of aromatic nitrogens<br>• BalabanJ: A chemical distance-based topological index from Chem. Phys. Lett. vol 89, 399-404 (1982).<br>• VSA_EState2: A MOE-type descriptor that sums the electrotopological state (EState) indices of atoms assigned to bin 2 based on their van der Waals surface area (VSA) contributions<br>• NumRotatableBonds: Number of Rotatable Bonds<br>• HeavyAtomCount: Number of heavy atoms of a molecule<br>• SlogP_VSA3: A MOE-type descriptor that sums the van der Waals surface area (VSA) contributions of atoms assigned to bin 3 based on their atomic contributions to the logP (expressing hydrophobicity)<br>• Chi3n: A valence molecular connectivity index calculated based on Rev. Comp. Chem., vol 2, 367-422 (1991).<br>• NumHeteroatoms: Number of Heteroatoms<br>• fr_aniline: Number of anilines

4.4.	Descriptor selection	Initial pool of descriptors: 221 RDKit descriptors<br>These descriptors are next standardized with z-score (Gaussian) transformation<br>Selection methods: Mutual information filtering & KBest feature selection algorithm
4.5.	Algorithm and descriptor generation	RDKit software calculates 210 descriptors that encode physicochemical, topological, and structural properties of compounds, again based on their 2D structure. Additionally, 11 descriptors based on the 3D molecular structure (3D conformers) are calculated using RDKit.
4.6.	Software name and version for descriptor generation	RDKit v2024.3.6, Chem package
4.7.	Chemicals/Descriptors ratio	Chemicals/ Descriptors ratio: 74.5
**5**	**Defining the applicability domain - OECD Principle 3: “A DEFINED DOMAIN OF APPLICABILITY”**	**PRINCIPLE 3: “A DEFINED DOMAIN OF APPLICABILITY”. APPLICABILITY DOMAIN refers to the response and chemical structure space in which the model makes predictions with a given reliability. Ideally the applicability domain should express the structural, physicochemical and response space of the model. The CHEMICAL STRUCTURE (x variable) space can be expressed by information on physicochemical properties and/or structural fragments. The RESPONSE (y variable) can be any physicochemical, biological or environmental effect that is being predicted. According to PRINCIPLE 3 a (Q)SAR should be associated with a defined domain of applicability. Section 5 can be repeated (e.g., 5.a, 5.b, 5.c, etc) as many times as necessary if more than one method has been used to assess the applicability domain.**
5.1.	Description of the applicability domain of the model	A similarity-based AD is established. In this case whether a query compound is located inside or outside the model’s AD limits is decided by comparing its similarity to the training compounds. If this similarity is equal or larger than a predefined S_{cutoff} value with at least N_{min} training compounds, then the query compound is considered inside the AD. This is expressed as: $$AD_{fingerprint}\{N_{min}, S_{cutoff}\}$$
5.2.	Method used to assess the applicability domain	The N_{min} value is selected to be equal to the number of neighbours used within the guided-kNN (k=9), to ensure that the training neighbours contributing to the prediction are sufficiently similar to the query compound, thus aligning the AD with the guided-kNN’s local prediction mechanism. The S_{cutoff} value is selected as the 75th percentile of the distribution of Tanimoto similarities within the training compounds.
5.3.	Software name and version for applicability domain assessment	Python 3.11.5<br>pandas v2.1.4<br>numpy v1.24.4
5.4.	Limits of applicability	Only the top 25% of the most similar training compounds are considered in the applicability domain definition. This value represents a rather strict threshold, but it ensures that only highly similar compounds are included within the AD.
**6**	**Defining goodness-of-fit and robustness (internal validation) – OECD Principle 4: “APPROPRIATE MEASURES OF GOODNESS-OF-FIT, ROBUSTENESS AND PREDICTIVITY”**	**PRINCIPLE 4: “APPROPRIATE MEASURES OF GOODNESS-OF-FIT, ROBUSTENESS AND PREDICTIVITY”. PRINCIPLE 4 expresses the need to perform validation to establish the performance of the model. GOODNESS-OF-FIT and ROBUSTNESS refer to the internal model performance.**

6.1.	Availability of the training set	Available at the ChemPharos database:<br>https://db.chempharos.eu/datasets/Datasets.zul?datasetID=ds18<br><br>Training compounds are labelled accordingly, see column "Subset" column (ALY).
6.2.	Available information for the training set	Chemical names (common names and IUPAC names)<br>SMILES<br>Molecular formulas<br>InChI codes<br>InChIKeys<br>PubChem URLs<br>CIDs
6.3.	Data for each descriptor variable for the training set	Available at the ChemPharos database:<br>https://db.chempharos.eu/datasets/Datasets.zul?datasetID=ds18<br><br>For each compound, 210 2D RDKit (columns ADA-ALB), and 11 3D RDKit descriptors (columns ALC-ALM) are available. The subset of descriptors used for modelling are presented in §4.3.
6.4.	Data for the dependent variable for the training set	Available at the ChemPharos database:<br>https://db.chempharos.eu/datasets/Datasets.zul?datasetID=ds18<br><br>Each compound is labelled ("Y"-column C) as either mutagenic/Ames positive (class 1) or non-mutagenic/Ames negative (class 0).
6.5.	Other information about the training set	-
6.6.	Pre-processing of data before modelling	Descriptors are standardized prior to feature selection with z-score (Gaussian) transformation. This linear transformation ensures that normalized values present a mean value equal to 0 and standard deviation equal to 1.
6.7.	Statistics for goodness-of-fit	<table><tr><td>Statistics</td><td>Validation set</td></tr><tr><td>MCC</td><td>0.602</td></tr><tr><td>Accuracy</td><td>0.807</td></tr><tr><td>Sensitivity/Recall</td><td>0.828</td></tr><tr><td>Specificity</td><td>0.777</td></tr><tr><td>Precision</td><td>0.843</td></tr></table>
6.8.	Robustness - Statistics obtained by leave-one-out cross-validation	<table><tr><td>Statistics</td><td>Training set (LOO)</td></tr><tr><td>MCC</td><td>0.569</td></tr><tr><td>Accuracy</td><td>0.788</td></tr><tr><td>Sensitivity/Recall</td><td>0.811</td></tr><tr><td>Specificity</td><td>0.758</td></tr><tr><td>Precision</td><td>0.811</td></tr></table>
6.9.	Robustness - Statistics obtained by leave-many-out cross-validation	-
6.10.	Robustness - Statistics obtained by Y-scrambling	Statistics (test set) for 5 iterations with scrambled endpoint values:<br><table><tr><td>Y-Randomization</td><td>Accuracy</td><td>MCC</td></tr><tr><td># 1</td><td>0.495</td><td>-0.038</td></tr><tr><td># 2</td><td>0.522</td><td>0.018</td></tr><tr><td># 3</td><td>0.472</td><td>-0.087</td></tr><tr><td># 4</td><td>0.516</td><td>0.011</td></tr></table>

<table>
<tr><td></td><td></td><td><table><tr><td># 5</td><td>0.496</td><td>-0.035</td></tr></table></td></tr>
<tr><td>6.11.</td><td>Robustness - Statistics obtained by bootstrap</td><td>-</td></tr>
<tr><td>6.12.</td><td>Robustness - Statistics obtained by other methods</td><td>-</td></tr>
<tr><td>7</td><td>Defining predictivity (external validation) – OECD Principle 4: “APPROPRIATE MEASURES OF GOODNESS-OF-FIT, ROBUSTENESS AND PREDICTIVITY”</td><td>PRINCIPLE 4: “APPROPRIATE MEASURES OF GOODNESS-OF-FIT, ROBUSTENESS AND PREDICTIVITY”. PRINCIPLE 4 expresses the need to perform validation to establish the performance of the model. PREDICTIVITY refers to the external model validation. Section 7 can be repeated (e.g., 7.a, 7.b, 7.c, etc) as many times as necessary if more validation studies need to be reported in the QMRF.</td></tr>
<tr><td>7.1.</td><td>Availability of the external validation set</td><td>Available at the ChemPharos database:
https://db.chempharos.eu/datasets/Datasets.zul?datasetID=ds18

Testing compounds are labelled accordingly, see column “Subset” column (ALY).</td></tr>
<tr><td>7.2.</td><td>Available information for the external validation set</td><td>Chemical names (common names and IUPAC names)
SMILES
Molecular formulas
InChI codes
InChIKeys
PubChem URLs
CIDs</td></tr>
<tr><td>7.3.</td><td>Data for each descriptor variable for the external validation set</td><td>Available at the ChemPharos database:
https://db.chempharos.eu/datasets/Datasets.zul?datasetID=ds18

For each compound, 210 2D RDKit (columns ADA-ALB), and 11 3D RDKit descriptors (columns ALC-ALM) are available. The subset of descriptors used in the model are presented in §4.3.</td></tr>
<tr><td>7.4.</td><td>Data for the dependent variable for the external validation set</td><td>Available at the ChemPharos database:
https://db.chempharos.eu/datasets/Datasets.zul?datasetID=ds18

Each compound is labelled (“Y”-column C) as either mutagenic/Ames positive (class 1) or non-mutagenic/Ames negative (class 0).</td></tr>
<tr><td>7.5.</td><td>Other information about the external validation set</td><td>-</td></tr>
<tr><td>7.6.</td><td>Experimental design of test set</td><td>TDC’s protocol is followed for random data splitting into training (70%), validation (10%) and test (20%) subsets.

from tdc.single_pred import Tox
data = Tox(name = 'AMES')
df = data.get_data()
split = data.get_split(method = 'random')</td></tr>
<tr><td>7.7.</td><td>Predictivity - Statistics obtained by external validation</td><td><table><tr><th>Statistics</th><th>Test set</th></tr><tr><td>MCC</td><td>0.599</td></tr><tr><td>Accuracy</td><td>0.802</td></tr><tr><td>Sensitivity/Recall</td><td>0.845</td></tr><tr><td>Specificity</td><td>0.749</td></tr><tr><td>Precision</td><td>0.806</td></tr></table></td></tr>
</table>

7.8.	Predictivity - Assessment of the external validation set	98.8% of the test set compounds fall inside the applicability domain limits of the model.
7.9.	Comments on the external validation of the model	The test set was normalized using the Gaussian normalization function, which was also applied on the training set.
**8**	**Providing a mechanistic interpretation - OECD Principle 5: "A MECHANISTIC INTERPRETATION, IF POSSIBLE"**	**PRINCIPLE 5: "A MECHANISTIC INTERPRETATION, IF POSSIBLE". According to PRINCIPLE 5, a (Q)SAR should be associated with a mechanistic interpretation, if possible.**
8.1.	Mechanistic basis of the model	Descriptors are described in §4.3.
8.2.	A priori or a posteriori mechanistic interpretation	A posteriori mechanistic interpretation. Among the top-ranked RDKit descriptors, aromaticity-related structural features (number of aromatic rings, aromatic carbocycles, benzene rings) and nitro group-related features (number of nitro groups) are identified as key descriptors. Both aromatic (especially polycyclic aromatic compounds) and nitroaromatic compounds have been associated with mutagenic activity. The planar structure of aromatic rings can intercalate between DNA base pairs, causing structural distortions that can lead to mutations whereas, the nitro group can undergo metabolic activation to form reactive intermediates that interact with DNA. It should be also noted that the nitro aromatic group substituents may also act as detoxifying substructures of nitroaromatics depending on their position. For instance, the introduction of bulky alkyl substituents ortho to the nitro functional group in 4-nitrobiphenyl eliminates mutagenicity compared to the parent compound. This reduction in mutagenic potential is associated with steric hindrance, which forces the nitro group out of the aromatic plane and disrupts its reactivity, that could otherwise lead to DNA adducts formation. These findings may be generalizable to other nitroaromatic compounds with similar substitution patterns, nonetheless further assessment of the different substituent effects could be addressed in future studies. In our model, the number of ortho nitro aromatic group substituents can be derived by the two selected descriptors (number of nitro benzene ring substituents and of non-ortho nitro benzene ring substituents), thus overall, the guided-kNN model captures competing effects as it accounts for structural features that both promote and mitigate mutagenicity. Other physicochemical features such as hydrophobicity-based MOE descriptors (SlogP_VSA8, SlogP_VSA10, SlogP_VSA5) suggested that hydrophobicity (often denoted as logP and commonly derived as a key descriptor for mutagenicity prediction) influences cellular uptake, as it controls the penetration of molecules to cell membranes and interacts with intracellular components, increasing the potential of mutagenic interactions. Molar refractivity-based descriptors (SMR_VSA5, SMR_VSA7) encode molecular polarizability, and can serve as a measure of the interactions between biological receptors and substrates. Other highly ranked descriptors, such as spherocity, which measures molecular anisotropy, are not commonly associated with mutagenicity in the literature. Nonetheless, descriptors like Plane of Best Fit (PBF) and SpherocityIndex effectively capture molecular shape and planarity (key structural features of known mutagenic polycyclic aromatic scaffolds such as acridine, phenanthrene, pyrene, and quinoxaline). In brief, spherocity values close to zero correspond to flat molecules, whereas values near one describe spherical ones. Finally, low PBF values suggest molecules are nearly planar, whereas higher PBF values reflect greater three-dimensionality, with atoms more dispersed out of a single plane.
8.3.	Other information about the mechanistic interpretation	-

9	**Miscellaneous information**	
9.1.	Comments	-
9.2.	Bibliography	-
9.3	Supporting information	The curated and enriched dataset is available in the chemPharos database, where the training, validation, and test observations are clearly indicated: https://db.chempharos.eu/datasets/Datasets.zul?datasetID=ds18

QMRF report 2: GCNN

	**Element**	**Explanation**
**1.**	**QSAR identifier**	
1.1.	QSAR identifier (title)	Pólis - Classification of chemicals mutagenicity (Ames positive/negative) based on the GCNN model
1.2	Other related models	Xu, C., Cheng, F., Chen, L., Du, Z., Li, W., Liu, G., ... & Tang, Y. (2012). In silico prediction of chemical Ames mutagenicity. *Journal of chemical information and modeling*, 52(11), 2840-2847. - https://doi.org/10.1021/ci300400a<br><br>Other models based on this dataset can be found in the TDC database: https://tdcommons.ai/benchmark/admet_group/21ames/
1.3.	Software coding the model	deepchem v2.8.1<br>Keras and TensorFlow v2.15.0<br>Link to the web service:<br>https://www.enaloscloud.novamechanics.com/insight/polis/
**2.**	**General information**	
2.0	Abstract	A deep learning model for the classification of chemicals as "mutagens"/Ames positive or "non-mutagens"/Ames negative based on similarities, 2D and 3D descriptors. The relevant paper describing this model is in preparation.
2.1.	Date of QMRF	March 3, 2026
2.2.	QMRF author(s) and contact details	Dimitra-Danai Varsou, varsou@novamechanics.com<br>Andreas Tsoumanis, tsoumanis@novamechanics.com<br>Georgia Melagraki, georgiamelagraki@gmail.com<br>Antreas Afantitis, afantitis@novamechanics.com
2.3.	Date of QMRF update(s)	-
2.4.	QMRF update(s)	-
2.5.	Model developer(s) and contact details	Dimitra-Danai Varsou, varsou@novamechanics.com<br>Andreas Tsoumanis, tsoumanis@novamechanics.com<br>Georgia Melagraki, georgiamelagraki@gmail.com<br>Antreas Afantitis, afantitis@novamechanics.com
2.6.	Date of model development and/or publication	2025
2.7.	Reference(s) to main scientific papers and/or software package	Paper in preparation

<table>
<tr><td>2.8.</td><td>Availability of information about the model</td><td>The model is proprietary: the source code is confidential; however, the description of the modelling workflow is presented in the original research article, training and validation sets are available as supplementary information of the original research article and the model is implemented as a public web service.</td></tr>
<tr><td>2.9.</td><td>Availability of another QMRF for exactly the same model</td><td>-</td></tr>
<tr><td>3</td><td>Defining the endpoint - OECD Principle 1: “A DEFINED ENDPOINT"</td><td>PRINCIPLE 1: “A DEFINED ENDPOINT". ENDPOINT refers to any physicochemical, biological, or environmental property/activity/effect that can be measured and therefore modelled. The intent of PRINCIPLE 1 (a (Q)SAR should be associated with a defined endpoint) is to ensure clarity in the endpoint being predicted by a given model, since a given endpoint could be determined by different experimental protocols and under different experimental conditions. It is therefore important to identify the experimental system and test conditions that is being modelled by the (Q)SAR.</td></tr>
<tr><td>3.1.</td><td>Species</td><td>Bacteria (Salmonella typhimurium, Escherichia coli)</td></tr>
<tr><td>3.2.</td><td>Endpoint</td><td>Endpoint: detection of gene (point) mutations
The experimental protocol for the Ames bacterial reverse mutation test is covered by the OECD Test Guideline (TG) 471</td></tr>
<tr><td>3.3</td><td>Comment on endpoint</td><td>Exposure to amino-acid requiring strains (histidine-Salmonella typhimurium or tryptophan-Escherichia coli, respectively)</td></tr>
<tr><td>3.4.</td><td>Endpoint units</td><td>Classification: Ames positive or negative. Positive results from the bacterial reverse mutation test indicate that a substance induces point mutations by base substitutions or frameshifts in the genome of either Salmonella typhimurium and/or Escherichia coli.</td></tr>
<tr><td>3.5.</td><td>Dependent variable</td><td>Ames mutagenicity class: “Mutagenic” (Ames positive) or “Non-mutagenic” (Ames negative)</td></tr>
<tr><td>3.6.</td><td>Experimental protocol</td><td>See OECD Test Guideline (TG) 471. Data was collected and curated as described in Xu, C., Cheng, F., Chen, L., Du, Z., Li, W., Liu, G., ... & Tang, Y. (2012). In silico prediction of chemical Ames mutagenicity. Journal of chemical information and modeling, 52(11), 2840-2847. - https://doi.org/10.1021/ci300400a</td></tr>
<tr><td>3.7.</td><td>Endpoint data quality and variability</td><td>Details on the initial data collection and curation can be found in the work of Xu, C., Cheng, F., Chen, L., Du, Z., Li, W., Liu, G., ... & Tang, Y. (2012). In silico prediction of chemical Ames mutagenicity. Journal of chemical information and modeling, 52(11), 2840-2847. - https://doi.org/10.1021/ci300400a

Data are retrieved from the Therapeutics Data Commons (TDC) platform: https://tdcommons.ai/single_pred_tasks/tox/#ames-mutagenicity

<pre>from tdc.single_pred import Tox
data = Tox(name = 'AMES')
df = data.get_data()</pre>
Classes distribution between sets:
<table>
<tr><th>Subset</th><th>All</th><th>Mutagens (Ames positive)</th><th>Non-mutagens (Ames negative)</th></tr>
<tr><td>Training</td><td>5076</td><td>2741</td><td>2335</td></tr>
<tr><td>Validation</td><td>726</td><td>415</td><td>311</td></tr>
<tr><td>Test</td><td>1453</td><td>795</td><td>658</td></tr>
<tr><td>Total</td><td>7255</td><td>3951</td><td>3304</td></tr>
</table></td></tr>
</table>

<table>
<tr><td>4</td><td>Defining the algorithm - OECD Principle 2: “AN UNAMBIGUOUS ALGORITHM”</td><td>PRINCIPLE 2: “AN UNAMBIGUOUS ALGORITHM”. The (Q)SAR estimate of an endpoint is the result of applying an ALGORITHM to a set of structural parameters which describe the chemical structure. The intent of PRINCIPLE 2 (a (Q)SAR should be associated with an unambiguous algorithm) is to ensure transparency in the model algorithm that generates predictions of an endpoint from information on chemical structure and/or physicochemical properties. In this context, algorithm refers to any mathematical equation, decision rule or output approach.</td></tr>
<tr><td>4.1.</td><td>Type of model</td><td>Graph Convolutional Neural Networks</td></tr>
<tr><td>4.2.</td><td>Explicit algorithm</td><td>Convolutional neural networks (CNNs) are widely used in processing and modelling images, speech, and time-series data because convolutional layers take advantage of hierarchical patterns and extract high-level features from input data, leading to powerful representational capabilities. Early layers in CNNs detect small-scale local patterns, while deeper layers capture larger, more abstract features. Graph convolutional neural networks (GCNNs) extend CNNs to graph-structured data by leveraging the inherent graph topology. In the context of cheminformatics, GCNNs combine information from neighbouring nodes—bonded atoms in a molecule—in a convolutional manner, allowing for effective learning directly on molecular graphs.

The GCNN model treats each molecule as a graph and processes these graphs through multiple GraphConv layers that implements the graph convolution. Each GraphConv layer is followed by BatchNormalization to stabilize training and Dropout to reduce overfitting. A GraphPool layer then performs max pooling over the feature vectors of atoms in a neighbourhood.
The pooled features of the last GraphConv layers are passed through a fully connected Dense layer with ReLU activation function for further refinement. Normalization and dropout layers are introduced at this stage, as well. A GraphGather layer then aggregates the entire graph’s representation. Finally, a Dense layer reshapes the output (via Reshape) and applies a softmax activation function, yielding probability distributions over the two classes. A probability closer to 1 is assigned to mutagenic compounds and a probability closer to 0 to non-mutagenic ones. The model is trained using the AdamW optimizer -combining adaptive learning rates with weight decay- and a categorical cross-entropy loss to update the network’s weights.

<table>
<tr><td>Hyperparameters</td><td>Optimized hyperparameters</td></tr>
<tr><td>Graph convolution layers</td><td>[64, 64]</td></tr>
<tr><td>Dense layer size</td><td>128</td></tr>
<tr><td>Batch size</td><td>32</td></tr>
<tr><td>Dropout</td><td>0.123</td></tr>
<tr><td>Learning rate</td><td>0.006</td></tr>
<tr><td>Weight decay</td><td>0.001</td></tr>
<tr><td>Activation function</td><td>ReLU</td></tr>
</table></td></tr>
<tr><td>4.3.</td><td>Descriptors in the model</td><td>Compounds are featurized as molecular graphs. No descriptors are used.</td></tr>
<tr><td>4.4.</td><td>Descriptor selection</td><td>-</td></tr>
<tr><td>4.5.</td><td>Algorithm and descriptor generation</td><td>-</td></tr>
</table>

4.6.	Software name and version for descriptor generation	PyTDC v0.3.6<br>deepchem v2.8.1
4.7.	Chemicals/Descriptors ratio	-
**5**	**Defining the applicability domain - OECD Principle 3: "A DEFINED DOMAIN OF APPLICABILITY"**	**PRINCIPLE 3: "A DEFINED DOMAIN OF APPLICABILITY". APPLICABILITY DOMAIN refers to the response and chemical structure space in which the model makes predictions with a given reliability. Ideally the applicability domain should express the structural, physicochemical and response space of the model. The CHEMICAL STRUCTURE (x variable) space can be expressed by information on physicochemical properties and/or structural fragments. The RESPONSE (y variable) can be any physicochemical, biological or environmental effect that is being predicted. According to PRINCIPLE 3 a (Q)SAR should be associated with a defined domain of applicability. Section 5 can be repeated (e.g., 5.a, 5.b, 5.c, etc) as many times as necessary if more than one method has been used to assess the applicability domain.**
5.1.	Description of the applicability domain of the model	-
5.2.	Method used to assess the applicability domain	-
5.3.	Software name and version for applicability domain assessment	-
5.4.	Limits of applicability	-
**6**	**Defining goodness-of-fit and robustness (internal validation) – OECD Principle 4: "APPROPRIATE MEASURES OF GOODNESS-OF-FIT, ROBUSTENESS AND PREDICTIVITY"**	**PRINCIPLE 4: "APPROPRIATE MEASURES OF GOODNESS-OF-FIT, ROBUSTENESS AND PREDICTIVITY". PRINCIPLE 4 expresses the need to perform validation to establish the performance of the model. GOODNESS-OF-FIT and ROBUSTNESS refer to the internal model performance.**
6.1.	Availability of the training set	Data can be directly retrieved from the TDC database:<br>https://tdcommons.ai/single_pred_tasks/tox/#ames-mutagenicity<br><br>`from tdc.single_pred import Tox`<br>`data = Tox(name = 'AMES')`<br>`df = data.get_data()`<br>`split = data.get_split(method = 'random')`<br>`train = split['train']`
6.2.	Available information for the training set	SMILES
6.3.	Data for each descriptor variable for the training set	-
6.4.	Data for the dependent variable for the training set	Each compound is labelled ("Y") as either mutagenic/Ames positive (class 1) or non-mutagenic/Ames negative (class 0).
6.5.	Other information about the training set	-
6.6.	Pre-processing of data before modelling	-
6.7.	Statistics for goodness-of-fit	(see table below)

Statistics	Validation set
ROC-AUC	0.901
MCC	0.656
Accuracy	0.831

<table>
<tr><td></td><td></td><td><table><tr><td>Sensitivity/Recall</td><td>0.841</td></tr><tr><td>Specificity</td><td>0.817</td></tr><tr><td>Precision</td><td>0.860</td></tr></table></td></tr>
<tr><td>6.8.</td><td>Robustness - Statistics obtained by leave-one-out cross-validation</td><td>-</td></tr>
<tr><td>6.9.</td><td>Robustness - Statistics obtained by leave-many-out cross-validation</td><td>-</td></tr>
<tr><td>6.10.</td><td>Robustness - Statistics obtained by Y-scrambling</td><td>Statistics (test set) for 5 iterations with scrambled endpoint values:<table><tr><th>Y-Randomization</th><th>Accuracy</th><th>MCC</th></tr><tr><td># 1</td><td>0.531</td><td>0.064</td></tr><tr><td># 2</td><td>0.480</td><td>-0.030</td></tr><tr><td># 3</td><td>0.539</td><td>0.041</td></tr><tr><td># 4</td><td>0.515</td><td>0.004</td></tr><tr><td># 5</td><td>0.522</td><td>0.036</td></tr></table></td></tr>
<tr><td>6.11.</td><td>Robustness - Statistics obtained by bootstrap</td><td>-</td></tr>
<tr><td>6.12.</td><td>Robustness - Statistics obtained by other methods</td><td>-</td></tr>
<tr><td>7</td><td>Defining predictivity (external validation) – OECD Principle 4: “APPROPRIATE MEASURES OF GOODNESS-OF-FIT, ROBUSTENESS AND PREDICTIVITY”</td><td>PRINCIPLE 4: “APPROPRIATE MEASURES OF GOODNESS-OF-FIT, ROBUSTENESS AND PREDICTIVITY”. PRINCIPLE 4 expresses the need to perform validation to establish the performance of the model. PREDICTIVITY refers to the external model validation. Section 7 can be repeated (e.g., 7.a, 7.b, 7.c, etc) as many times as necessary if more validation studies need to be reported in the QMRF.</td></tr>
<tr><td>7.1.</td><td>Availability of the external validation set</td><td>Data can be directly retrieved from the TDC database:
https://tdcommons.ai/single_pred_tasks/tox/#ames-mutagenicity<pre><code>from tdc.single_pred import Tox
data = Tox(name = 'AMES')
df = data.get_data()
split = data.get_split(method = 'random')
test = split['test']</code></pre></td></tr>
<tr><td>7.2.</td><td>Available information for the external validation set</td><td>SMILES</td></tr>
<tr><td>7.3.</td><td>Data for each descriptor variable for the external validation set</td><td>-</td></tr>
<tr><td>7.4.</td><td>Data for the dependent variable for the external validation set</td><td>Each compound is labelled (“Y”) as either mutagenic/Ames positive (class 1) or non-mutagenic/Ames negative (class 0).</td></tr>
<tr><td>7.5.</td><td>Other information about the external validation set</td><td>-</td></tr>
<tr><td>7.6.</td><td>Experimental design of test set</td><td>TDC’s protocol is followed for random data splitting into training (70%), validation (10%) and test (20%) subsets.<pre><code>from tdc.single_pred import Tox
data = Tox(name = 'AMES')
df = data.get_data()
split = data.get_split(method = 'random')</code></pre></td></tr>
</table>

<table>
<tr><td>7.7.</td><td>Predictivity - Statistics obtained by external validation</td><td>

Statistics	Test set
MCC	0.613
Accuracy	0.808
Sensitivity/Recall	0.815
Specificity	0.799
Precision	0.831

</td></tr>
<tr><td>7.8.</td><td>Predictivity - Assessment of the external validation set</td><td>-</td></tr>
<tr><td>7.9.</td><td>Comments on the external validation of the model</td><td>-</td></tr>
<tr><td>8</td><td>Providing a mechanistic interpretation - OECD Principle 5: “A MECHANISTIC INTERPRETATION, IF POSSIBLE”</td><td>PRINCIPLE 5: “A MECHANISTIC INTERPRETATION, IF POSSIBLE”. According to PRINCIPLE 5, a (Q)SAR should be associated with a mechanistic interpretation, if possible.</td></tr>
<tr><td>8.1.</td><td>Mechanistic basis of the model</td><td>-</td></tr>
<tr><td>8.2.</td><td>A priori or a posteriori mechanistic interpretation</td><td>A posteriori mechanistic interpretation. The use of the GCNN model allows to calculate and visualize the contributions of heavy atoms within the molecule to the prediction of mutagenicity, enhancing in this manner the model’s interpretability. In brief, the atomic contribution is calculated as the predicted probability difference between the whole molecule, and the remaining part of the molecule after atom removal. To better visualize atomic contributions, through a colour mapping the weights are normalized by dividing by the maximum absolute weight value. Atoms highlighted in pink colour contribute positively to the mutagenic probability (their presence enhances mutagenicity) whereas green colour indicates negative contribution to the mutagenic probability.</td></tr>
<tr><td>8.3.</td><td>Other information about the mechanistic interpretation</td><td>-</td></tr>
<tr><td>9</td><td>Miscellaneous information</td><td></td></tr>
<tr><td>9.1.</td><td>Comments</td><td>-</td></tr>
<tr><td>9.2.</td><td>Bibliography</td><td>-</td></tr>
<tr><td>9.3</td><td>Supporting information</td><td>The curated (only compounds with unambiguous stereochemistry) and enriched dataset (2D, 3D descriptors) is available in the chemPharos database, where the training, validation, and test observations are clearly indicated: https://db.chempharos.eu/datasets/Datasets.zul?datasetID=ds18</td></tr>
</table>

QMRF report 3: XGBoost

	**Element**	**Explanation**
**1.**	**QSAR identifier**	
1.1.	QSAR identifier (title)	Pólis - Classification of chemicals mutagenicity (Ames positive/negative) based on the XGBoost methodology

1.2	Other related models	Xu, C., Cheng, F., Chen, L., Du, Z., Li, W., Liu, G., ... & Tang, Y. (2012). In silico prediction of chemical Ames mutagenicity. *Journal of chemical information and modeling*, 52(11), 2840-2847. - https://doi.org/10.1021/ci300400a<br><br>Other models based on this dataset can be found in the TDC database: https://tdcommons.ai/benchmark/admet_group/21ames/
1.3.	Software coding the model	Isalos v2.0.0<br>Link to the web service:<br>https://www.enaloscloud.novamechanics.com/insight/polis/
**2.**	**General information**	
2.0	Abstract	A machine learning model for the classification of chemicals as “mutagens”/Ames positive or “non-mutagens”/Ames negative based on similarities, 2D and 3D descriptors. The relevant paper describing this model is in preparation.
2.1.	Date of QMRF	March 3, 2026
2.2.	QMRF author(s) and contact details	Dimitra-Danai Varsou, varsou@novamechanics.com<br>Andreas Tsoumanis, tsoumanis@novamechanics.com<br>Georgia Melagraki, georgiamelagraki@gmail.com<br>Antreas Afantitis, afantitis@novamechanics.com
2.3.	Date of QMRF update(s)	-
2.4.	QMRF update(s)	-
2.5.	Model developer(s) and contact details	Dimitra-Danai Varsou, varsou@novamechanics.com<br>Andreas Tsoumanis, tsoumanis@novamechanics.com<br>Georgia Melagraki, georgiamelagraki@gmail.com<br>Antreas Afantitis, afantitis@novamechanics.com
2.6.	Date of model development and/or publication	2025
2.7.	Reference(s) to main scientific papers and/or software package	Paper in preparation
2.8.	Availability of information about the model	The model is proprietary: the source code is confidential; however, the description of the modelling workflow is presented in the original research article, training and validation sets are available as supplementary information of the original research article and the model is implemented as a public web service.
2.9.	Availability of another QMRF for exactly the same model	-
**3**	**Defining the endpoint - OECD Principle 1: “A DEFINED ENDPOINT"**	**PRINCIPLE 1: “A DEFINED ENDPOINT". ENDPOINT refers to any physicochemical, biological, or environmental property/activity/effect that can be measured and therefore modelled. The intent of PRINCIPLE 1 (a (Q)SAR should be associated with a defined endpoint) is to ensure clarity in the endpoint being predicted by a given model, since a given endpoint could be determined by different experimental protocols and under different experimental conditions. It is therefore important to identify the experimental system and test conditions that is being modelled by the (Q)SAR.**
3.1.	Species	Bacteria (*Salmonella typhimurium*, *Escherichia coli*)
3.2.	Endpoint	Endpoint: detection of gene (point) mutations<br>The experimental protocol for the Ames bacterial reverse mutation test is covered by the OECD Test Guideline (TG) 471

<table>
<tr><td>3.3</td><td>Comment on endpoint</td><td>Exposure to amino-acid requiring strains (histidine-Salmonella typhimurium or tryptophan-Escherichia coli, respectively)</td></tr>
<tr><td>3.4.</td><td>Endpoint units</td><td>Classification: Ames positive or negative. Positive results from the bacterial reverse mutation test indicate that a substance induces point mutations by base substitutions or frameshifts in the genome of either Salmonella typhimurium and/or Escherichia coli.</td></tr>
<tr><td>3.5.</td><td>Dependent variable</td><td>Ames mutagenicity class: “Mutagenic” (Ames positive) or “Non-mutagenic” (Ames negative)</td></tr>
<tr><td>3.6.</td><td>Experimental protocol</td><td>See OECD Test Guideline (TG) 471. Data was collected and curated as described in Xu, C., Cheng, F., Chen, L., Du, Z., Li, W., Liu, G., ... & Tang, Y. (2012). In silico prediction of chemical Ames mutagenicity. Journal of chemical information and modeling, 52(11), 2840-2847. - https://doi.org/10.1021/ci300400a</td></tr>
<tr><td>3.7.</td><td>Endpoint data quality and variability</td><td>Details on the initial data collection and curation can be found in the work of Xu, C., Cheng, F., Chen, L., Du, Z., Li, W., Liu, G., ... & Tang, Y. (2012). In silico prediction of chemical Ames mutagenicity. Journal of chemical information and modeling, 52(11), 2840-2847. - https://doi.org/10.1021/ci300400a

Data are retrieved from the Therapeutics Data Commons (TDC) platform: https://tdcommons.ai/single_pred_tasks/tox/#ames-mutagenicity

<code>from tdc.single_pred import Tox</code>
<code>data = Tox(name = 'AMES')</code>
<code>df = data.get_data()</code>

An additional filtering of the chemical compounds is performed so that the 3D descriptors (see §4.5) correspond as closely as possible to the input compounds, excluding those with ambiguous stereochemistry. The RDKit EnumerateStereoisomers module is used to search for possible stereoisomers for each one of the compounds of the training, validation and test sets. Compounds that yield multiple stereoisomers due to unassigned stereocenters, are filtered out.

Classes distribution between sets after data curation:
<table>
<tr><th>Subset</th><th>All</th><th>Mutagens (Ames positive)</th><th>Non-mutagens (Ames negative)</th></tr>
<tr><td>Training</td><td>3724</td><td>2090</td><td>1634</td></tr>
<tr><td>Validation</td><td>549</td><td>325</td><td>224</td></tr>
<tr><td>Test</td><td>1089</td><td>601</td><td>488</td></tr>
<tr><td>Total</td><td>5362</td><td>3016</td><td>2346</td></tr>
</table></td></tr>
<tr><td>4</td><td>Defining the algorithm - OECD Principle 2: “AN UNAMBIGUOUS ALGORITHM”</td><td>PRINCIPLE 2: “AN UNAMBIGUOUS ALGORITHM”. The (Q)SAR estimate of an endpoint is the result of applying an ALGORITHM to a set of structural parameters which describe the chemical structure. The intent of PRINCIPLE 2 (a (Q)SAR should be associated with an unambiguous algorithm) is to ensure transparency in the model algorithm that generates predictions of an endpoint from information on chemical structure and/or physicochemical properties. In this context, algorithm refers to any mathematical equation, decision rule or output approach.</td></tr>
<tr><td>4.1.</td><td>Type of model</td><td>Decision trees/XGBoost</td></tr>
</table>

<table>
<tr><td>4.2.</td><td>Explicit algorithm</td><td>The XGBoost open-source library supports ensemble machine learning methods based on decision tree models. In an iterative procedure, trees are added to the ensemble so as to reduce the prediction error (loss) of previous models (in our case the objective function was the minimization of softmax probability loss).
<table>
<tr><th>Hyperparameters</th><th>Optimized hyperparameters</th></tr>
<tr><td>Booster</td><td>gbtree</td></tr>
<tr><td>Tree method</td><td>auto</td></tr>
<tr><td>Number of trees</td><td>100</td></tr>
<tr><td>Maximum tree depth</td><td>11</td></tr>
<tr><td>Learning rate (eta)</td><td>0.25</td></tr>
<tr><td>alpha</td><td>4</td></tr>
<tr><td>lambda</td><td>5</td></tr>
<tr><td>gamma</td><td>0</td></tr>
<tr><td>Minimum child weight</td><td>1</td></tr>
<tr><td>Subsampling rate</td><td>0.8</td></tr>
</table></td></tr>
<tr><td>4.3.</td><td>Descriptors in the model</td><td>
• D123: Average of molecular weight
• D173: Mohar order-2 index
• D186: Average vertex distance connectivity index
• D279: Total information content order-4 index
• D282: Structural information content order-1 index
• D287: Complementary information content order-0 index
• D297: Bond information content order-4 index
• D349: Molecular topological path index of order 09
• D357: Molecular topological multiple path index of order 09
• D358: Molecular topological multiple path index of order 10
• D366: Sum of topological distance between the vertices N and N
• D367: Sum of topological distance between the vertices N and P
• D413: Walk-returning count order-5
• D477: Geary topological structure autocorrelation length-7 weighted by atomic polarizabilities
• D478: Geary topological structure autocorrelation length-8 weighted by atomic polarizabilities
• D532: Lowest eigenvalue from Burden matrix weighted by masses order-1
• D533: Lowest eigenvalue from Burden matrix weighted by masses order-2
• D534: Lowest eigenvalue from Burden matrix weighted by masses order-3
• D535: Lowest eigenvalue from Burden matrix weighted by masses order-4
• D564: Highest eignevalue from Burden matrix weighted by masses order-1
• D588: Highest eignevalue from Burden matrix weighted by polarizabilities order-1
• D674: Number of group total hydroxyl groups
• D746: Number of group H attached to C0(sp3) no X attached to next C
• NPR1: Normalized Principal Moment of Inertia 1
• SpherocityIndex: An anisometry descriptor, it varies from zero for flat molecules, to one for totally spherical molecules
• PBF: Plane of best fit
</td></tr>
</table>

4.4.	Descriptor selection	Initial pool of descriptors: 777 Mold2 descriptors<br>Selection methods: low variance filtering, descriptors standardization, filtering out of descriptors with redundant values (80% threshold), "Best First" approach with "CfsSubset" evaluator.
4.5.	Algorithm and descriptor generation	Mold2 software, developed by the National Center for Toxicological Research (NCTR), calculates 777 unique molecular descriptors that capture the properties of small molecules, based on their 2D chemical structure. Additionally, 11 descriptors based on the 3D molecular structure (3D conformers) are calculated using RDKit.
4.6.	Software name and version for descriptor generation	RDKit v2024.3.6, Chem package<br>Enalos+ Mold2 KNIME node<br>Open Babel v3.1.1
4.7.	Chemicals/Descriptors ratio	Chemicals/ Descriptors ratio: 143.2
**5**	**Defining the applicability domain - OECD Principle 3: "A DEFINED DOMAIN OF APPLICABILITY"**	**PRINCIPLE 3: "A DEFINED DOMAIN OF APPLICABILITY". APPLICABILITY DOMAIN refers to the response and chemical structure space in which the model makes predictions with a given reliability. Ideally the applicability domain should express the structural, physicochemical and response space of the model. The CHEMICAL STRUCTURE (x variable) space can be expressed by information on physicochemical properties and/or structural fragments. The RESPONSE (y variable) can be any physicochemical, biological or environmental effect that is being predicted. According to PRINCIPLE 3 a (Q)SAR should be associated with a defined domain of applicability. Section 5 can be repeated (e.g., 5.a, 5.b, 5.c, etc) as many times as necessary if more than one method has been used to assess the applicability domain.**
5.1.	Description of the applicability domain of the model	The applicability domain (APD) is defined by fixed boundaries, the APD threshold (§5.2), calculated by considering Euclidean distances between all molecules in the training set. The distance of a test compound to its nearest neighbour in the training set is compared to the predefined applicability domain threshold. If the distance is beyond this threshold, then the prediction is considered unreliable.
5.2.	Method used to assess the applicability domain	The distance of each test compound to each nearest neighbour of the training compounds is compared to a predefined threshold<br>$$AD_{descriptors} = \langle d \rangle + Z\sigma$$<br>if this distance is lower than the threshold then it falls within the AD of the model, and its mutagenicity prediction can be considered reliable. To define the AD threshold, Euclidean distances between all training compounds based on the selected descriptors are calculated, as well as the average value of these distances (D_{av}). In a next step the new average value $\langle d \rangle$ and standard deviation σ of the distances included in the subset of training compounds, which have lower distance than D_{av}, are calculated. Z, is an empirical cut-off value, the default value is equal to 0.5.
5.3.	Software name and version for applicability domain assessment	Isalos v2.0.0<br>Function: Statistics → Applicability Domain → APD
5.4.	Limits of applicability	APD threshold = 5.421

<table>
<tr><th>6</th><th>Defining goodness-of-fit and robustness (internal validation) – OECD Principle 4: “APPROPRIATE MEASURES OF GOODNESS-OF-FIT, ROBUSTENESS AND PREDICTIVITY”</th><th>PRINCIPLE 4: “APPROPRIATE MEASURES OF GOODNESS-OF-FIT, ROBUSTENESS AND PREDICTIVITY”. PRINCIPLE 4 expresses the need to perform validation to establish the performance of the model. GOODNESS-OF-FIT and ROBUSTNESS refer to the internal model performance.</th></tr>
<tr><td>6.1.</td><td>Availability of the training set</td><td>Available at the ChemPharos database:
https://db.chempharos.eu/datasets/Datasets.zul?datasetID=ds18

Training compounds are labelled accordingly, see column “Subset” column (ALY).</td></tr>
<tr><td>6.2.</td><td>Available information for the training set</td><td>Chemical names (common names and IUPAC names)
SMILES
Molecular formulas
InChI codes
InChIKeys
PubChem URLs
CIDs</td></tr>
<tr><td>6.3.</td><td>Data for each descriptor variable for the training set</td><td>Available at the ChemPharos database:
https://db.chempharos.eu/datasets/Datasets.zul?datasetID=ds18

For each compound, 777 Mold2 (columns D-ACZ), and 11 3D RDKit descriptors (columns ALC-ALM) are available. The subset of descriptors used for modelling are presented in §4.3.</td></tr>
<tr><td>6.4.</td><td>Data for the dependent variable for the training set</td><td>Available at the ChemPharos database:
https://db.chempharos.eu/datasets/Datasets.zul?datasetID=ds18

Each compound is labelled (“Y”-column C) as either mutagenic/Ames positive (class 1) or non-mutagenic/Ames negative (class 0).</td></tr>
<tr><td>6.5.</td><td>Other information about the training set</td><td>-</td></tr>
<tr><td>6.6.</td><td>Pre-processing of data before modelling</td><td>Descriptors are standardized prior to feature selection with z-score (Gaussian) transformation. This linear transformation ensures that normalized values present a mean value equal to 0 and standard deviation equal to 1.</td></tr>
<tr><td>6.7.</td><td>Statistics for goodness-of-fit</td><td><table>
<tr><th>Statistics</th><th>Validation set</th></tr>
<tr><td>MCC</td><td>0.523</td></tr>
<tr><td>Accuracy</td><td>0.770</td></tr>
<tr><td>Sensitivity/Recall</td><td>0.815</td></tr>
<tr><td>Specificity</td><td>0.705</td></tr>
<tr><td>Precision</td><td>0.801</td></tr>
</table></td></tr>
<tr><td>6.8.</td><td>Robustness - Statistics obtained by leave-one-out cross-validation</td><td>-</td></tr>
<tr><td>6.9.</td><td>Robustness - Statistics obtained by leave-many-out cross-validation</td><td><table>
<tr><th>Statistics</th><th>Training set (5-fold CV)</th></tr>
<tr><td>MCC</td><td>0.584</td></tr>
<tr><td>Accuracy</td><td>0.795</td></tr>
<tr><td>Sensitivity/Recall</td><td>0.824</td></tr>
<tr><td>Precision</td><td>0.814</td></tr>
</table></td></tr>
<tr><td>6.10.</td><td>Robustness - Statistics obtained by Y-scrambling</td><td>Statistics (test set) for 5 iterations with scrambled endpoint values:
<table>
<tr><th>Y-Randomization</th><th>Accuracy</th><th>MCC</th></tr>
<tr><td># 1</td><td>0.514</td><td>-0.003</td></tr>
</table></td></tr>
</table>

<table>
<tr><td></td><td></td><td><table>
<tr><td># 2</td><td>0.538</td><td>0.051</td></tr>
<tr><td># 3</td><td>0.494</td><td>-0.048</td></tr>
<tr><td># 4</td><td>0.517</td><td>0.004</td></tr>
<tr><td># 5</td><td>0.512</td><td>-0.007</td></tr>
</table></td></tr>
<tr><td>6.11.</td><td>Robustness - Statistics obtained by bootstrap</td><td>-</td></tr>
<tr><td>6.12.</td><td>Robustness - Statistics obtained by other methods</td><td>-</td></tr>
<tr><td>7</td><td>Defining predictivity (external validation) – OECD Principle 4: “APPROPRIATE MEASURES OF GOODNESS-OF-FIT, ROBUSTENESS AND PREDICTIVITY”</td><td>PRINCIPLE 4: “APPROPRIATE MEASURES OF GOODNESS-OF-FIT, ROBUSTENESS AND PREDICTIVITY”. PRINCIPLE 4 expresses the need to perform validation to establish the performance of the model. PREDICTIVITY refers to the external model validation. Section 7 can be repeated (e.g., 7.a, 7.b, 7.c, etc) as many times as necessary if more validation studies need to be reported in the QMRF.</td></tr>
<tr><td>7.1.</td><td>Availability of the external validation set</td><td>Available at the ChemPharos database:
https://db.chempharos.eu/datasets/Datasets.zul?datasetID=ds18

Testing compounds are labelled accordingly, see column “Subset” column (ALY).</td></tr>
<tr><td>7.2.</td><td>Available information for the external validation set</td><td>Chemical names (common names and IUPAC names)
SMILES
Molecular formulas
InChI codes
InChIKeys
PubChem URLs
CIDs</td></tr>
<tr><td>7.3.</td><td>Data for each descriptor variable for the external validation set</td><td>Available at the ChemPharos database:
https://db.chempharos.eu/datasets/Datasets.zul?datasetID=ds18

For each compound, 777 Mold2 (columns D-ACZ), and 11 3D RDKit descriptors (columns ALC-ALM) are available. The subset of descriptors used in the model are presented in §4.3.</td></tr>
<tr><td>7.4.</td><td>Data for the dependent variable for the external validation set</td><td>Available at the ChemPharos database:
https://db.chempharos.eu/datasets/Datasets.zul?datasetID=ds18

Each compound is labelled (“Y”-column C) as either mutagenic/Ames positive (class 1) or non-mutagenic/Ames negative (class 0).</td></tr>
<tr><td>7.5.</td><td>Other information about the external validation set</td><td>-</td></tr>
<tr><td>7.6.</td><td>Experimental design of test set</td><td>TDC’s protocol is followed for random data splitting into training (70%), validation (10%) and test (20%) subsets.

from tdc.single_pred import Tox
data = Tox(name = 'AMES')
df = data.get_data()
split = data.get_split(method = 'random')</td></tr>
</table>

7.7.	Predictivity - Statistics obtained by external validation	<table><tr><td>Statistics</td><td>Test set</td></tr><tr><td>MCC</td><td>0.607</td></tr><tr><td>Accuracy</td><td>0.805</td></tr><tr><td>Sensitivity/Recall</td><td>0.817</td></tr><tr><td>Specificity</td><td>0.791</td></tr><tr><td>Precision</td><td>0.828</td></tr></table>
7.8.	Predictivity - Assessment of the external validation set	99.4% of the test set compounds fall inside the applicability domain limits of the model.
7.9.	Comments on the external validation of the model	The test set was normalized using the Gaussian normalization function, which was also applied on the training set.
**8**	**Providing a mechanistic interpretation - OECD Principle 5: "A MECHANISTIC INTERPRETATION, IF POSSIBLE"**	**PRINCIPLE 5: "A MECHANISTIC INTERPRETATION, IF POSSIBLE". According to PRINCIPLE 5, a (Q)SAR should be associated with a mechanistic interpretation, if possible.**
8.1.	Mechanistic basis of the model	Descriptors are described in §4.3.
8.2.	A priori or a posteriori mechanistic interpretation	A posteriori mechanistic interpretation. A SHAP analysis was performed in Python using Tree SHAP to evaluate the influence of each selected descriptor in the XGBoost model's mutagenicity predictions. The SHAP analysis highlighted mainly topological and topochemical properties. To begin with, Burden matrix eigenvalues (D532, D533, D535, D534, D564, D588) and information content indices (D279, D282, D297) emerged as critical factors, all of which quantify molecular topology, accounting for atomic contributions and molecular diversity of atoms and bonds, respectively. Although these descriptors do not directly indicate known mutagenic substructures, they suggest that the overall electron distribution and molecular connectivity are important for how a compound is metabolized and how it may form reactive intermediates, ultimately influencing mutagenic potential. Additionally, multiple path indices (D357, D358) were important, indicating that the spatial arrangement of topological features also contribute to mutagenicity predictions. Meanwhile, the SpherocityIndex and PBF were found among the highly contributing descriptors: for instance, SHAP analysis indicates that lower PBF values, corresponding to more planar compounds, are associated with increased mutagenic potential, which is consistent with literature indicating planarity of molecules, as a possible factor for DNA intercalation, especially in polycyclic aromatic systems.<br>Another top-ranked descriptor (D367) was the sum of topological distance between the vertices of nitrogen (N) and phosphorus (P) atoms, with higher values (e.g., in cases of structurally distant N and P atoms in different branches or regions, in cases of elongated molecules or in cases where the N and P atoms are sparse) contributing to mutagenicity. Lower D367 values indicate direct P-N bonds within the molecule or that these atoms are possibly clustered within the same functional region. Both N and P-containing compounds are considered important therapeutic agents in the medicinal field due to their bioavailability, selectivity and biocompatibility. Among the top-ranked descriptors was the number of total hydroxyl groups (D674), which can influence a compound's solubility, metabolic pathways, and overall biological reactivity, and as a result they can potentially alter mutagenicity risk. Finally, the negative SHAP values associated with the compounds average molecular weight (D123) indicate that, within the XGBoost model, molecules with lower average molecular weight tend to be predicted as more mutagenic. Heavy atoms such as chlorine and iodine may be associated with toxic structural alerts.

8.3.	Other information about the mechanistic interpretation	-
**9**	**Miscellaneous information**	
9.1.	Comments	-
9.2.	Bibliography	-
9.3	Supporting information	The curated and enriched dataset is available in the chemPharos database, where the training, validation, and test observations are clearly indicated: https://db.chempharos.eu/datasets/Datasets.zul?datasetID=ds18

QMRF report 4: Consensus model

	Element	Explanation
**1.**	**QSAR identifier**	
1.1.	QSAR identifier (title)	Pólis - Classification of chemicals mutagenicity (Ames positive/negative) based on the consensus scheme (guided-kNN, GCNN & XGBoost)
1.2	Other related models	Xu, C., Cheng, F., Chen, L., Du, Z., Li, W., Liu, G., ... & Tang, Y. (2012). In silico prediction of chemical Ames mutagenicity. *Journal of chemical information and modeling*, 52(11), 2840-2847. - https://doi.org/10.1021/ci300400a<br><br>Other models based on this dataset can be found in the TDC database: https://tdcommons.ai/benchmark/admet_group/21ames/
1.3.	Software coding the model	Isalos v2.0.0<br>Link to the web service:<br>https://www.enaloscloud.novamechanics.com/insight/polis/
**2.**	**General information**	
2.0	Abstract	A consensus model for the classification of chemicals as “mutagens”/Ames positive or “non-mutagens”/Ames negative based on similarities, 2D and 3D descriptors. The relevant paper describing this model is in preparation.
2.1.	Date of QMRF	March 3, 2026
2.2.	QMRF author(s) and contact details	Dimitra-Danai Varsou, varsou@novamechanics.com<br>Andreas Tsoumanis, tsoumanis@novamechanics.com<br>Georgia Melagraki, georgiamelagraki@gmail.com<br>Antreas Afantitis, afantitis@novamechanics.com
2.3.	Date of QMRF update(s)	-
2.4.	QMRF update(s)	-
2.5.	Model developer(s) and contact details	Dimitra-Danai Varsou, varsou@novamechanics.com<br>Andreas Tsoumanis, tsoumanis@novamechanics.com<br>Georgia Melagraki, georgiamelagraki@gmail.com<br>Antreas Afantitis, afantitis@novamechanics.com
2.6.	Date of model development and/or publication	2025
2.7.	Reference(s) to main scientific papers and/or software package	Paper in preparation

2.8.	Availability of information about the model	The model is proprietary: the source code is confidential; however, the description of the modelling workflow is presented in the original research article, training and validation sets are available as supplementary information of the original research article and the model is implemented as a public web service.
2.9.	Availability of another QMRF for exactly the same model	-
**3**	**Defining the endpoint - OECD Principle 1: "A DEFINED ENDPOINT"**	**PRINCIPLE 1: "A DEFINED ENDPOINT". ENDPOINT refers to any physicochemical, biological, or environmental property/activity/effect that can be measured and therefore modelled. The intent of PRINCIPLE 1 (a (Q)SAR should be associated with a defined endpoint) is to ensure clarity in the endpoint being predicted by a given model, since a given endpoint could be determined by different experimental protocols and under different experimental conditions. It is therefore important to identify the experimental system and test conditions that is being modelled by the (Q)SAR.**
3.1.	Species	Bacteria (*Salmonella typhimurium*, *Escherichia coli*)
3.2.	Endpoint	Endpoint: detection of gene (point) mutations<br>The experimental protocol for the Ames bacterial reverse mutation test is covered by the OECD Test Guideline (TG) 471
3.3	Comment on endpoint	Exposure to amino-acid requiring strains (histidine-*Salmonella typhimurium* or tryptophan-*Escherichia coli*, respectively)
3.4.	Endpoint units	Classification: Ames positive or negative. Positive results from the bacterial reverse mutation test indicate that a substance induces point mutations by base substitutions or frameshifts in the genome of either *Salmonella typhimurium* and/or *Escherichia coli*.
3.5.	Dependent variable	Ames mutagenicity class: "Mutagenic" (Ames positive) or "Non-mutagenic" (Ames negative)
3.6.	Experimental protocol	See OECD Test Guideline (TG) 471. Data was collected and curated as described in Xu, C., Cheng, F., Chen, L., Du, Z., Li, W., Liu, G., ... & Tang, Y. (2012). In silico prediction of chemical Ames mutagenicity. *Journal of chemical information and modeling*, 52(11), 2840-2847. - https://doi.org/10.1021/ci300400a
3.7.	Endpoint data quality and variability	Details on the initial data collection and curation can be found in the work of Xu, C., Cheng, F., Chen, L., Du, Z., Li, W., Liu, G., ... & Tang, Y. (2012). In silico prediction of chemical Ames mutagenicity. *Journal of chemical information and modeling*, 52(11), 2840-2847. - https://doi.org/10.1021/ci300400a<br><br>Data are retrieved from the Therapeutics Data Commons (TDC) platform: https://tdcommons.ai/single_pred_tasks/tox/#ames-mutagenicity<br><br>`from tdc.single_pred import Tox`<br>`data = Tox(name = 'AMES')`<br>`df = data.get_data()`<br><br>An additional filtering of the chemical compounds is performed so that the 3D descriptors (see §4.5) correspond as closely as possible to the input compounds, excluding those with ambiguous stereochemistry. The RDKit EnumerateStereoisomers module is used to search for possible stereoisomers for each one of the compounds of the training, validation and test sets. Compounds that yield multiple stereoisomers due to unassigned stereocenters, are filtered out.<br><br>Classes distribution between sets before and after data curation:

Initial dataset (GCNN)			
Subset	All	*Mutagens (Ames positive)*	Non-mutagens (Ames negative)
Training	5076	2741	2335
Validation	726	415	311
Test	1453	795	658
Total	7255	3951	3304
Curated set (guided-kNN and XGBoost)			
Subset	All	*Mutagens (Ames positive)*	Non-mutagens (Ames negative)
Training	3724	2090	1634
Validation	549	325	224
Test	1089	601	488
Total	5362	3016	2346

**4**	**Defining the algorithm - OECD Principle 2: "AN UNAMBIGUOUS ALGORITHM"**	**PRINCIPLE 2: "AN UNAMBIGUOUS ALGORITHM". The (Q)SAR estimate of an endpoint is the result of applying an ALGORITHM to a set of structural parameters which describe the chemical structure. The intent of PRINCIPLE 2 (a (Q)SAR should be associated with an unambiguous algorithm) is to ensure transparency in the model algorithm that generates predictions of an endpoint from information on chemical structure and/or physicochemical properties. In this context, algorithm refers to any mathematical equation, decision rule or output approach.**
4.1.	Type of model	Consensus model
4.2.	Explicit algorithm	Each model (guided-kNN, GCNN & XGBoost) casts a "vote" for a class, and the class with the highest number of votes (majority vote) is selected as the final prediction.<br><br>In case predictions cannot be produced from the guided-kNN and XGBoost models due to the inability to generate 3D conformers, the consensus prediction is based solely on the GCNN model.
4.3.	Descriptors in the model	Molecular graphs (GCNN), 2D and 3D descriptors (XGBoost and guided-kNN). See details in respective QMRFs.
4.4.	Descriptor selection	See details in respective QMRFs.
4.5.	Algorithm and descriptor generation	See details in respective QMRFs.
4.6.	Software name and version for descriptor generation	See details in respective QMRFs.
4.7.	Chemicals/Descriptors ratio	See details in respective QMRFs.
**5**	**Defining the applicability domain - OECD Principle 3: "A DEFINED DOMAIN OF APPLICABILITY"**	**PRINCIPLE 3: "A DEFINED DOMAIN OF APPLICABILITY". APPLICABILITY DOMAIN refers to the response and chemical structure space in which the model makes predictions with a given reliability. Ideally the applicability domain should express the structural, physicochemical and response space of the model. The CHEMICAL STRUCTURE (x variable) space can be expressed by information on physicochemical properties and/or structural fragments. The RESPONSE (y variable) can be any physicochemical, biological or environmental effect that is being predicted. According to PRINCIPLE 3 a (Q)SAR should be associated with a defined domain of applicability. Section 5 can be repeated (e.g., 5.a, 5.b, 5.c, etc) as many times as necessary if more than one method has been used to assess the applicability domain.**

5.1.	Description of the applicability domain of the model	We consider that the AD of the two models (guided-kNN and XGBoost) is well-defined covering the physicochemical and structural domain and thus, can cover the reliability of the predictions of the GCNN model as well, considering that the same training compounds are used in all three methodologies. To increase confidence in the consensus predictions, the outcome of the two AD methods (whether a prediction lies within the respective AD limits) is combined and an overall decision on the reliability of the predictions is made.
5.2.	Method used to assess the applicability domain	Voting scheme: The degree of reliability for each prediction is characterized as "low" (the query compound falls outside both AD limits), "moderate" (only one of the two AD limits is met), and "high" (the query compound falls within both AD limits).
5.3.	Software name and version for applicability domain assessment	Isalos v2.0.0
5.4.	Limits of applicability	See respective QMRFs.
**6**	**Defining goodness-of-fit and robustness (internal validation) – OECD Principle 4: "APPROPRIATE MEASURES OF GOODNESS-OF-FIT, ROBUSTENESS AND PREDICTIVITY"**	**PRINCIPLE 4: "APPROPRIATE MEASURES OF GOODNESS-OF-FIT, ROBUSTENESS AND PREDICTIVITY". PRINCIPLE 4 expresses the need to perform validation to establish the performance of the model. GOODNESS-OF-FIT and ROBUSTNESS refer to the internal model performance.**
6.1.	Availability of the training set	The complete dataset can be directly retrieved from the TDC database: https://tdcommons.ai/single_pred_tasks/tox/#ames-mutagenicity<br><br>The curated dataset is available at the ChemPharos database: https://db.chempharos.eu/datasets/Datasets.zul?datasetID=ds18<br>Training compounds are labelled accordingly, see column "Subset" column (ALY).
6.2.	Available information for the training set	Complete set:<br>SMILES<br><br>Curated set:<br>Chemical names (common names and IUPAC names)<br>SMILES<br>Molecular formulas<br>InChI codes<br>InChIKeys<br>PubChem URLs<br>CIDs
6.3.	Data for each descriptor variable for the training set	For the curated dataset<br>https://db.chempharos.eu/datasets/Datasets.zul?datasetID=ds18<br><br>For each compound, 777 Mold2 (columns D-ACZ), 210 2D RDKit (columns ADA-ALB) and 11 3D RDKit descriptors (columns ALC-ALM) are available.
6.4.	Data for the dependent variable for the training set	Each compound is labelled ("Y") as either mutagenic/Ames positive (class 1) or non-mutagenic/Ames negative (class 0).<br><br>For the curated dataset<br>https://db.chempharos.eu/datasets/Datasets.zul?datasetID=ds18, the label can be found in "Y"-column C.
6.5.	Other information about the training set	-
6.6.	Pre-processing of data before modelling	See details in respective QMRFs.

6.7.	Statistics for goodness-of-fit	-
6.8.	Robustness - Statistics obtained by leave-one-out cross-validation	-
6.9.	Robustness - Statistics obtained by leave-many-out cross-validation	-
6.10.	Robustness - Statistics obtained by Y-scrambling	-
6.11.	Robustness - Statistics obtained by bootstrap	To assess the robustness of our predictive workflow, we also performed a sensitivity analysis to quantify the impact of stochasticity in the 3D conformer generation step. For this reason, an external set of compounds was selected ("logP dataset" from Titania tool - https://enaloscloud.novamechanics.com/EnalosWebApps/titania/) comprising of 14207 molecules. Any molecule that failed sanitization was removed from the dataset, leaving 13844 compounds available for further analysis. Next, conformer generation was performed using ETKDGv3 and UFF optimization followed by 3D descriptor calculation and final mutagenicity prediction. This process was repeated 20 times using in each run a different random seed to initiate the conformer embedding process. The results demonstrated a high degree of stability, with the consensus model predictions varying by only approximately 0.3% across all runs. Given that the model's overall accuracy is 83% (see §7.7), this 0.3% variability is negligible.
6.12.	Robustness - Statistics obtained by other methods	-
**7**	**Defining predictivity (external validation) – OECD Principle 4: "APPROPRIATE MEASURES OF GOODNESS-OF-FIT, ROBUSTENESS AND PREDICTIVITY"**	**PRINCIPLE 4: "APPROPRIATE MEASURES OF GOODNESS-OF-FIT, ROBUSTENESS AND PREDICTIVITY". PRINCIPLE 4 expresses the need to perform validation to establish the performance of the model. PREDICTIVITY refers to the external model validation. Section 7 can be repeated (e.g., 7.a, 7.b, 7.c, etc) as many times as necessary if more validation studies need to be reported in the QMRF.**
7.1.	Availability of the external validation set	The complete dataset can be directly retrieved from the TDC database: https://tdcommons.ai/single_pred_tasks/tox/#ames-mutagenicity<br><br>The curated dataset is available at the ChemPharos database: https://db.chempharos.eu/datasets/Datasets.zul?datasetID=ds18<br>Testing compounds are labelled accordingly, see column "Subset" column (ALY).
7.2.	Available information for the external validation set	Complete set:<br>SMILES<br><br>Curated set:<br>Chemical names (common names and IUPAC names)<br>SMILES<br>Molecular formulas<br>InChI codes<br>InChIKeys<br>PubChem URLs<br>CIDs

<table>
<tr><td>7.3.</td><td>Data for each descriptor variable for the external validation set</td><td>For the curated dataset
https://db.chempharos.eu/datasets/Datasets.zul?datasetID=ds18

For each compound, 777 Mold2 (columns D-ACZ), 210 2D RDKit (columns ADA-ALB) and 11 3D RDKit descriptors (columns ALC-ALM) are available.</td></tr>
<tr><td>7.4.</td><td>Data for the dependent variable for the external validation set</td><td>Each compound is labelled (“Y”) as either mutagenic/Ames positive (class 1) or non-mutagenic/Ames negative (class 0).

For the curated dataset
https://db.chempharos.eu/datasets/Datasets.zul?datasetID=ds18, the label can be found in “Y”-column C.</td></tr>
<tr><td>7.5.</td><td>Other information about the external validation set</td><td>-</td></tr>
<tr><td>7.6.</td><td>Experimental design of test set</td><td>TDC’s protocol is followed for random data splitting into training (70%), validation (10%) and test (20%) subsets.

from tdc.single_pred import Tox
data = Tox(name = 'AMES')
df = data.get_data()
split = data.get_split(method = 'random')

For fair comparisons against the guided-kNN and XGBoost methodologies, the evaluation metrics for the GCNN model are calculated based on the curated test set predicted classes comprising of 1088 compounds.</td></tr>
<tr><td>7.7.</td><td>Predictivity - Statistics obtained by external validation</td><td><table>
<tr><td>Statistics</td><td>Test set</td></tr>
<tr><td>MCC</td><td>0.648</td></tr>
<tr><td>Accuracy</td><td>0.826</td></tr>
<tr><td>Sensitivity/Recall</td><td>0.855</td></tr>
<tr><td>Specificity</td><td>0.791</td></tr>
<tr><td>Precision</td><td>0.834</td></tr>
</table></td></tr>
<tr><td>7.8.</td><td>Predictivity - Assessment of the external validation set</td><td>-</td></tr>
<tr><td>7.9.</td><td>Comments on the external validation of the model</td><td>See details in respective QMRFs.</td></tr>
<tr><td>**8**</td><td>**Providing a mechanistic interpretation - OECD Principle 5: “A MECHANISTIC INTERPRETATION, IF POSSIBLE”**</td><td>**PRINCIPLE 5: “A MECHANISTIC INTERPRETATION, IF POSSIBLE”. According to PRINCIPLE 5, a (Q)SAR should be associated with a mechanistic interpretation, if possible.**</td></tr>
<tr><td>8.1.</td><td>Mechanistic basis of the model</td><td>-</td></tr>
<tr><td>8.2.</td><td>A priori or a posteriori mechanistic interpretation</td><td>-</td></tr>
<tr><td>8.3.</td><td>Other information about the mechanistic interpretation</td><td>-</td></tr>
<tr><td>**9**</td><td>**Miscellaneous information**</td><td></td></tr>
<tr><td>9.1.</td><td>Comments</td><td>-</td></tr>
<tr><td>9.2.</td><td>Bibliography</td><td>-</td></tr>
</table>

9.3	Supporting information	The curated (only compounds with unambiguous stereochemistry) and enriched dataset (2D, 3D descriptors) is available in the chemPharos database, where the training, validation, and test observations are clearly indicated: https://db.chempharos.eu/datasets/Datasets.zul?datasetID=ds18
