# Supplementary material for: From QSAR to deep learning: an interpretable comprehensive pipeline with a read-across approach for mutagenicity prediction via the Enalos Cloud Platform
Source: RSC Adv. 2026 Sep 9;16(43):53776–805. doi: 10.1039/d6ra05786a (PMC13557173; doi:10.1039/d6ra05786a)
Supplement: Supplementary file 3 [file RA-016-D6RA05786A-s003.pdf]

Supplementary Information (SI) for RSC Advances.
This journal is © The Royal Society of Chemistry 2026

From QSAR to Deep Learning: An interpretable comprehensive pipeline with a read-across approach for mutagenicity prediction via the Enalos Cloud Platform

Dimitra-Danai Varsou[1,2], Andreas Tsoumanis[2,3], Eleonora Marta Longhin[4], Maria Dusinska[4], Dimitris G. Mintis[2,3], Panagiotis D. Kolokathis[1,2], Konstantinos D. Papavasileiou[1,2], Iseult Lynch[5], Georgia Melagraki[6], Antreas Afantitis[1,2,3,7]

[1]NovaMechanics MIKE, Piraeus 18545, Greece
[2]Entelos Institute, Nicosia 2102, Cyprus
[3]NovaMechanics Ltd, Nicosia 1070, Cyprus
[4]Health Effects Laboratory, Department of Environmental Chemistry and Health Effects, NILU - Norwegian Institute for Air Research, Kjeller, Norway
[5]School of Geography, Earth and Environmental Sciences, University of Birmingham, B15 2TT Birmingham, UK
[6]Division of Physical Sciences and Applications, Hellenic Military Academy, 16673, Vari, Greece
[7]Department of Pharmacy, Frederick University, Nicosia 1036, Cyprus

Supporting Information

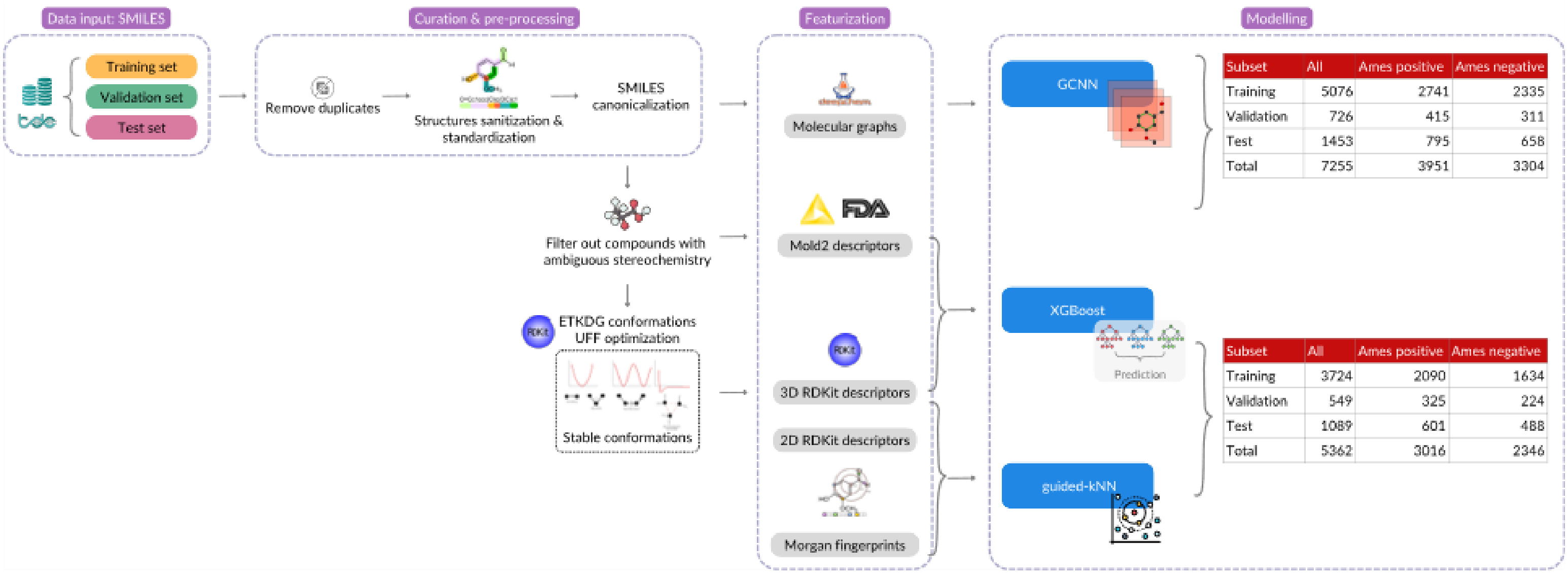

Figure S1: Data curation and featurization process.

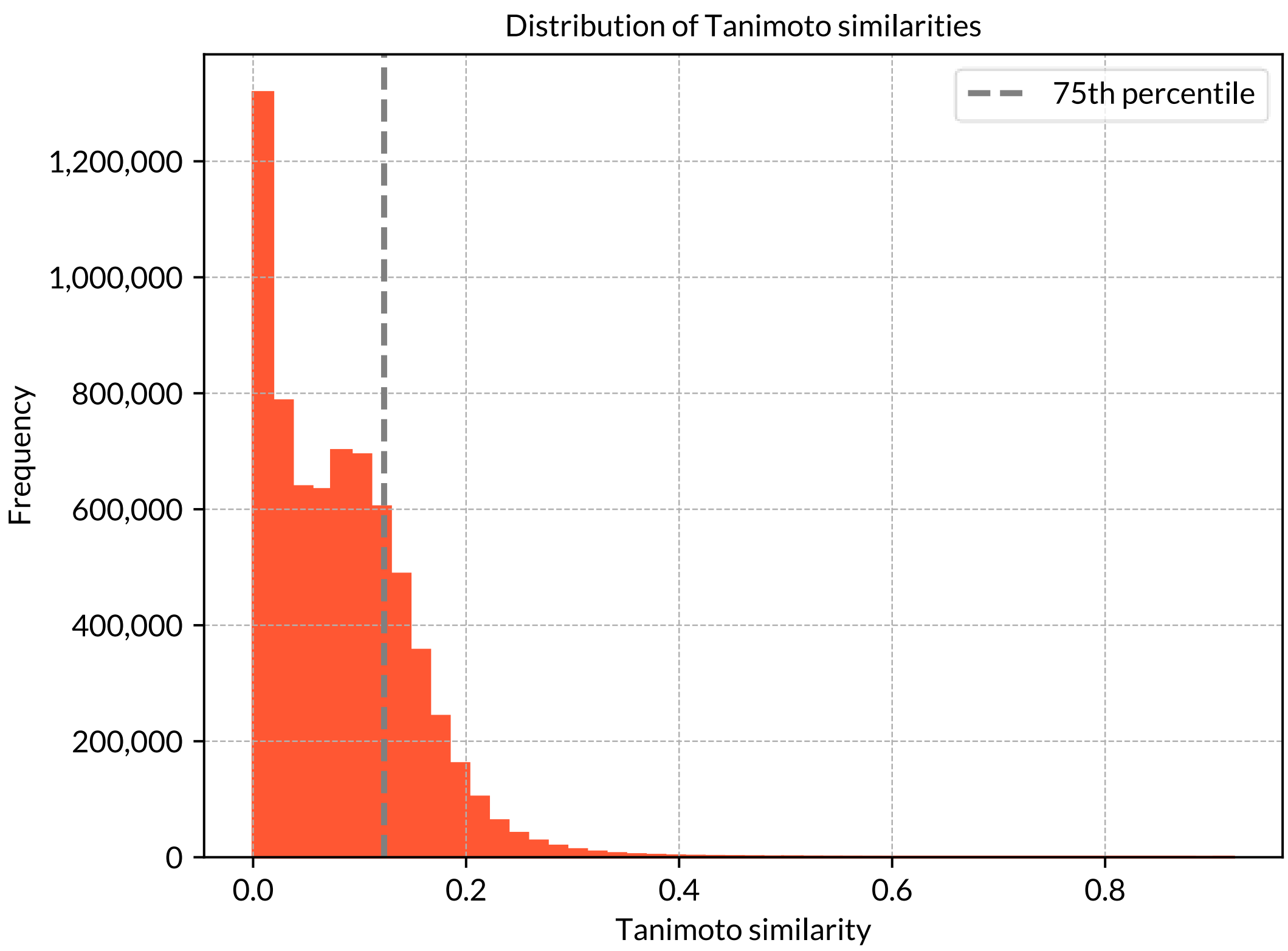

Figure S2: Distribution of Tanimoto similarities within the training compounds and depiction of the 75th percentile, selected as the S_{cutoff} value for the $AD_{fingerprint}$ methodology.

Table S1: Selected descriptors for the development of the guided-kNN model.[1–12]

Feature	Score	Description
SlogP_VSA8	330.81	A MOE-type descriptor that sums the van der Waals surface area (VSA) contributions of atoms assigned to bin 8 based on their atomic contributions to the logP (expressing hydrophobicity)
fr_nitro	253.87	Number of nitro groups
NumAromaticRings	253.66	Number of aromatic rings in the molecule
HallKierAlpha	235.40	The Hall-Kier alpha value for a molecule
RingCount	235.02	Total number of rings in the molecule
fr_bicyclic	204.46	Number of bicyclic rings
NumAromaticCarbocycles	202.80	Number of aromatic rings containing only carbon atoms.
fr_benzene	202.46	Number of benzene rings
BertzCT	201.05	A topological index meant to quantify complexity of molecules
fr_nitro_arom_nonortho	187.86	Number of non-ortho nitro benzene ring substituents
fr_nitro_arom	179.86	Number of nitro benzene ring substituents
PBF	179.75	Plane of best fit
PEOE_VSA2	169.90	A MOE-type descriptor that sums the van der Waals surface area (VSA) contributions of atoms assigned to bin 2 based on their partial equalization of orbital electronegativity (PEOE) charge values
AvgIpc	166.36	Average information content per atom
fr_NH0	165.93	Number of Tertiary amines

SMR_VSA5	157.61	A MOE-type descriptor that sums the van der Waals surface area (VSA) contributions of atoms assigned to bin 5 based on their atomic contributions to the MR (molecular refractivity)
SlogP_VSA10	147.46	A MOE-type descriptor that sums the van der Waals surface area (VSA) contributions of atoms assigned to bin 10 based on their atomic contributions to the logP (expressing hydrophobicity)
SpherocityIndex	137.55	An anisometry descriptor, it varies from zero for flat molecules, to one for totally spherical molecules
SMR_VSA7	132.93	A MOE-type descriptor that sums the van der Waals surface area (VSA) contributions of atoms assigned to bin 7 based on their atomic contributions to the MR (molecular refractivity)
SlogP_VSA5	130.81	A MOE-type descriptor that sums the van der Waals surface area (VSA) contributions of atoms assigned to bin 5 based on their atomic contributions to the logP (expressing hydrophobicity)
SMR_VSA10	127.21	A MOE-type descriptor that sums the van der Waals surface area (VSA) contributions of atoms assigned to bin 10 based on their atomic contributions to the MR (molecular refractivity)
SlogP_VSA4	116.32	A MOE-type descriptor that sums the van der Waals surface area (VSA) contributions of atoms assigned to bin 4 based on their atomic contributions to the logP (expressing hydrophobicity)
VSA_EState8	113.55	A MOE-type descriptor that sums the electrotopological state (EState) indices of atoms assigned to bin 8 based on their van der Waals surface area (VSA) contributions
FractionCSP3	106.53	Fraction of sp^3-hybridized carbon atoms
VSA_EState6	106.46	A MOE-type descriptor that sums the electrotopological state (EState) indices of atoms assigned to bin 6 based on their van der Waals surface area (VSA) contributions
PEOE_VSA13	106.04	A MOE-type descriptor that sums the van der Waals surface area (VSA) contributions of atoms assigned to bin 13 based on their partial equalization of orbital electronegativity (PEOE) charge values
Qed	105.19	Quantitative Estimate of Drug-likeness
FpDensityMorgan3	98.71	Fingerprint density for Morgan fingerprints (radius=3)
SlogP_VSA6	97.18	A MOE-type descriptor that sums the van der Waals surface area (VSA) contributions of atoms assigned to bin 6 based on their atomic contributions to the logP (expressing hydrophobicity)
NOCount	92.74	Number of Nitrogen and Oxygen atoms
VSA_EState3	74.02	A MOE-type descriptor that sums the electrotopological state (EState) indices of atoms assigned to bin 3 based on their van der Waals surface area (VSA) contributions
TPSA	72.74	Topological polar surface area
VSA_EState4	67.33	A MOE-type descriptor that sums the electrotopological state (EState) indices of atoms assigned to bin 4 based on their van der Waals surface area (VSA) contributions
NumHAcceptors	64.25	Number of Hydrogen Bond Acceptors

fr_unbrch_alkane	62.88	Number of unbranched alkanes of at least 4 members (excludes halogenated alkanes)
Chi4n	57.55	A valence molecular connectivity index calculated based on Rev. Comp. Chem., vol 2, 367-422 (1991).
PEOE_VSA8	56.48	A MOE-type descriptor that sums the van der Waals surface area (VSA) contributions of atoms assigned to bin 8 based on their partial equalization of orbital electronegativity (PEOE) charge values
VSA_EState7	55.94	A MOE-type descriptor that sums the electrotopological state (EState) indices of atoms assigned to bin 7 based on their van der Waals surface area (VSA) contributions
EState_VSA10	54.12	A MOE-type descriptor that sums the van der Waals surface area (VSA) contributions of atoms assigned to bin 10 based on their electrotopological state (EState) index
fr_COO2	53.82	Number of carboxylic acids
Chi1	51.98	A molecular connectivity index calculated from equations (1), (11) and (12) of Rev. Comp. Chem. vol 2, 367-422, (1991)
fr_ArN	51.78	Number of aromatic nitrogens
BalabanJ	51.78	A chemical distance-based topological index from Chem. Phys. Lett. vol 89, 399-404 (1982).
VSA_EState2	46.98	A MOE-type descriptor that sums the electrotopological state (EState) indices of atoms assigned to bin 2 based on their van der Waals surface area (VSA) contributions
NumRotatableBonds	46.42	Number of Rotatable Bonds
HeavyAtomCount	45.79	Number of heavy atoms of a molecule
SlogP_VSA3	44.53	A MOE-type descriptor that sums the van der Waals surface area (VSA) contributions of atoms assigned to bin 3 based on their atomic contributions to the logP (expressing hydrophobicity)
Chi3n	43.82	A valence molecular connectivity index calculated based on Rev. Comp. Chem., vol 2, 367-422 (1991).
NumHeteroatoms	38.55	Number of Heteroatoms
fr_aniline	35.32	Number of anilines

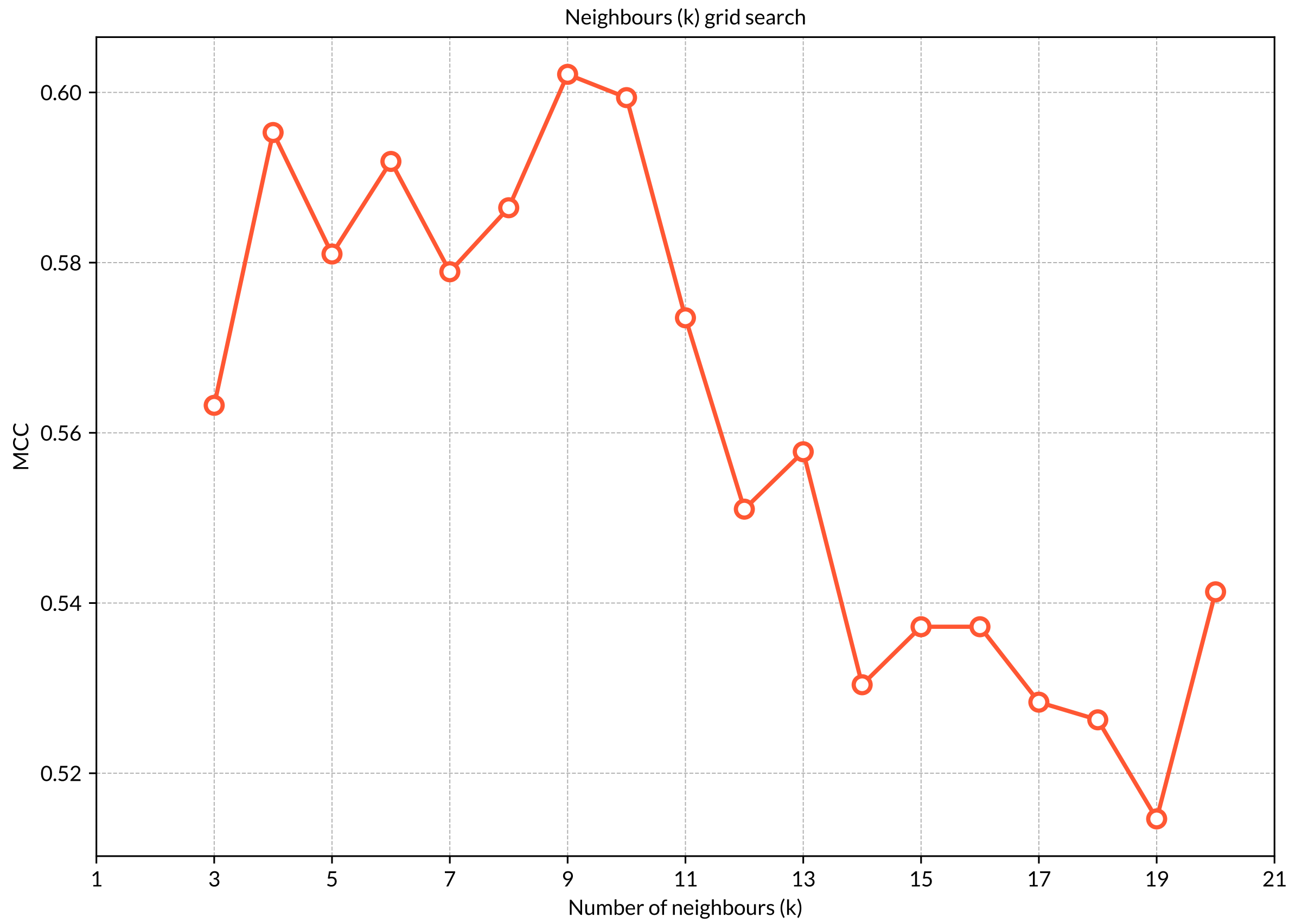

Figure S3: Grid search results for the selection of the k_{opt} value. The horizontal axis represents the number of k-neighbours, while the vertical axis shows the MCC score on the validation set.

Table S2: Y-randomization results, expressed as accuracy and MCC, when the shuffled-endpoint guided-kNN models (trained using $k = k_{opt}$) were applied on the original test set.

Y-Randomization	Accuracy	MCC
# 1	0.495	-0.038
# 2	0.522	0.018
# 3	0.472	-0.087
# 4	0.516	0.011
# 5	0.496	-0.035

Table S3: Y-randomization results, expressed as accuracy and MCC, when the shuffled-endpoint GCNN models (trained using the optimized hyperparameters) were applied on the original test set.

Y-Randomization	Accuracy	MCC
# 1	0.531	0.064
# 2	0.480	-0.030
# 3	0.539	0.041
# 4	0.515	0.004
# 5	0.522	0.036

Table S4: Selected descriptors for the development of the QSAR model.[9,13–15]

Descriptor	Description
D123	Average of molecular weight
D173	Mohar order-2 index
D186	Average vertex distance connectivity index
D279	Total information content order-4 index
D282	Structural information content order-1 index
D287	Complementary information content order-0 index
D297	Bond information content order-4 index
D349	Molecular topological path index of order 09
D357	Molecular topological multiple path index of order 09
D358	Molecular topological multiple path index of order 10
D366	Sum of topological distance between the vertices N and N
D367	Sum of topological distance between the vertices N and P
D413	Walk-returning count order-5
D477	Geary topological structure autocorrelation length-7 weighted by atomic polarizabilities
D478	Geary topological structure autocorrelation length-8 weighted by atomic polarizabilities
D532	Lowest eigenvalue from Burden matrix weighted by masses order-1
D533	Lowest eigenvalue from Burden matrix weighted by masses order-2
D534	Lowest eigenvalue from Burden matrix weighted by masses order-3
D535	Lowest eigenvalue from Burden matrix weighted by masses order-4
D564	Highest eigenvalue from Burden matrix weighted by masses order-1
D588	Highest eigenvalue from Burden matrix weighted by polarizabilities order-1
D674	Number of group total hydroxyl groups
D746	Number of group H attached to C0(sp3) no X attached to next C
NPR1	Normalized Principal Moment of Inertia 1
SpherocityIndex	An anisometry descriptor, it varies from zero for flat molecules, to one for totally spherical molecules
PBF	Plane of best fit

Table S5: Optimized hyperparameters of the ML methodologies in the QSAR workflow after 5-fold cross validation.

ML methodology	Hyperparameters	Optimized hyperparameters
J48	Maximum depth	10
	Minimum sample split	7
XGBoost	Maximum tree depth	11
	Learning rate (eta)	0.25
	alpha	4
	lambda	5
	gamma	0
	Minimum child weight	1
	Subsampling rate	0.8
kNN	Number of neighbours (*k*)	9

Table S6: Y-randomization results, expressed as accuracy and MCC, when the shuffled-endpoint XGBoost models (trained using the optimized hyperparameters) were applied on the original test set.

Y-Randomization	Accuracy	MCC
# 1	0.514	-0.003
# 2	0.538	0.051
# 3	0.494	-0.048
# 4	0.517	0.004
# 5	0.512	-0.007

Case study: Validation with experimental data

Table S7: Experimental results of Ames test available on databases for the case study compounds.

Reagent [CAS]	ECHA (according to TG471, y/n/equivalent)	Comptox	Metabolic activation
HMDS [999-97-3]	Negative (y)	Negative	With and without
PDMS [107-46-0]	Negative (equivalent)	Negative	With and without
TMMS [1185-55-3]	Negative (y)	Negative	With and without
TCMS [75-79-6]	Negative (equivalent)	Negative	With and without
TEOS [78-10-4]	Negative (equivalent)	Negative	With and without
TMOS [681-84-5]	Negative (y)	Negative	With and without
MTES [2031-67-6]	Negative (y)	Negative	With and without
VTMS [2768-02-7]	NA	Negative	With and without

References

1. Labute, P. A widely applicable set of descriptors. *J. Mol. Graph. Model.* **18**, 464–477 (2000).
2. Sauer, W. H. B. & Schwarz, M. K. Molecular shape diversity of combinatorial libraries: A prerequisite for broad bioactivity. *J. Chem. Inf. Comput. Sci.* **43**, 987–1003 (2003).
3. Song, C. *et al.* A polymer genome approach for rational design of poly(aryl ether)s with high glass transition temperature. *J. Mater. Chem. A* **11**, 16985–16994 (2023).
4. RDKit: Open-source cheminformatics. rdkit.Chem package. https://www.rdkit.org/docs/source/rdkit.Chem.html (2025).
5. Wicker, J. G. P. & Cooper, R. I. Will it crystallise? Predicting crystallinity of molecular materials. *CrystEngComm* **17**, 1927–1934 (2015).
6. Bonchev, D. & Trinajstić, N. Information theory, distance matrix, and molecular branching. *J. Chem. Phys.* **67**, 4517–4533 (1977).
7. RDKit: Open-source cheminformatics. RDKit SpacialScore module. https://rdkit.org/docs/source/rdkit.Chem.SpacialScore.html.
8. Landrum, G. A. What are the VSA Descriptors? *RDKit blog* https://greglandrum.github.io/rdkit-blog/posts/2023-04-17-what-are-the-vsa-descriptors.html (2024).
9. RDKit: Open-source cheminformatics. RDKit List of Available Descriptors. https://www.rdkit.org/docs/GettingStartedInPython.html#list-of-available-descriptors.
10. Ertl, P., Rohde, B. & Selzer, P. Fast Calculation of Molecular Polar Surface Area as a Sum of Fragment-Based Contributions and Its Application to the Prediction of Drug Transport Properties. *J. Med. Chem.* **43**, 3714–3717 (2000).
11. RDKit: Open-source cheminformatics. RDKit: Descriptors Namespace Reference. https://www.rdkit.org/docs/cppapi/namespaceRDKit_1_1Descriptors.html (2025).
12. RDKit: Open-source cheminformatics. rdkit.Chem.Fragments module. https://www.rdkit.org/docs/source/rdkit.Chem.Fragments.html (2025).
13. Firth, N. C., Brown, N. & Blagg, J. Plane of best fit: A novel method to characterize the three-dimensionality of molecules. *J. Chem. Inf. Model.* **52**, 2516–2525 (2012).
14. Hong, H. *et al.* Mold2, molecular descriptors from 2D structures for chemoinformatics and toxicoinformatics. *J. Chem. Inf. Model.* **48**, 1337–1344 (2008).
15. Todeschini, R. & Consonni, V. *Molecular Descriptors for Chemoinformatics*. vol. 2 (Wiley, 2009).
